# Early prevention of diabetes microvascular complications in people with hyperglycaemia in Europe. ePREDICE randomized trial. Study protocol, recruitment and selected baseline data

**DOI:** 10.1371/journal.pone.0231196

**Published:** 2020-04-13

**Authors:** Rafael Gabriel, Nisa Boukichou Abdelkader, Tania Acosta, Aleksandra Gilis-Januszewska, Ricardo Gómez-Huelgas, Konstantinos Makrilakis, Zdravko Kamenov, Bernhard Paulweber, Ilhan Satman, Predrag Djordjevic, Abdullah Alkandari, Asimina Mitrakou, Nebojsa Lalic, Stephen Colagiuri, Jaana Lindström, Jesús Egido, Andrea Natali, J. Carlos Pastor, Yvonne Teuschl, Marcus Lind, Luis Silva, Ruy López-Ridaura, Jaakko Tuomilehto, On behalf of the e-PREDICE Consortium

**Affiliations:** 1 Departamento de Salud Internacional, Escuela Nacional de Sanidad, Instituto de Salud Carlos III, Madrid, Spain; 2 World Community for Prevention of Diabetes Foundation (WCPD), Madrid, Spain; 3 EVIDEM CONSULTORES, Madrid, Spain; 4 Asociación para la Investigación y Prevención de la Diabetes y Enfermedades Cardiovasculares (PREDICOR), Madrid, Spain; 5 Department of Public Health. Universidad del Norte, Barranquilla, Colombia; 6 Uniwersytet Jagiellonski, Collegium Medicum, Krakow, Poland; 7 Fundación FIMABIS. Servicio Andaluz de Salud (SAS), Malaga, Spain; 8 National and Kapodistrian University of Athens, Athens, Greece; 9 University Multi-Profile Hospital for Active Treatment Alexandrovska EAD, Sofia, Bulgaria; 10 Gemeinnuetzige Salzburger Landeskliniken Betriebsgesellschaft, (SALK) Salzburg, Austria; 11 Istanbul University, Istanbul, Turkey; 12 General Hospital Medical System Beograd—MSB Belgrade Serbia, Beograd, Serbia; 13 Dasman Diabetes Research Institute, Kuwait, Kuwait; 14 Alexandra Hospital, University of Athens, Athens, Greece; 15 Faculty of Medicine, University of Belgrade, Belgrade, Serbia; 16 The University of Sydney, Boden Institute of Obesity, Nutrition, Exercise & Eating Disorders, Sydney, Australia; 17 National Institute for Health and Welfare, Helsinki, Finland; 18 Renal, Vascular and Diabetes Research Laboratory, Spanish Biomedical Research Centre in Diabetes and Associated Metabolic Disorders (CIBERDEM), Instituto de Investigación Sanitaria Fundación Jiménez Díaz, Universidad Autónoma, Madrid, Spain; 19 Department of Internal Medicine, Universita di Pisa, Pisa, Italy; 20 Instituto Universitario de Oftalmobiología Aplicada (IOBA), Universidad de Valladolid, Hospital Clínico Universitario, Valladolid, Spain; 21 Department for Clinical Neurosciences and Preventive Medicine, Danube University Krems, Krems, Austria; 22 Västra Götalands Läns Landsting, Gothenburg, Sweden; 23 Instituto Nacional de Salud Púbica, Cuernavaca, México; 24 University of Helsinki, Helsinki, Finland; 25 King Abdulaziz University, Jeddah, Saudi Arabia; Weill Cornell Medical College in Qatar, QATAR

## Abstract

**Objectives:**

To assess the effects of early management of hyperglycaemia with antidiabetic drugs plus lifestyle intervention compared with lifestyle alone, on microvascular function in adults with pre-diabetes.

**Methods:**

Trial design: International, multicenter, randomised, partially double-blind, placebo-controlled, clinical trial.

**Participants:**

Males and females aged 45–74 years with IFG, IGT or IFG+IGT, recruited from primary care centres in Australia, Austria, Bulgaria, Greece, Kuwait, Poland, Serbia, Spain and Turkey.

**Intervention:**

Participants were randomized to placebo; metformin 1.700 mg/day; linagliptin 5 mg/day or fixed-dose combination of linagliptin/metformin. All patients were enrolled in a lifestyle intervention program (diet and physical activity). Drug intervention will last 2 years. Primary Outcome: composite end-point of diabetic retinopathy estimated by the Early Treatment Diabetic Retinopathy Study Score, urinary albumin to creatinine ratio, and skin conductance in feet estimated by the sudomotor index. Secondary outcomes in a subsample include insulin sensitivity, beta-cell function, biomarkers of inflammation and fatty liver disease, quality of life, cognitive function, depressive symptoms and endothelial function.

**Results:**

One thousand three hundred ninety one individuals with hyperglycaemia were assessed for eligibility, 424 excluded after screening, 967 allocated to placebo, metformin, linagliptin or to fixed-dose combination of metformin + linagliptin. A total of 809 people (91.1%) accepted and initiated the assigned treatment. Study sample after randomization was well balanced among the four groups. No statistical differences for the main risk factors analysed were observed between those accepting or rejecting treatment initiation. At baseline prevalence of diabetic retinopathy was 4.2%, severe neuropathy 5.3% and nephropathy 5.7%.

**Conclusions:**

ePREDICE is the first -randomized clinical trial with the aim to assess effects of different interventions (lifestyle and pharmacological) on microvascular function in people with pre-diabetes. The trial will provide novel data on lifestyle modification combined with glucose lowering drugs for the prevention of early microvascular complications and diabetes.

**Registration:**

- ClinicalTrials.Gov Identifier: NCT03222765

- EUDRACT Registry Number: 2013-000418-39

## Introduction

The World Health Organization (WHO) defines ‘Intermediate hyperglycaemia’ (pre-diabetes) as 2 h post 75 g oral glucose tolerance test (OGTT) plasma glucose (2h PG) levels of 7.8–11.0 mmol/l (Impaired Glucose Tolerance–IGT-) or fasting plasma glucose (FPG) of 6.1–6.9 mmol/l with 2 h PG <7.8 mmol/l (Impaired Fasting Glucose–IFG-) [1]. Progression towards manifest diabetes type 2 (T2D) approximate ranges between 5 and 10% per year: 4–6% for isolated IGT; and 6–9% for IFG plus IGT [2–4].

People with pre-diabetes have a significantly increased risk for microvascular disease: nephropathy, neuropathy, retinopathy, as well as macrovascular disease and mortality [5–11]. In addition, it has been shown that people with pre-diabetes have generalized microvascular dysfunction and sequelae representing end-organ damage typical of diabetes [12]. Based on such evidence it can be proposed that the definition of pre-diabetes should not only be centered on glucose abnormalities, but should incorporate also the microvascular involvement among its criteria [13, 14].

A recent meta-analysis of published clinical trials on the prevention of T2D have showed the benefits of lifestyle intervention in delaying the onset of T2D in patients with pre-diabetes [15]. In addition, several antidiabetic drugs such as acarbose, metformin, rosiglitazone, pioglitazone and liraglutide have shown benefit for prevention of diabetes in pre-diabetic individuals [16–20]. However, the evidence for the prevention of microvascular complications in people with pre-diabetes is scarce. Few prevention trials have evaluated the potential benefits of lifestyle or drug treatment on microvascular complications in pre-diabetes. Clinical evidence regarding the microvascular benefit of lifestyle or metformin in prediabetes was inconclusive in the DPP [21]. Recently, the Carmelina study has shown preliminary evidence on the benefits of linagliptin for prevention of microvascular impairment in type 2 diabetes [22]. To our knowledge thus far no trial has explored the extent to which microvascular function can be preserved, and hence complications prevented, when combining lifestyle interventions with early pharmacological treatment in people with pre-diabetes.

The specific aim of the ePREDICE trial is to assess the effects of early intensive management of hyperglycaemia with linagliptin, metformin or their combination, added to lifestyle intervention, compared with lifestyle intervention alone, on microvascular impairment (retinal, renal and peripheral nerves) in adults with pre-diabetes. This paper describes the study design, enrolment, and basic demographic, epidemiological and clinical characteristics of participants at baseline.

## Material and methods

### Study design

ePREDICE is a multicenter, International, independent, randomized, double-blind, placebo-controlled, clinical trial. Participants are randomised with equal probability to four parallel study arms: placebo, metformin, linagliptin, and fixed-dose combination of linagliptin/metformin. Lifestyle intervention (diet and physical activity) is delivered to all patients. The active intervention will last for 2 years and patients will be followed-up during an additional observational period of one year.

### Study population

#### Inclusion criteria

People with pre-diabetes selected from several clinical centers in Australia, Austria, Bulgaria, Greece, Kuwait, Poland, Serbia, Spain and Turkey.

Men and women aged 45–74 years with IFG: FPG 6.1 to 6.9mmol/l and OGTT 2h PG: <7.8mmol/L; or IGT: FPG <7.0mmol/L and OGTT 2h PG >_7.8 and <11.1mmol/L) or both conditions together.

*Exclusion criteria*. Type 1 diabetes; known or screen-detected T2D; use of any antidiabetic drug within the 3 months prior to inclusion, any previous cardiovascular event, stroke or revascularization procedure of any arterial territory; morbid obesity (Body Max Index>45); current renal replacement therapy; renal function impairment: estimated glomerular filtration rate (eGFR <60 ml/min/1.73m^2^); diagnosis of liver cirrhosis or chronic hepatitis; previous diagnosis of acute or chronic pancreatitis; elevation of pancreatic enzymes (amylase/Lipase) >3 times of the upper normal ranges; diagnosis of chronic heart failure (NYHA class III or higher); organ transplantation or waiting for organ transplantation; diagnosis of malignant neoplasm requiring chemotherapy, surgery, radiation or palliative therapy or end-stage or metastatic cancer; known hypersensitivity to trial products or related products; known use of non-prescribed narcotics or illicit drugs; simultaneous participation in any other clinical trial of an investigational agent; females of childbearing potential who are pregnant, breast-feeding or intend to become pregnant; dementia, mental disorder or evident cognitive impairment; unable to give written informed consent; chronic institutionalization (nursing/mental health home, hospital, prison) conditions impeding retinal assessment as cataract, ocular surgery in the previous month, concomitant intraocular treatment (retina or choroid), tropicamide allergy; complete amputation of one/ both hands or one/both feet and any other circumstance that in the investigator opinion could interfere with the trial initiation, visit schedules or procedures.

### Primary endpoint

Two-year change in the microvascular Index, defined as a linear combination of three variables: the retinal “Early Treatment of Diabetic Retinopathy Study (ETDRS) score, the urinary albumin to creatinine ratio (UACR) and the sudomotor index using the SUDOSCAN® device.

### Secondary endpoints

Incidence of diabetes; quality of life, cognitive function, depressive symptoms, fatty liver index, insulin sensitivity, beta-cell function, and biomarkers of inflammation and endothelial dysfunction in a subsample of participants.

### Safety endpoints

Incidence of serious adverse events (SAE) and incidence of adverse events of special interest related to specific drug treatment as hypoglycaemic episodes, neoplasms and pancreatitis.

### Intervention groups

Placebo (matched to linagliptin) twice a day plus lifestyle modification; metformin 1700 mg/day plus lifestyle modification; linagliptin 5 mg/day plus lifestyle modification; and fixed-dose combination of linagliptin 5 mg/day and metformin 1,700 mg/day plus lifestyle modification.

### Lifestyle intervention

All randomized patients, regardless of drug treatment, receive similar lifestyle intervention program, aimed at promoting several lifestyle habits simultaneously. The lifestyle intervention program contains informative as well as behavioural and motivational modules and consists of two individual -sessions and 12 group sessions (up to 15 participants per session). The program follows the process model for supporting lifestyle behaviour changes proposed by the EU-funded IMAGE project (http://www.image-project.eu/). Group-sessions are repeated every month during the first six months of the trial, and thereafter every three months. Individual sessions last for one hour and group sessions 1, 5 hours. The lifestyle goals are based on those of the Finnish Diabetes Prevention Study translated into food intake [23]. The informative modules of nutrition and physical activity encourage participants to increase consumption of vegetables, fruits and berries, legumes, whole-grain cereal, vegetable sources of fat and protein (nuts, legumes), olive or rapeseed oil, and fish over unhealthy food choices such as fast food, refined grains and sweet treats. The dietary intervention has been culturally adjusted to local conditions and food supply while retaining the same goals. Optionally, based on local centre resources, physical activity intervention was delivered by group coaching (two sessions per week starting at month-3), and facilitate access to local gyms and sport clubs; otherwise physical activity is strongly emphasized during the group sessions. The intervention is delivered by staff nurses or nutritionists or “coachers” previously trained by experts.

### Sample size

Since there is no published data on the efficacy of pharmacological treatment compared with lifestyle intervention to reduce microvascular dysfunction in pre-diabetes, the sample size estimation was challenging. We used feet skin conductance sudomotor index (ESC), a proxy of early peripheral neuropathy measured with the SUDOSCAN device to detect changes in small fibres of peripheral nerves earlier than the occurrence of clinical neuropathy, which takes a long time to develop [24].

SUDOSCAN calculates feet ESC automatically, without the influence of observer bias. The cardiovascular autonomic neuropathy risk score is derived from feet ESC values adjusted for age and Body Max Index. For sample size and power calculation we used 1-year changes in SUDOSCAN parameters collected in a previous pilot intervention study in pre-diabetic patients (data not published).Sample size and power calculations were performed with the validated public statistical software GRANMO, version 7.0 (https://www.imim.cat/ofertadeserveis/software-public/granmo/).

Based on a pilot study, we considered the most unfavourable outcome result as an absolute difference between drug and lifestyle intervention of 1.8 units in ESC, a common standard deviation of 12.1 in the whole population, follow-up dropout rate of 25%, correlation between two SUDOSCAN measurements 0.80, size ratio lifestyle alone group /drug group 1:3, two-sided test alpha risk 0.05, and beta risk 0.20. Using all these assumptions, the total calculated sample size is 896 individuals (224 individuals per arm). The statistical power of this sample to detect significant difference between lifestyle intervention alone and the three drug arms together for SUDOSCAN-ESC is 82%.

### Randomization procedure

Randomization was centralized and stratified by center with permuted block within each centre to ensure balance between treatment overtime and within centres. People were equally allocated to any of the four 4 different groups (p = 0.25). The design of the study was partially double-blinded, only between one of the active drug arms (linagliptin only) and the placebo arm, and single-blinded (only for the patient) for the arm with metformin alone and the fixed combination of metformin/linagliptin. Given the partially blinded design, we randomly varied block sizes to avoid predictability of treatment assignment and selection bias. A specific web-based, central, independent, randomization tool under OpenClinica® software was used for randomization. Random blocks were allocated to each centre as needed (dynamic allocation of blocks). The coordinating centre (EVIDEM) centrally generated the random allocation sequence and assigned participants to each intervention. Each clinical centre enrolled participants centrally assigned to each treatment group.

Several possible situations were foreseen in advance for randomization code breaking (unmasking): a) end of experimental treatment at month 24^th^of trial initiation; b) need of rescue therapy during follow-up if average of HbA1c>6.5% or fasting glucose ≥7.0 mmol/l or 2h post load glucose ≥11.1 mmol/l in two consecutive study visits; c) any serious adverse event (SAEs); d) voluntary drug discontinuation and/or withdrawn of the participant from the trial; and e) pregnancy in a female participant during the trial. Despite of unmasking or drug discontinuation, lifestyle intervention and outcome assessments are carried out as originally planned (intention to treat).

### Study procedures

#### Assessment of primary outcomes

Diabetic Retinopathy is graded according to the final ETDRS scale using digital fundus photographs. The photograph´s protocol was the one validated by the Joslin Vision Network (3 pictures covering posterior pole) performed under mydriasis with tropicamide, and carried out by certified photographers. Retinal images were sent to the reading centre at the IOBA (Eye Institute) of the University of Valladolid, Spain, and graded by trained ophthalmologists who were blinded to the participant´s treatment group assigned. Retinal findings were graded using the ETDRS [25].

Detection of distal small fiber nerve impairment was done measuring the peripheral nerve conductance by the proxy sudomotor function with the SUDOSCAN device, a non-invasive quantitative method based on the electrochemical reaction between sweat chlorides and stainless-steel electrodes in contact with the palm of the hands and soles of the feet. Results of nerve conductance are given in micro-siemens (μS) for hands and feet. SUDOSCAN can detect distal small fibre polyneuropathy with a sensitivity of >75%. The SUDOSCAN method has been validated for the detection of sudomotor dysfunction [24–26]. Early diabetic peripheral neuropathy is defined as a reduction of >1SD in the conductance values between two measurements.

Urine albumin and creatinine levels are measured from spot urine samples in the central laboratory Fundación Jiménez Díaz, Madrid. Microalbuminuria is defined as UACR of ≥30 mg/g; Clinical albuminuria is defined as UACR ≥300 mg/g. Glomerular filtration rate is estimated by the Modification of Diet in Renal Disease (MDRD-4) Study equation, and by the Chronic Kidney Disease Epidemiological Collaboration (CKD-EPI) equation [27].

The primary composite microvascular complication index is calculated using a linear combination of the ETDRS score, the level of urinary albumin to creatinine ratio, and the SUDOSCAN index.

#### Assessment of secondary endpoints

Self-perceived quality of life with the EQ-5D-5L instrument; symptoms of neuropathy are measured with the Michigan Neuropathy Screening Instrument; symptoms of depression are recorded with the Hamilton Rating Scale for Depression (HAM-D) and the Mini-International Neuropsychiatric Interview (MINI) [28–31]. Cognitive function is measured with the Montreal Cognitive Assessment (MOCA), Trail making test A and B and Digit-Span forward and backward [32, 33].

Insulin secretion rates and β-cell glucose sensitivity are estimated by the slope of the insulin secretion/plasma glucose dose-response by C-peptide deconvolution by OGTT modelling [34]. Finally, in a random subsample of participants, the endothelial function with the Endo-PAT^®^device, and novel biomarkers related to inflammation, microvascular function and non-alcoholic fatty liver disease (NAFLD) will be also measured [35,36].

In addition to outcome assessment, at baseline each participant undergone a complete medical interview, a physical activity assessment with the RPAQ questionnaire, and dietary habits assessment with a food frequency questionnaire combining questions from two validated questionnaire [37–39].

Physical examination included body weight measurement (0.1 kg precision with calibrated balance) and height (0.5 cm scale precision) in light indoor clothing without shoes; heart rate was measured during one minute and blood pressure recorded twice with a mercury sphygmomanometer in both seated and standing position; waist and hip circumferences were measured with a plastic tape (0.1 cm precision).

Biochemical determinations included an oral glucose tolerance test (OGTT) carried out according to the WHO recommendations. A 300 ml test solution containing 75g of anhydrous glucose and 1.6 g of citric acid was used. The OGTT started after 12 hours of fasting. Venous blood samples were obtained after the ingestion of the glucose solution at min 0, 30, 60, 90 and 120. Samples were drawn into fluoridated tubes and centrifuged within 30 minutes. Plasma glucose is determined locally in all samples with the HEMOCUE® system. The aliquot samples from all point-time OGTT were shipped to the central laboratory for insulin and c-peptide assays. Also HbA1c, serum total cholesterol (T-C), high-density lipoprotein (HDL-C), triglycerides (TG), low density lipoprotein (LDL-C), AST, ALT, GGT, ALP, amylase, serum creatinine and uric acid concentrations were determined by usual standard methods at each local laboratory. Complete assessments are repeated at month 24th.

The protocol is available at www.epredice.eu and registred at https://clinicaltrials.gov/ct2/show/NCT03222765. For data entry and quality control, a specific web-based system under open-source software Open Clinica® was developed (https://community.openclinica.com).

### Statistical analysis

This paper provides data on the screening, randomization and intention-to-treat processes. Baseline descriptive results are only provided for people randomized and who accepted the treatment assigned. The only comparative data provided are between the whole group of patients who initiated treatment and those who refused to participate. Comparisons by treatment arm are not provided in order to preserve randomization concealment, as the trial is still ongoing. For this specific paper only descriptive statistics (mean, SD, percentages and 95%CI) are reported. Comparisons between randomised & treated vs randomised & not-treated, after randomization were done with the t-test for two independent samples and for comparisons of proportions were done with the Pearson Chi-Squared test. Blinding of the randomised treatments are maintained until the final database is closed and released for statistical analyses, after all participants have completed the 24-month visit or have earlier discontinued study therapy.

No analyses of unmasked or between-group data will be performed before the database is closed or released. All statistical analyses are performed by an independent statistics group. Analysis of efficacy will be done both by intention-to treat (as randomised), and per protocol.

The primary analysis will compare complications in all three antidiabetic therapies combined (metformin, linagliptin, and linagliptin with metformin), to complications in the lifestyle only arm, to see whether further glucose lowering in the prediabetic state by antidiabetic therapies reduces diabetic complications. It will also assess whether any of the active pharmacologic therapies separately has a superior effect compared to lifestyle intervention.

Analysis will be performed using Mann-Whitney U-test (two-sided, significance level 0.05) or alternatively analysis of covariance (ANCOVA) in case adjustment for baseline variables is required between the 3 active arms combined and the lifestyle arm. The analyses will be performed on the intention to treat basis, with adjustments for baseline variables significantly differing between the groups and significantly related to the outcome variable.

An independent external Data Safety and Monitoring Committee (DSMC) was constituted to oversee safety and to perform ongoing safety surveillance at pre-defined time points. The DSMC evaluates all relevant safety information received, and has access to un-blinded data. SAEs are assessed and classified by the DSMC. SAEs will be then encoded using the Medical Dictionary for Regulatory Activities (Med DRA version 12.1) system. All SAEs are collected, assessed and recorded by the local investigator as to the severity, possible relationship to the study medication and onset. This includes laboratory abnormalities, if considered an SAE by the investigator. All SAEs will be tabulated in the statistical report as total, by severity adverse events and if related to the study medication.

### Ethical issues

Prior to any trial-related activity, the investigators gave verbal and written information about the trial in a form that the participant could read and understand. A voluntary, personally dated written informed consent form was signed by all study participants, prior to any trial-related activity was started (form attached). The informed consent sheet complied with the applicable regulatory European medical agency requirements, and adheres with the International Good Clinical Practice Guidelines, and the Helsinki Declaration.

The trial was registered in EUDRA-CT (ID 2013-000418-39) before the first patient was recruited and after in the ClinicalTrials.gov (ID: NCT03222765). The main reasons for delay in registering this study was Stopped during more than one year because financial problems and bureaucracies with the EU. The participating clinical centres entered the study at different times. At the very beginning the trial was only initiated in Spain. At this stage we were unable to predict if the trial would stay only as a pilot trial, and how many of participating centres identified in the EU proposal would be able to overcome these financial and administrative barriers. During this process the coordinating centre and the CRO changed two times, 4 partners withdrew the European Consortium, one of the drug companies compromised to donate medication withdrew the consortium, and the EU delayed the funds during more than one year and a half until all administrative local problems described were overcome. So we decided do not register the trial in clinicaltrials.gov until we were sure the trial will continue, avoiding early removal, continuous changes in the registration process, and unnecessary works. The authors confirm that all ongoing and related trials for this drug/intervention are registered.

The clinical trial is currently ongoing, patients and data are being managed in a masked fashion. Data cannot be made publicly available since data would compromise confidentialty and might reveal identity or location of participants. Additionaly, public availability of data would be in violation of the Spanish Organic Law 15/1999 of protection of personal data (consolidated text 5/3/2011) and the European Law (EU) 2016/679 from European Parlament and European Council of 27 of April 2016 about Data Protection (RGPD).

The trial protocol was approved by all national Medicine Agencies and local ethic committees of participating centres:

- Comité Ético de Investigación Clínica del Hospital Universitario La Paz, Madrid, Spain. Approval No. 3850 (01/02/2013).
- Hellenic Republic Ministry of Health National Ethics Committee, Greece. Approval No. 74/00-01/14 (15/12/2014).
- The Dean office, Istanbul Medical Faculty Ethical Board for Clinical Trials, Turkey.-pproval No. 2014/495 (10/04/2014).
- Etickog Odbora. Beograd, Republike Srbije, Serbia. Appoval No. 61/1 (06/04/2013).
- Ethics Committee of the Faculty of Medicine, University of Belgrade, Serbia. Approval No. 29/III-10(07/03/2013).
- Komisji Bioetycznej UJ (Uniwersytetu Jagiellonskiego), Poland. Appoval No. BET/185/L/2014(26/06/2014).
- Executive Officer of Ethics Review Committee (RPAH) & Human Research Ethics Committee (HREC), Australia. Approval No. X13-0046 & HREC/13/RPAH/65 (12/03/2014).
- комисия по етика "Александровска" ЕАД София, България, Bulgaria. Approval No. KИ-213/18.03.15 (18/03/2015).
- Die Ethikkommission für das Bundesland Salzburg. "Landeskrankenhaus Salzburg-
- Universitätsklinikum der PMU, Universitätsklinik für Innere Medizin I", Austria. Approval No. 415-E/1649/10-2014 (08/07/2014).

All study participants gave their written informed consent prior to the participation in the study. The data Access Committee of Consejería de Sanidad de la Comunidad de Madrid”address: c/Plaza Carlos Trías Bertrán n°7 (Edif. Sollube) Madrid 28020; (protecciondedatos.sanidad@madrid.org) could consider those request that do not involve any conflict with these legal regulations. Any acceptable request will be processed and alsoevaluated by the ePREDICE Steering Committee.

The funders had no role in study design, data collection and analysis, decision to publish, or preparation of the manuscript.

## Results

Enrolment of the first patient first visit was on March 12, 2015, and the last patient first visit was done on July 18, 2018 and the central database closed for this specific analysis. Recruitment and clinical follow-up is still ongoing.

**Fig 1:**
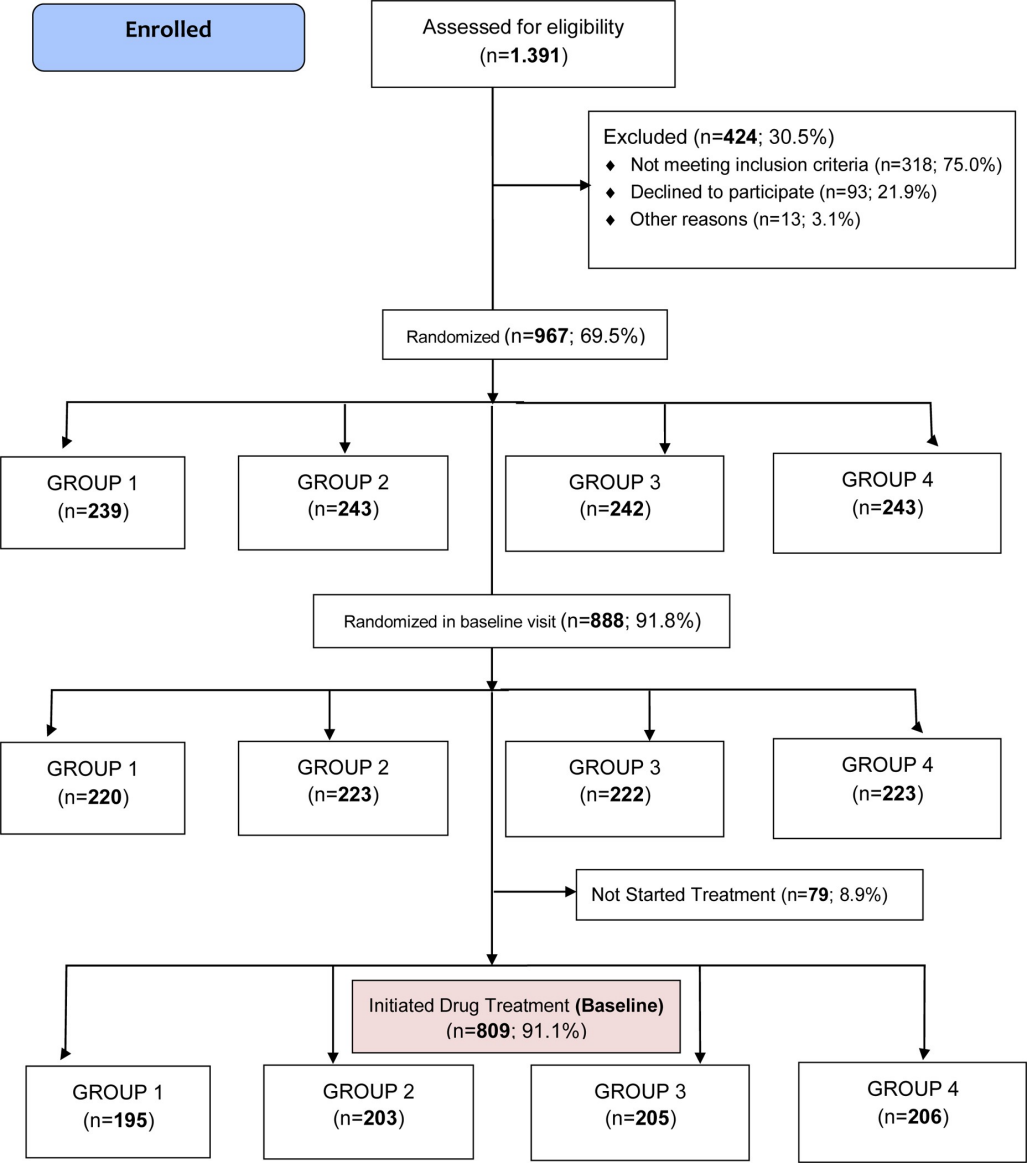
ePREDICE CONSORT flow diagram baseline.

Fig 1 shows the study population flow-diagram. One thousand three hundred ninety one potentially eligible adult individuals were screened using the standard OGTT; 424 (30.5%) were excluded after screening: 318 (75%) out of them because did not meet the inclusion criteria; 93 (21.9%) declined to sign the informed consent and 13 patients (3.1%) because other reasons. Nine hundred and sixty seven patients (69.5% of those screened) were randomized (ITT population) and 809 out of them (83.7%) started the assigned treatment. The flow-chart shows also the number of patients initially allocated to each treatment group, and the number of those who accepted and started the assigned treatment by study group as by July 18, 2018. The 92 randomized participants finally not included were allocated as follow: 27 to group 1; 24 to group 2; 20 to group 3 and 21 to group 4. But the new flow-chart the 79 randomized participants finally not included were allocated as follow: 25 to group 1; 20 to group 2; 17 to group 3 and 17 to group 4. The sample was quite well balanced among all four groups, and no statistical differences were observed in the allocation rate. The study is ongoing.

Table 1 shows the number and percentage of enrolled participants by centre by July 18, 2018. In Madrid, Spain a total of 323 were randomized and started treatment (39.9% of the total final sample). The corresponding number of people randomized and treated by center was 124 (15.3%) in Krakow, Poland; 84 (10.4%) in Malaga, Spain; 67 (8.3%) in Athens (NK), Greece; 76 (9.4%) in Sofia, Bulgaria; 55 (6.8%) in Salzburg, Austria; 36 (4.4%) in Istanbul, Turkey; 44 (5.4%) in Belgrade (MSB), Serbia; Ongoing (NA) in Kuwait; Ongoing (NA) in Athens (AH), Greece; Ongoing (NA) in Belgrade (UB), Serbia and Ongoing (NA) in Sydney, Australia.

**Table 1:** Number of people randomized and initiated treatment by study site in the ePREDICE trial. ^1^

Location	Randomized n(%)	Initiated the allocated study treatment n(%)
**Madrid, Spain**	349 (36.1)	323 (39.9)
**Krakow, Poland**	134 (13.9)	124 (15.3)
**Málaga, Spain**	90 (9.3)	84 (10.4)
**Athens (NK), Greece**	80 (8.3)	67 (8.3)
**Sofia, Bulgaria**	79 (8.2)	76 (9.4)
**Salzburg, Austria**	63 (6.5)	55 (6.8)
**Istanbul, Turkey**	49 (5.1)	36 (4.4)
**Belgrade (MSB), Serbia**	44 (4.6)	44 (5.4)
**Kuwait**	36 (3.7)	-
**Athens (AH), Greece**	17 (1.8)	-
**Belgrade (UB), Serbia**	17 (1.8)	-
**Sydney, Australia**	9 (0.9)	-
**TOTAL**	**967 (100)**	**809 (100)**

1) ePREDICE denotes: Early Prevention of Diabetes Complications in People with Hyperglycaemia in Europe.

* **NK:** National and Kapodistrian University of Athens. Greece.

* **MSB:** Medical System Beograd. Belgrade, Serbia.

* **AH:** Alexandra Hospital. University of Athens. Greece.

* **UB:** Faculty of Medicine, University of Belgrade. Serbia.

Table 2 shows preliminary data and compares means and standard deviations for key demographic characteristics and major cardio-metabolic risk factors between people randomized who started treatment (n = 809), and those randomized who did not started treatment (n = 79). No statistical differences were observed between the two groups for any of the variables analysed.

**Table 2:** Demographics and the major cardiovascular risk factors at baseline of people randomized in the ePREDICE trial (treated and non-treated; Intention-To-Treat population).

Key variables	Randomized Not Treated (n = 79)	Randomized Treated (n = 809)	P-value
**Females (%)**	50 (63.3)	470 (58.1)	**0.371**
**Smoking every day (%)**	11 (13.9)	116 (14.3)	**0.636**
**Family history of diabetes (%)**	26 (32.9)	321 (39.7)	**0.485**
**Use of antihypertensive drugs**	26 (32.9)	325 (40.2)	**0.208**
**Physical activity (30 min or more every day)**	37 (46.8)	415 (51.3)	**0.449**
**Vegetables, fruits or berries consumption (every day)**	42 (53.2)	475 (58.7)	**0.340**
**Age, years**	59.4 (7.8)	58.5 (7.6)	**0.327**
**BMI (kg/m**^**2**^**)**	30.8 (5.2)	30.6 (5.0)	**0.729**
**Waist circumference in males (cm)**	105.4 (12.2)	105.8 (12.0)	**0.594**
**Waist circumference in females (cm)**	98.9 (13.3)	99.3 (11.7)	**0.594**
**Systolic blood pressure (mmHg)**	130 (15.7)	132 (17.2)	**0.372**
**Diastolic blood pressure (mmHg)**	78 (8.9)	82 (10.7)	**0.150**
**Fasting plasma glucose (mmol/L)**	6.4 (0.4)	6.4 (0.5)	**0.323**
**2-hour plasma glucose (mmol/L)**	7.9 (1.7)	8.1 (1.7)	**0.382**
**Serum total cholesterol (mmol/L)**	5.1 (1.6)	5.3 (1.1)	**0.219**
**Serum HDL cholesterol (mmol/L)**	1.4 (0.3)	1.4 (2.7)	**0.930**
**Serum LDL cholesterol (mmol/L)**	3.2 (0.8)	3.3 (0.9)	**0.202**
**Serum triglycerides (mmol/L)**	1.5 (1.6)	1.5 (0.9)	**0.940**
**HbA1c (%)**	5.8 (0.3)	5.8 (0.4)	**0.545**
**eGFR CKD-EPI (mL/min per 1.73 m**^**2**^**)**	93.7 (7.4)	93.8 (9.5)	**0.884**
**FINDRISC Score**	13.3 (4.1)	13.8 (4.1)	**0.262**

For categorical variables % is presented.

For continuous variables Mean (SD) are presented.

BMI: body mass index;HbA1c: glycated haemoglobin; eGFR CKD-EPI: estimated glomerular filtration rate based on Chronic Kidney Disease Epidemiology Collaboration; FINDRISC: Finnish Diabetes Risk Score.

Table 3 shows preliminary data the percentages of relevant clinical abnormalities at baseline. Thirty four people (4.2%) shown DR>14 according to ETDRS; 43 people (5.3%) had severe peripheral neuropathy according to SUDOSCAN criteria (feet ESC <50 μS or hand ESC<40 μS), and 46 people (5.7%) had nephropathy at baseline with albuminuria levels >30 mg/dl.

**Table 3:** Frequency of relevant health conditions at baseline in randomized (treated and not-treated individuals; Intention-To-Treat population).

Health Condition	Randomized Not Treated (%) (n = 79)	Randomized Treated (%) (n = 809)	P-value
**Glycaemia categories**			**0.311**
**Isolated IGT**	16 (20.3)	226 (27.9)	
**Isolated IFG**	36 (45.6)	317 (39.2)	
**IGT+ IFG combined**	27 (34.2)	266 (32.9)	
**Hypertension (SBP ≥ 140 mmHg or DBP ≥ 90 mmHg or antihypertensive drug use)**	40 (50.6)	486 (60.1)	**0.103**
**Hypercholesterolemia (Serum total cholesterol ≥ 200 mg or lipid lowering drug use)**	51 (64.6)	536 (66.3)	**0.761**
**Overweight (BMI 25–29 kg/m^2^)**	22 (27.8)	223 (27.6)	**0.957**
**Obesity (BMI ≥ 30 kg/m^2^)**	41 (51.9)	411 (50.8)	**0.853**
**Abdominal obesity (Males: WC ≥ 94 cm)**	25 (37.3)	301 (42.9)	**0.625**
**Abdominal obesity (Female: WC ≥ 88 cm)**	42 (62.7)	401 (57.1)	**0.625**
**Diabetic Retinopathy (ETDRS > 14)**	0	34 (4.2)	NA
**Severe neuropathy (feet ESC [μS]<50 or hands ESC [μS]<40)**	2 (2.5)	43 (5.3)	**0.282**
**Nephropathy (Albumin > 30 mg/dl)**	2 (2.5)	46 (5.7)	**0.237**

IGT: Impaired Glucose Tolerance; IFG: Impaired Fasting Glucose; SBP: systolic blood pressure; DBP: diastolic blood pressure; BMI: body mass index; WC: waist circumference; ETDRS: Early Treatment Diabetic Retinopathy Scale; NA: not applicable.

Data from Kuwait (36 patients), Athens -AH- (17 patients), Belgrade -UB- (17 patients) and Sydney (9 patients) are incomplete or unavailable at the moment. Therefore, these patients were not included in the analyses performed.

## Discussion

ePREDICE is an investigator-driven, independent clinical trial, assessing effects of different glucose lowering drugs combined with lifestyle counselling on preservation of microvascular function and early prevention of retinopathy, nephropathy and peripheral neuropathy in pre-diabetic people. The trial is particularly innovative for the assessment of small nerve impairment by using the sudomotor function with the Sudoscan device.

Microvascular complications appear at early stage of the disease, and are suitable as major outcome when trying to prevent complications of hyperglycaemia. Our hypothesis is that intensive and early treatment combining safe antidiabetic drugs with lifestyle intervention is more effective than intensive lifestyle intervention alone in preventing microvascular complications in adults with non-diabetic hyperglycaemia range. The use of a particular antidiabetic drug is based on its capacity to control glycaemia, and the likelihood to produce long-term benefits, which is the key issue in pre-diabetes. We aim to investigate if the early and intensive multifactorial treatment could challenge the current standard approach to pre-diabetes, using pharmacological treatment as co-adjuvant to the lifestyle interventions. We also aim to see to what extent, a more intensive preventive treatment can reduce the progression from pre-diabetes to diabetes. Previous studies have shown that lifestyle intervention and pharmacotherapy reduces the risk of progression in people with IGT, but results among people with IFG have been inconclusive. Our study can throw light to this open question [40, 41].

The trial´s recruitment process was quite efficient with a 30.5% rate of screening failure, which is lower than those reported by other similar trials in the field [17]. The rate of acceptance of drug treatment (91.1%) is considered very high for a primary prevention trial with drugs and lifestyle combined, where only 50% of eligible people invited usually accept [17]. The main reasons for drug treatment refusal in primary prevention trials of chronic such as pre-diabetes including ours is the unawareness of suffering a health problem without symptoms, and the unwillingness to sign the informed consent or taking a masked drug. However, the similar socio-demographic characteristics and clinical profile of those people randomized who rejected or accepted the drug treatment, allows ruling out any risk of differential bias towards the inclusion of people with either higher or lower risk than planned.

We have compared the distribution of key cardio-metabolic risk factors in the ePREDICE population with pre-diabetic people recruited by the DE-PLAN study, an European program of diabetes prevention using the Finnish Diabetes Risk Score (FINDRISC) for screening followed of an OGTT, in the same age-group (45–74 years), and which was previously conducted by several ePREDICE investigators at the same primary care settings [42]. Participants in the ePREDICE trial are similar in terms of socio-demographic, cardio-metabolic and lifestyle profile than the sub-sample of prediabetic people in the DE-PLAN. Pre-diabetic population in ePREDICE and DE-PLAN shown similar fasting-and 2h PG levels. Therefore, we can conclude that ePREDICE sample represent fairly well the adult pre-diabetic population who use public primary care services across Europe [42].

Few studies have reported the prevalence of microvascular abnormalities in the pre-diabetic population, and less have used validated and standardized methods of assessment. The DPP trial used the same criteria for diabetic retinopathy evaluation (ETDRS and central grading), and reported a slightly higher prevalence of retinopathy (7.9%) in a sub-sample of prediabetic participants people who did not progressed to overt clinical diabetes after 3-year of follow-up [21]. The prevalence of peripheral neuropathy in pre-diabetic populations has been reported to be up to 15% depending of the method used, but data based on SUDOSCAN are scarce. The prevalence of severe neuropathy in our sample was 5.3%. A recent population study in Finnish men with high-risk of T2D reported up to 11% of mild or severe neuropathy [43, 44]. However, if we consider both severe and mild neuropathy together the prevalence raises up to 31% using the SUDOSCAN criteria. The prevalence of nephropathy in our study according to a level of albuminuria >30mg/dl was 5.7%, similar than the reported by Melsom et al in a sample of pre-diabetics from the US general population [45].

### Limitations

Our approach was similar to that used in other landmark trials of diabetes prevention which compared metformin to lifestyle intervention and both arms were also evaluated separately against placebo (US DPP). However, the DPP was unable to observe any benefit of lifestyle intervention or metformin on microvascular complications [21]. The original planning of ePREDICE duration is relatively short (2-years) due to the limitation of primary fund sources. Nevertheless, we hope that we will be able to monitor the status of the study participants for a longer period.

Our primary objective is to compare the primary outcome in all three anti-diabetic drug arms combined (metformin, linagliptin, and linagliptin/metformin) with the lifestyle (placebo) only arm. To increase trial´s power the three main outcomes have been combined in a composite endpoint.

### Potential impact

ePREDICE final results will have the potential of changing the current paradigm of early management of intermediate hyperglycaemia and to guide treatment decisions in pre-diabetes. The trial will permit the evaluation of effects of intensive treatment (lifestyle plus drug treatment) in pre-diabetes for the early prevention of complication associated with hyperglycemia. We also wish to characterize individuals who will develop early complications and to identify which complication appears first. The follow-up will enable us to assess also the cumulative incidence of various complications.

## Conclusions

The ePREDICE is the first randomized clinical trial assessing effects of different interventions (lifestyle and glucose lowering drugs together) on the prevention of microvascular complications and other outcomes in people with pre-diabetes. This approach can be also valuable to investigate the mechanisms how to reduce microvascular complications by glucose lowering drugs and lifestyle interventions. The ePREDICE study has proceeded well by collecting demographic, clinical information and complications associated with pre-diabetes at baseline.

## Supporting information

S1 Table Number of randomized and initiated treatment by study site. (DOC)

S2 Table Demographics and the major cardiovascular risk factors at baseline of people randomized in the ePREDICE trial (treated and non-treated; Intention-To-Treat population). (DOC)

S3 Table Frequency of relevant health conditions at baseline in randomized (treated and not-treated individuals; Intention-To-Treat population). (DOC)

S1 Checklist CONSORT 2010 checklist of information to include when reporting a randomised trial*. (DOC)

S1 Data (PDF)

S2 Data (PDF)

S3 Data (PDF)
