# Supplementary material for: Early prevention of diabetes microvascular complications in people with hyperglycaemia in Europe. ePREDICE randomized trial. Study protocol, recruitment and selected baseline data
Source: PLoS One. 2020 Apr 13;15(4):e0231196. doi: 10.1371/journal.pone.0231196 (PMC7153858; doi:10.1371/journal.pone.0231196)
Supplement: S1 Table — (DOC) [file pone.0231196.s001.doc]

**Table1. Number of people randomized and initiated treatment by study site in the ePREDICE trial^1^**

| **Location** | **Randomized**  **n(%)** | **Initiated the allocated study treatment n(%)** |
| --- | --- | --- |
| **Madrid, Spain** | 349 (36.1) | 323 (39.9%) |
| **Krakow, Poland** | 134 (13.9) | 124 (15.3%) |
| **Málaga, Spain** | 90 (9.3) | 84 (10.4%) |
| **Athens (NK), Greece** | 80 (8.3) | 67 (8.3%) |
| **Sofia, Bulgaria** | 79 (8.2) | 76 (9.4%) |
| **Salzburg, Austria** | 63 (6.5) | 55 (6.8%) |
| **Istanbul, Turkey** | 49 (5.1) | 36 (4.4%) |
| **Belgrade (MSB), Serbia** | 44 (4.6) | 44 (5.4%) |
| **Kuwait** | 36 (3.7) | - |
| **Athens (AH), Greece** | 17 (1.8) | - |
| **Belgrade (UB), Serbia** | 17 (1.8) | - |
| **Sydney, Australia** | 9 (0.9) | - |
| **TOTAL** | **967 (100)** | **809 (100)** |
| 1) ePREDICE denotes: Early Prevention of Diabetes Complications in People with Hyperglycaemia in Europe  * **NK:** National and Kapodistrian University of Athens. Greece  * **MSB:** Medical System Beograd. Belgrade, Serbia  * **AH:** Alexandra Hospital. University of Athens. Greece  * **UB:** Faculty of Medicine, University of Belgrade. Serbia | | |
