# Supplementary material for: Early prevention of diabetes microvascular complications in people with hyperglycaemia in Europe. ePREDICE randomized trial. Study protocol, recruitment and selected baseline data
Source: PLoS One. 2020 Apr 13;15(4):e0231196. doi: 10.1371/journal.pone.0231196 (PMC7153858; doi:10.1371/journal.pone.0231196)
Supplement: S2 Table — (DOC) [file pone.0231196.s002.doc]

**Table 2. Demographics and the major cardiovascular risk factors at baseline of people randomized in the ePREDICE trial (treated and non-treated;**

**Intention-To-Treat population)**

| **Key variables** | **Randomized**  **Not Treated**  **(n=79)** | **Randomized**  **Treated**  **(n=809)** | **P-value** |
| --- | --- | --- | --- |
| **Females (%)** | 50 (63.3) | 470 (58.1) | **0.371** |
| **Smoking every day (%)** | 11 (13.9) | 116 (14.3) | **0.636** |
| **Family history of diabetes (%)** | 26 (32.9) | 321 (39.7) | **0.485** |
| **Use of antihypertensive drugs** | 26 (32.9) | 325 (40.2) | **0.208** |
| **Physical activity (30 min or more every day)** | 37 (46.8) | 415 (51.3) | **0.449** |
| **Vegetables, fruits or berries consumption (every day)** | 42 (53.2) | 475 (58.7) | **0.340** |
| **Age, years** | 59.4 (7.8) | 58.5 (7.6) | **0.327** |
| **BMI (kg/m^2^)** | 30.8 (5.2) | 30.6 (5.0) | **0.729** |
| **Waist circumference in males (cm)** | 105.4 (12.2) | 105.8 (12.0) | **0.594** |
| **Waist circumference in females (cm)** | 98.9 (13.3) | 99.3 (11.7) | **0.594** |
| **Systolic blood pressure (mmHg)** | 130 (15.7) | 132 (17.2) | **0.372** |
| **Diastolic blood pressure (mmHg)** | 78 (8.9) | 82 (10.7) | **0.150** |
| **Fasting plasma glucose (mmol/L)** | 6.4 (0.4) | 6.4 (0.5) | **0.323** |
| **2-hour plasma glucose (mmol/L)** | 7.9 (1.7) | 8.1 (1.7) | **0.382** |
| **Serum total cholesterol (mmol/L)** | 5.1 (1.6) | 5.3 (1.1) | **0.219** |
| **Serum HDL cholesterol (mmol/L)** | 1.4 (0.3) | 1.4 (2.7) | **0.930** |
| **Serum LDL cholesterol (mmol/L)** | 3.2 (0.8) | 3.3 (0.9) | **0.202** |
| **Serum triglycerides (mmol/L)** | 1.5 (1.6) | 1.5 (0.9) | **0.940** |
| **HbA1c (%)** | 5.8 (0.3) | 5.8 (0.4) | **0.545** |
| **eGFR CKD-EPI (mL/min per 1.73 m^2^)** | 93.7 (7.4) | 93.8 (9.5) | **0.884** |
| **FINDRISC Score** | 13.3 (4.1) | 13.8 (4.1) | **0.262** |
| For categorical variables  % is presented  For continuous variables Mean (SD) are presented. | | | |

BMI: body mass index;HbA1c: glycated haemoglobin; eGFR CKD-EPI: estimated glomerular filtration rate based on Chronic Kidney Disease Epidemiology Collaboration; FINDRISC: Finnish Diabetes Risk Score
