# Supplementary material for: Early prevention of diabetes microvascular complications in people with hyperglycaemia in Europe. ePREDICE randomized trial. Study protocol, recruitment and selected baseline data
Source: PLoS One. 2020 Apr 13;15(4):e0231196. doi: 10.1371/journal.pone.0231196 (PMC7153858; doi:10.1371/journal.pone.0231196)
Supplement: S3 Table — (DOC) [file pone.0231196.s003.doc]

**Table 3. Frequency of relevant health conditions at baseline in randomized (treated and not-treated individuals; Intention-To-Treat population)**

| **Health Condition** | **Randomized**  **Not Treated (%)**  **(n=79)** | **Randomized**  **Treated (%)**  **(n=809)** | **P-value** |
| --- | --- | --- | --- |
| **Glycaemia categories** |  |  | **0.311** |
| **Isolated IGT** | 16 (20.3) | 226 (27.9) |  |
| **Isolated IFG** | 36 (45.6) | 317 (39.2) |  |
| **IGT+ IFG combined** | 27 (34.2) | 266 (32.9) |  |
| **Hypertension (SBP > 140 mmHg or DBP > 90 mmHg or antihypertensive drug use)** | 40 (50.6) | 486 (60.1) | **0.103** |
| **Hypercholesterolemia (Serum total cholesterol > 200 mg or lipid lowering drug use)** | 51 (64.6) | 536 (66.3) | **0.761** |
| **Overweight (BMI 25-29 kg/m^2^)** | 22 (27.8) | 223 (27.6) | **0.957** |
| **Obesity (BMI > 30 kg/m^2^)** | 41 (51.9) | 411 (50.8) | **0.853** |
| **Abdominal obesity (Males: WC > 94 cm)** | 25 (37.3) | 301 (42.9) | **0.625** |
| **Abdominal obesity (Female: WC > 88 cm)** | 42 (62.7) | 401 (57.1) | **0.625** |
| **Diabetic Retinopathy (ETDRS > 14)** | 0 | 34 (4.2) | NA |
| **Severe neuropathy (feet ESC [µS]<50 or hands ESC [µS]<40)** | 2 (2.5) | 43 (5.3) | **0.282** |
| **Nephropathy (Albumin > 30 mg/dl)** | 2 (2.5) | 46 (5.7) | **0.237** |

IGT: Impaired Glucose Tolerance; IFG: Impaired Fasting Glucose; SBP: systolic blood pressure; DBP: diastolic blood pressure; BMI: body mass index; WC: waist circumference; ETDRS: Early Treatment Diabetic Retinopathy Scale; NA: not applicable
