# Supplementary material for: Early prevention of diabetes microvascular complications in people with hyperglycaemia in Europe. ePREDICE randomized trial. Study protocol, recruitment and selected baseline data
Source: PLoS One. 2020 Apr 13;15(4):e0231196. doi: 10.1371/journal.pone.0231196 (PMC7153858; doi:10.1371/journal.pone.0231196)
Supplement: S2 Data — (PDF) [file pone.0231196.s006.pdf]

Title: **Early Prevention of Diabetes Complications in people with Hyperglycaemia in Europe: Study e-PREDICE**

Proposal acronym: **e-PREDICE**
Type of funding scheme: **Collaborative Project**
Work programme topics addressed: **HEALTH.2011.2.4.3-1: Investigator-driven clinical trials to reduce diabetes complications. FP7-HEALTH-2011-two-stage**

Co-Principal Investigators:

Prof. Rafael Gabriel

Prof. Jaakko Tuomilehto

SPONSOR:
EVIDEM CONSULTORES SL (EVIDEM)
Calle Marroquina 12 – 1ºA 28030 – Madrid

Legal representative**:**

Ana Roson Hernandez

FP7 Protocol identifying number: *279074*

EudraCT number: 2013-000418-39

Version Number: 5.1, June 12, 2018

*(**NOTE:** Any modification to the protocol should be annotated on the coversheet or in an appendix. The annotation should note the exact words that are changed, the location in the protocol, the date the modification was approved, and the date it became effective.)*

LOCAL PRINCIPAL INVESTGATORS SIGNATURE PAGE:

Study Title:	Early Prevention of Diabetes Complications in people with Hyperglycaemia in Europe: Study ePredice
Trial Number:	EC-GA No. 279074
EudraCT:	2013-000418-39
Protocol Version:	5.1
Protocol Date:	June 12 - 2018
Sponsor:	EVIDEM

Investigators Responsibilities
Prior to participation in the Early Prevention of Diabetes Complications in Europe, as the site principal investigator I understand that I must obtain written approval from my Institutional Review Board (IRB) or independent Ethics Committee (IEC). This approval must include my name and I must send a copy to EVIDEM along with the IRB/IEC approved Informed Consent document prior to study enrolment at my study site.

As site Principal Investigator, I must also:

1. Conduct the study in accordance with the study protocol, the signed Consortium Agreement, the principles of Good Clinical practices (GCP) guidelines as well as in accordance with the national, state and local laws of the appropriate regulatory authorities and declaration of Helsinki.
2. Ensure that all the study personnel are appropriately trained prior to any data collection.
3. Ensure that the study is not commenced until IRB/IEC approvals have been obtained.
4. Ensure that written Informed Consent is obtained from each patient prior to any data collection using the most recent IRB/IEC approved Patient Informed Consent Form.
5. Provide all required data and reports and agree to source document verification of study data with patients' medical records.
6. Allow EVIDEM personnel or its designees to inspect any documents pertaining to this clinical investigation.

Principal Investigator Signature
I have read and understood the content of the Early Prevention of Diabetes Complications in Europe study protocol and agree to abide by the requirements set forth in this document.

________________________________ ________________________________

Principal Investigator Name (print) Investigative Site (print)

________________________________ ________________________________

Principal Investigator Name (print)　　　　　　　　　　　　Date

EXECUTIVE SUMMARY

Background: A significant proportion of pre-diabetics, show macro and micro vascular complications associated with hyperglycaemia. Although many trials have demonstrated the efficacy of lifestyle and pharmaceutical interventions in diabetes prevention, no trial has evaluated the extent to which mid- and long-term complications can be prevented by early interventions on hyperglycaemia.

Aims: To assess the long-term effects on multiple complications of hyperglycaemia of early intensive management of hyperglycaemia with linagliptin, metformin or their combination added to lifestyle intervention (LSI) (diet and physical activity), compared with LSI alone in adults with non-diabetic intermediate hyperglycaemia (IFG, IGT or both).

Study Design: Investigator initiated (non-commercial), long-term, multi-centre, randomised, partially double blinded, placebo controlled, phase-IIIb clinical trial with prospective blinded outcome evaluation. Participants will be randomised to four parallel arms: 1) LSI + 2 placebo tablets/day; 2) LSI + 2 *Metformin* tablets of 850 mg/day; 3) LSI + 1 *Linagliptin* tablets of 5 mg/day and 1 placebo; 4) LSI + 2 tablets of a fixed-dose combination of *Linagliptin* 2.5mg and *Metformin* 850 /day. Active intervention will last for at least 2 years, and additional follow-up up to 3 years.

Setting and population: Males and Females with pre-diabetes (IFG, IGT or both) aged 45 to 74 years selected from primary care screening programs in 11 clinical centres from 8 countries: Austria, Bulgaria, Greece, Kuwait,, Poland, Serbia, Spain and Turkey.

Main Outcomes: The primary endpoint is a combined continuous variable: ***"the microvascular complication índex" (MCI***) composed by a linear combination of the Early Treatment Diabetic Retinopathy Study Scale (ETDRS) score (based on retinograms), the level of urinary albumin to creatinine ratio, and a measure of distal small fibre neuropathy (sudomotor test by SUDOSCAN), measured during baseline visit and at 24th and 36th month visits after randomisation. In addition, this project will include the evaluation of early novel serological biomarkers of systemic inflammation, early micro-vascular damage, non-alcoholic fatty liver disease, insulin sensitivity and insulin secretion, and measures of quality of life,and neuropsychological evaluation. Endothelial function measured by EndoPAT will be evaluated in a subset of participants .

Expected results: By evaluating the effect of aggressive treatments in pre-diabetes for the early prevention of diabetes complication, this project has the potential of changing the current paradigm of early management of hyperglycaemia. The ultimate goal is the development of a standardized core protocol for the early prevention of microvascular and other complications, impacting social cost as a result not only in health care, but also in disabilities at work.

TABLE OF CONTENTS

LOCAL PRINCIPAL INVESTGATORS SIGNATURE PAGE: ... 2
TABLE OF CONTENTS ... 4
1. AMENDMENTS HISTORY ... 6
1.1 List of amendments from version 2.1 to version 3.2 (05/July/2013). ... 6
1.2 List of amendments from version 3.2 to version 4.2 (02 January 2014) ... 9
1.3 List of amendments from version 4.2 to version 4.3 (02 March 2015) ... 11
1.4 List of amendments from version 4.3 to version 5.0 (11/26/2015) ... 11
1.5 List of amendments from version 5.0 to version 5.1 (12 June 2018) ... 11
ABBREVIATIONS ... 12
2. INTRODUCTION ... 13
2.1 Background Information ... 13
2.2 Rationale or Justification ... 15
3. OBJECTIVES AND PURPOSE ... 16
3.1 Primary objective: ... 16
3.2 Secondary objectives: ... 16
4. TRIAL DESIGN ... 16
4.1 Summary of Trial ... 16
4.2. Randomization and code breaking ... 19
4.3. Primary and Secondary Endpoints/Outcome ... 20
5. STUDY POPULATION ... 22
5.1 Inclusion Criteria ... 22
5.2 Exclusion Criteria ... 22
5.3 Subject Withdrawal Criteria: (terminating investigational product) ... 23
6. STUDY PROCEDURES ... 25
6.1 Lifestyle habits assessment ... 25
6.2 Non-invasive measurements ... 25
6.3 Biochemical measurements ... 25
6.4 Assessment of primary outcomes ... 26
6.5 Assessment of secondary endpoints ... 27
6.6 Informed Consent ... 28
7. STUDY VISITS ... 30
7.1 Screening and Eligibility Assessment: Enrolment and Baseline visits ... 30
7.2 Subsequent Assessments ... 34
7.3 End of Trial Assessment (Observational follow up after open experimental treatment) ... 36
8. INTERVENTIONS ... 38
8.1 Intervention Groups: ... 38
8.2 Lifestyle intervention ... 39
8.3 Drug intervention ... 43
9. STATISTICAL ANALYSIS AND ASSESSMENT OF EFFICACY ... 50
9.1 General considerations ... 50
9.2 Assessment of Efficacy: ... 50
10. ASSESSMENT OF SAFETY ... 53
10.1 Definitions ... 54
10.2 Causality and Expectedness ... 55
10.3 Procedures for Recording Adverse Events ... 55
10.4 Reporting Procedures for Serious Adverse Events ... 56
10.5 Type and Duration of Follow-Up of Subjects after Adverse Events ... 56
10.6 Safety analysis ... 56

11. SAMPLE SIZE CALCULATION 56
11.1 Power calculation 56
11.2 Adjustment of sample size and statistical power 58
12. DIRECT ACCESS TO SOURCE DATA/DOCUMENTS 60
13. QUALITY CONTROL AND QUALITY ASSURANCE 60
14. ETHIC, DEONTOLOGICAL AND REGULATORY CONSIDERATIONS 62
15. REFERENCES 65
Appendix A: Visit plan (eliminated) 68
Appendix B: Description of drug products characteristics released by the European Medicines Agency 69
Appendix C: Sample of informed consent document 69

1. AMENDMENTS HISTORY

1.1 List of amendments from version 2.1 to version 3.2 (05/July/2013).

1. **Drug Intervention**: Changes in the drug intervention of two arms: ***Group 3***: Change from one pill of 100 mg of Sitagliptin once a day to 2 pills of 50 mg twice a day, without the need of placebo pills. Sufficient evidence indicates equal effect of 100 mg per day either as a single dose or in two doses. ***Group 4:*** The dose of the metformin in the fixed combination of Sitagliptin/metformin changed from 1000 mg to 850 mg. The final fixed doe combination is Sitagliptin 50mg/Metformin 850mg.
    - Trial Summary:
    - Table 2: Intervention Groups:
    - Informed consent form
2. Define the design of the trial as a **partially double blinded**, instead of as single blinded, to clarify the actual blinding strategy. We called partially double blinded because placebo pills will only be prepared by MSD with an identical shape of the active sitagliptin 50 mg pill. Placebo pills will be provided blinded to the arm that receives only life-style intervention (group 1). For this arms and Group 3 arm (sitagliptin active drug 50mg twice a day, the trial is totally double blinded. However, for the other two arms only the bottles will be identical (as for all four arms) but the shape and colour of the pills will be different and unique with the possibility of being identified, at least by the physician in charge. Therefore, for these two groups, the blindness is only partially and more single (only the patient) than double blinded.
    - *Section 2.2 Rationale and Justification, Section 4.1 Summary of Trial, Section 4.2. Randomization and code breaking and Section 8.3 Drug intervention*

3. **Clarify the possibility of combining the first two visits of the study protocol** (*visits 1: Enrolment* and *visit 2: Baseline*) in as many visits (including only one) as each center consider feasible for their local conditions. It will be mandatory, however, to complete all needed evaluations before randomization, including the complete laboratory assessment to confirm inclusion and exclusion criteria **in no more than 1 month before randomization*:***
    - *Section 4.1 Summary of Trial and Section 7: Study Visits.*

4. **Rescue therapy**: A similar criteria previously established for HbA1c, was applied for any glucose assessment for the diagnosis of diabetes during follow-up:
    - *Section 4.1 Summary of Trial. Flow chart, Section 4.2. Randomization and code breaking Section 8.1 Intervention Groups*

5. **Rescue therapy**: The addition of the possibility of a documented laboratory analysis, independent of the pre-defined study protocol visits, as a second result to decide the need of a rescue therapy.
    - *Section 4.2.: Randomization and code breaking, Section 8.1 Intervention Groups.*

6. **Rescue therapy**: To make explicit the possibility that the physician already involved in the follow up of the trial be the regular primary care physician of the participant.
    - *Section 4.1 Summary of Trial (Page 13), Section 8.1 Intervention Groups.*

7. References to the **Operation Manual** for detail description of some standard procedures:
    - *Section 4.2 Summary of Trial, Section 7.0: Study visits..*

8. **Exclusion Criteria**: Multiple changes to clarify, add or define some of the exclusion criteria: *Section 5.2 Exclusion Criteria:*

I. Clarify that history of Gestational Diabetes is **not** an exclusion criterion after excluding Type 2 diabetes during enrolment or baseline visit.
II. Includes the use of ANY anti-diabetic drug within the 3 months prior to enrolment in only one item.
III. To avoid redundancy, eliminate the exclusion criteria of "*Clinical evidence of macro-vascular complications (overt clinical cardiovascular disease) at enrolment, including angina (stable or unstable)*." Only participants with evidence of previous CVD or stroke clinically documented will be excluded. We decided to eliminate any kind of angina because the possibility of over-diagnosis based only in subjective clinical symptoms without any clinical evidence of cardiovascular disease. The possibility of silent previous Myocardial Infarctions based on previous EKG is still valid.
IV. Specify the types of renal replacement therapy.
V. Include primary biliary cirrhosis as exclusion criteria.
VI. The evidence of liver, pancreatic or renal disease based on laboratory parameters is specified as within the previous 6 months. However, these biochemical evaluations MUST be performed as part of the baseline evaluation within no more than 1 month before randomization.
VII. Specify that upper normal ranges for pancreatic and liver enzymes will depend on of the normal reference stated by the local laboratory, which will need to be informed to central coordination.
VIII. Include any prior organ transplant and not only solid organ.
IX. Include morbid obesity and bariatric surgery plan in the next 5 years as exclusion criteria, because the possibility on interference with experimental trial.
X. Clarify that participant with history of cataracts surgery are allowed to enter the trial if the cataracts are resolved.
XI. Include the following criteria because of its interference with an adequate retinal evaluation included in the primary outcome index: Ocular surgery planned in the next 6 months (patients appointed for cataract surgery can be accepted after surgery if the recruitment period is still open), concomitant intraocular treatment (retina or choroid) and Tropicamide allergy(drug used to dilate the pupils).
XII. Includes the need of a screen retinogram during enrolment-baseline evaluation to centrally (WP6 leaders) evaluate if the participant is optimal for retinal assessment of primary outcome.
XIII. Add the criteria of complete amputation of hands or feet because the impediment to measure sudomotor reflex based on SUDOSCAN procedure. This is part of the primary outcome.
XIV. Specify examples of institutionalization.
XV. To avoid redundancy in contraindications of any study drug we only kept those not mentioned in other criteria.

9. **Criteria to be withdrawn from the investigational product**. *Section 5.3 Subject Withdrawal Criteria:*
   I. To be consistent with exclusion criteria at baseline, the definition of the upper limit for moderate renal impairment is based on CrCl <60 mL/min instead of CrCl <50 mL/min.
   II. Add the criteria of acute pancreatitis.

10. **Lifestyle Assessment:**
    - Consistency between table and text: Physical activity and dietary evaluation at 12, 18, 36 and 60 months: *Section 6.1: Lifestyle habits assessment.*

11. **Include the use of Hemocue®** system as the mandatory standard protocol to perform all glucose measurement for all study visits at the point of care.
    - *Section 6.3 Biochemical measurements*, *Section 7.1 Screening and Eligibility Assessment Enrolment and Baseline visits*.

12. **Specify that the techniques used for each biochemical analysis at the local laboratory facility** need to be documented and sent to the coordination entity before the start of the trial for an approval from the WP4 leader.

- *Section 6.3: Biochemical measurements and Section 7.1 Screening and Eligibility Assessment Enrolment and Baseline visits.*

13. **A new section was added to describe all study visits** in an independent *Section 7: Study Visits.*

14. **A new detailed description of the pre-screening, enrolment and baseline visits** is included in *Section 7.1 Screening and Eligibility Assessment Enrolment and Baseline visits*. In addition of a clearer description, the most important changes from the previous version are:
    I. The possibility that potential candidates might be contacted by letter, telephone or both for a first evaluation of inclusion/exclusion criteria. If the participants is likely to fulfil the study´s criteria, she/he can be invited to the enrolment visit (visit 1)
    II. A suggestion to do first the evaluation of the OGTT and the screening retinogram to avoid further testing and interviews in ineligible participants. (Page 28-29).
    III. A description of how to send the photograph of the screening retinogram for central evaluation.
    IV. To avoid an important number of screening failures because the expected intra-person variability in glucose levels and consequently inconsistencies in dysglycemia classification, the use of the average between the baseline full OGTT (5 points) and the previous OGTT used for screening (including OGTT performed within 6 months previous to baseline evaluation) is included.
    V. A note to describe in which cases the glucose assessment (including OGTTs) can be performed using capillary blood.

.

15. **Changes in procedures included in study visits**. Some changes were decided to simplify as much as possible the study protocol to decrease the workload at clinical centers without interfering in the primary and secondary objectives. Other changes were to correct inconsistencies between tables and text in previous versions. The most important changes are:
    I. To define as mandatory the primary outcome evaluation **only** at baseline, 36th month and 60th visits.
        - Section 6.4 Assessment of primary outcomes
    II. For those centers included in a subset for secondary outcomes such as CIMT, Endo-PAT, Sleep analysis (Somnomedics), etc, these assessment will be mandatory to perform at baseline, 36th month and 60th visits.
        i. Section 6.5 Assessment of secondary endpoints.
    III. Optionally and based on local feasibility, each clinical center might decide to include additional evaluations of SUDOSCAN, CIMT and Endo-PAT at 12th and/or 24th months visits.
        - Section 6.4 Assessment of primary outcomes, Section 6.5 Assessment of secondary endpoints.
    IV. To eliminate EKG as a mandatory test in any visits because a prior expectations of minor changes during follow up and the impossibility of having a central reading center for standard interpretation. It will remain as an optional if the center is willing to include it.
    V. To eliminate Ankle–Braquial pressure measurement as mandatory in any visit.

16. **A clarification that the IDF-Europe will participate only in the dissemination strategy** but no in monitoring clinical standards of diabetes care as it was mentioned in the previous version.
    - *Section 6.6 Informed Consent.*

17. **The specific name of the local pharmacy** to prepare the packing and labelling of the drug bottles was eliminates because it is not totally confirmed.
    - *Section 8.3: Drug intervention.*

18. **The use of Medication Event Monitoring System (MEMS®)** by AARDEX® group in a subsample of 20% of all participants was added as an additional objective measurement of drug adherence.

- *Section 8.3: Drug intervention.*

19. **A procedure to send the report of any SAE** is described.
    - Section 10.3 Procedures for Recording Adverse Events.

20. An explicit statement that the determination of the **coefficients to construct the primary outcome index** it won't be susceptible to be manipulated based on the results of this study.
    - Section 11: Sample Size Calculation.

21. Multiple changes to correct grammatical and typographical errors. Across the entire document.

22. **Changes in Informed Consent Letter and Form**: In additional some corrections of grammatical and typographical errors (independent document e-predice_ICF_V3.0_06_05_2013)

- The addition of an statement related to scientific news during the trial that might influence the decision of the participant to continue in the trial
  i. *Section 4: Do I have to participate?*
- Elimination ABI (Ankle braquial Index) as procedure to be performed. If a specific center decides to include it need to be added in local CLF forms previous notification to the coordination entity.
- EKG as an optional.
- Independent description of the two versions of OGTT: Simplified version (only fasting and 2 hours) and complete OGTT (5time points).
  Expand the information about the specific insurance referring the European Directive that regulates it.
  i. *Section 8: hat if something goes wrong?*
- Clarify that by marking the initials in each statement, the participant is accepting each option and it is free to accept each of them independently
  i. *Informed Consent (page9).*
- A new statement clarifying that if after signing this consent form, if during the review of the eligibility criteria the participant won't fulfil the criteria, he/she will not be invited and the consent form is not valid anymore.
  i. *Informed Consent (page9).*

1.2 List of amendments from version 3.2 to version 4.2 (02 January 2014)

1. Name of the Study protocol: Changes in this document to be consistent to the Informed Consent Letter and Form and with Insurance Policy certificate. From "Early Prevention of Diabetes Complications in Europe" to Early Prevention of Diabetes Complications in people with Hyperglycaemia in Europe: Study e-PREDICE"
2. **Drug Intervention**: Because of the MSD voluntary decision to cancel the donation of the study drugs: Januvia (sitagliptin), Janumet (sitagliptin+metformin) and the placebo of Januvia, these drugs have been substituted by similar drugs (DPP-4 inhibitor) of Boehringer Ingelheim (BI): Trajenta (Linagliptin 5 mg) and Jentadueto (Linagliptin 2.5 +Metformin 850), and a placebo for Trajenta. Therefore the new treatments arms are as follows and has been substituted across the document:

INTERVENTION GROUPS	
1	**Lifestyle intervention + one placebo tablet/twice a day**: One during breakfast, one during dinner). Placebo pills with identical aspect of linagliptin 5 mg active drug pill
2	**Lifestyle intervention + *Metformin* 850 mg/ twice a day;** One during breakfast, one

	during dinner). No placebo pills.
3	**Lifestyle intervention + *Linagliptin* 5mg/day:** One tablet of active drug during breakfast and one placebo tablet during dinner. Placebo pills with identical aspect of linagliptin 5 mg active drug pill
4	**Lifestyle intervention + fixed-dose combination of *Linagliptin* 2.5mg with *Metformin* 850 mg/twice a day:** One during breakfast, one during dinner. No placebo pills.

3. **Blind strategy**, still defined as **partially double blinded:** placebo pills will be prepared only by BI with identical shape of the active Linagliptin 5mg mg pill. Two placebo pills will be provided blinded to the treatment arm that receives life-style intervention only (group 1) and one placebo pill will be provided to the treatment arm that receives 5 mg of Linagliptin per day (group 3). For these two treatment arms (group 1 and 3) the trial is totally double blinded. However, for the two other arms only the bottles will be identical (as for all four treatment arms) but the shape and colour of the pills will be different and unique with the possibility of being identified, at least by the physician in charge. Therefore, for these two groups, the blindness can only be considered partially blinded. All four groups will receive 2 pills per day.

NOTE: Any previous amendments related to drug intervention that contradicts the above definition of intervention groups are not longer valid**.**

4. **Additional text supporting the use of Linagliptin** and corresponding references in section 2.1: background information.
5. **Corresponding changes to the Informed consent letter and form** (e-predice_ICF_V3.2_05_07_2013) to incorporate the new drug treatments.
6. Add a **7x24 record of number of step measured by pedometer** as an optional measure of adherence of physical activity intervention.
   Section 6.1: life habit assessment
7. Change the time of the optional assessment with SUDOSCAN from 2 optional assessments in months 12 and 24 months visit t only one optional at mid time of the trial (18 months). This will be more informative and reduce the work lead in clinical sites.
   Section 6.4 Assesment of primary outcome
   Section 7.2:Subsequent Assessments

8. Specify that the responsible of central readings of IMT for quality controland and quality assessment will be performed at the Cardiovascular Imaging Unit ICICORELAB (EVIDEM), Valladolid, Spain (WP5).
   Section 6.5: Assessment of secondary endpoints.

9. Eliminate the HAM-Anxiety Rating Scale (HAM-A) because was redundant with the anxiety assessment with the MINI 600 included modules. HAM-D (for depression) was kept because the special interest on depressive symptoms.
   Section 6.5: Assessment of secondary endpoints.
   Section 7.2:Subsequent Assessments

10. Specify that OGTT can be performed with capillary blood ONLY for the OGTT used for screening purposes. However any other OGTT, including full OGTT (5 time points) at baseline and final visit at month 36th and other simplified versions at months 12 and 24 (see below) venous whole blood is mandatory. This will increase the comparability across centres.
    10.1Section7.1: Screening and Eligibility Assessment

11. Add signature page and executive summary.

1.3 List of amendments from version 4.2 to version 4.3 (02 March 2015)

Change in the study sponsor: FUNDACION DE INVESTIGACION EN RED EN ENFERMEDADES CARDIOVASCULARES (FIRCAVA). Almagro 1, Madrid 28010 Spain
Legal representative**: Francisco Fernández Avilés**
By
EVIDEM CONSULTORES SL (EVIDEM). Calle Marroquina 12 – 1ºA 28030 – Madrid
Legal representative**: Ana Roson Hernandez**

Pages: 1, 2, 7, 26, 29, 58, 63, 64

1.4 List of amendments from version 4.3 to version 5.0 (11/26/2015)

1. Changing the time tracking from 3 to 2 years
2. Visits 9 and 10 are removed
3. The sample of participants decreases from 3000 to 2,000
4. Visits updated plan is attached

1.5 List of amendments from version 5.0 to version 5.1 (12 June 2018)

1. Change in the number and timeline of clinical follow-up visits:

1.1.- Visit 6 (month 18) moved to month 24 (old visit 7)

1.2.- Visit 7 (month 24) moved to month 36 (old visit 8)

1.3.- Cancelation of month-18 visit

2. Change of drug delivery after month 24:

2.1.- All 4 drug groups will be delivered as open-label from month 24 to month 36 of follow-up. Obviously, Placebo treatment is not longer any more after month 24.

3. Change in retinal protocol:

3.1.- At month 24 or month 36 ONLY three retinal photos per eye (instead of 7 per eye) will be taken. Please see specific ophtalmological operation manual for details.

4. Cancelation of questionnaires in visit month-6, month-12, month-24 and month-36:

4.1.- 3x24 Hour Physical Activity diary

4.2.- MINI 600

5. Cancelation of MEMS assessment

6. Adjustment of sample size and statistical power

ABBREVIATIONS

AE	*Adverse event*
AR	*Adverse reaction*
CrCl	*Creatinine Clearence*
CRF	*Case Report Form*
CT	*Clinical Trials*
DPS	*Diabetes Prevention Study*
EC	*Ethics Committee*
EMA	*European Medical Agency*
FPFV	*First Patient First Visit*
GCP	*Good Clinical Practice*
GP	*General Practitioner*
OP	*Operation manual, Investigators Brochure*
ICF	*Informed Consent Form*
ICH	*International Conference of Harmonisation*
IFG	*Impaired Fasting Glucose*
IGT	*Impaired Glucose Tolerance*
IRB/IEC	*Independent Review Board/Independent Ethics Committee*
FPG	*Fasting Plasma Glucose*
T2D	*Type 2 Diabetes*
PI	*Principal Investigator*
SAE	*Serious Adverse Event*
SAR	*Serious Adverse Reaction*
SMPC	*Summary of Medicinal Product Characteristics*
SUSAR	*Suspected Unexpected Serious Adverse Reactions*
WHO	*World Health Organization*

2. INTRODUCTION

2.1 Background Information

Definition of diabetes is arbitrary. Although blood glucose distribution is a continuum in the population, Type 2 Diabetes (T2D) is defined by the WHO as a condition characterised by hyperglycaemia and the risk of long term development of microvascular and macrovascular complications. The current fasting plasma glucose (FPG) parameters used to diagnose diabetes derive largely from diabetes specific microvascular complication data, especially retinopathy (1).

The current diagnostic criteria distinguish a group with significantly increased prevalence of microvascular complications, i.e. a significant proportion of people diagnosed with diabetes using current criteria already have specific complications due to hyperglycaemia at the time of diagnosis.

Although much more attention has focused on the diabetic macrovascular disease (stroke and acute coronary syndromes), the morbidity associated with diabetic microvasculature disease, including retinopathy, neuropathy, nephropathy, and limb ischemia, is staggering. Pathological changes in the diabetic microvasculature alter organ perfusion, including organs heavily dependent on their microvasculature supply, namely the retina, kidneys, and peripheral nervous system. The clinical problems associated with these changes–retinopathy, nephropathy, and neuropathy–drive a large burden of T2D morbidity (2).

A linear relationship exists between hyperglycaemia and microvascular complications. The DECODE study has shown a linear association between glycaemia and the risk of complications, even in the normoglycaemic range. Since high blood glucose reaches all tissues, every cell in the body will become more or less damaged. For instance, impaired glucose tolerance (IGT), is associated with a 40% of increased mortality and the risk of both microvascular and macrovascular complications (3). T2D leads to both macro- and microvascular complications that are responsible for most of the associated excess morbidity and mortality. While macrovascular complications such as cardiovascular disease are the most frequent cause of excess mortality, microvascular complications such as retinopathy, nephropathy and neuropathy are responsible for much of the excess morbidity.

A significant proportion of dysglycaemic individuals in population-based studies, show already typical retinal lesions characteristic of diabetes at baseline (12% of IGT participants in the DPP study), and are more likely to develop complications than normoglycaemic people (4). Cross-sectional data from the Blue Mountains Eye Study (BME), the Australian Diabetes, Obesity and Lifestyle Study, and the Multi-Ethnic Study of Atherosclerosis showed no uniform glycaemia threshold for retinopathy across different populations. These data suggest that microvascular complications do not occur at an arbitrary glycaemia threshold, a notion also raised for macrovascular disease (5). The BME study examined the relation between baseline FPG and “any” incident diabetic retinopathy (DR) showing that FPG poorly predicted incident diabetic retinopathy after 5 years, with most incident retinopathy cases occurring in people with FPG below the 7.0 mmol/L cut-off.

The interaction between glucose levels and microvascular disease requires a molecular explanation. Several targets cells can be implicated in diabetic microvascular disease pathways as the endothelium, the podocytes, the pericytes and the vascular smooth muscle cells (VSMCs). Capillary endothelial cells in the retina, mesangial cells in the renal glomerulus, and neurons and Schwann cells in peripheral nerves can all be categorized in this

way (6, 7). In summary, hyperglycaemia is a driving force in both large and small vessel disease. Indeed, microangiopathy and macroangiopathy may be interconnected, with microvascular disease promoting atherosclerosis.

Once established, T2D is difficult to treat and normoglycaemia unlikely to be achieved. Despite of pharmacologic treatment, blood glucose levels tend to increase over time. Fortunately, several studies have convincingly demonstrated that prevention of T2D is possible with lifestyle modifications. The Finnish landmark Diabetes Prevention Study (DPS) proved that lifestyle modifications can significantly reduce the onset of T2D by 58% in high risk subjects (8), later confirmed by the DPP study and many others (9). Unfortunately half of people with hyperglycaemia failed to achieve normoglycaemia with lifestyle intervention alone, and 1/3 progressed to T2D in 10 years in the Diabetes Prevention Study (DPS) (8).

Major trials on diabetes prevention have limited data on diabetes complications, and despite the perceived value of lifestyle interventions, whether the reduced incidence of diabetes also translates to a reduced incidence of microvascular complications remains uncertain (11). Recently the Chinese Da Qing Trial (10), a randomized controlled CT of combined lifestyle interventions (diet + physical activity) for 6 years in IGT people, reported a 47% reduction in the incidence of severe DR after adjusting for clinic and age, compared to the control group (hazard rate ratio 0.53, 95% CI 0.29–0.99, p=0.048).

There is a 30-year cumulative evidence that effective metabolic control during the earlier years of the disease significantly reduces long-term macrovascular complications (11,12). Several trials have attempted to demonstrate whether intensive glycaemic control with blood glucose lowering drugs can prevent microvascular complications, mainly the progression of diabetic retinopathy, in T2D patients with advance disease and high risk for cardiovascular disease. Although these trials shown that intensive glycaemic control reduced the rate of progression of diabetic retinopathy, the microvascular benefits of intensive therapy should be weighed against the increase in total and cardiovascular disease-related mortality, increased weight gain, and high risk for severe hypoglycaemia (13). No controlled trial to date has attempted to determine the lowest level of glycaemia where glucose-lowering therapy may have beneficial effect on the incidence of diabetic complications. Moreover, it is not known at all to what extent the currently proposed optimal targets for glycaemic parameters can be achieved in patients at early stage of the disease. In comparison, such trials have been carried out for blood pressure and serum total (LDL) cholesterol a long time ago, resulting in lowering the cut points to initiate active therapy. The NAVIGATOR trial, published recently, attempted and failed to prevent diabetes complications with single pharmacotherapy in IGT people. Therefore, new well designed trials with novel therapeutic approaches are needed; in particular how pharmacologic and lifestyle interventions can be combined and applied “in the best manner” and “as early as possible” to prevent diabetes complications (14).

Pharmacological treatment for hyperglycaemia at early stages:

The ADA/EASD algorithm for the management of T2D (January 2009) critically review the clinical advantages and disadvantages of different glucose-lowering therapies currently available. Metformin is the only blood glucose lowering drug recommended by the ADA in people with IGT who do not respond to lifestyle interventions, but this recommendation is based on expert opinion only, and there are no clinical trials showing metformin preventing diabetes complications in IGT people (15, 16).

An alternate therapeutic algorithm to the ADA/EASD recommendations is based upon known pathophysiological disturbances. This algorithm provides a more rational approach and is more likely to produce a durable long-term

effect. This algorithm initiates treatment with lifestyle modification plus combination therapy with drugs known to improve insulin sensitivity and, most importantly, with drugs that have been shown to preserve β-cell function (17). Neither the sulfonylureas nor metformin alone has been shown to preserve β-cell function..

New incretin-based therapies (DPPIV inhibitors and GLP-1 agonists) have been approved for use in Europe and other regions of the world with evidence of efficacy when used alone or in combination with metformin in T2D patients (18). A recent meta-analysis evaluated the efficacy and safety of dipeptidyl peptidase-4 (DPP-4) inhibitors and metformin as initial combination therapy and as mono-therapy in patients with T2D. DPP-4 inhibitors had a stronger effect than metformin as a mono-therapy on lowering HbA1c and other cardiovascular risk factors suggesting a potential effect on reducing the risk of CV events in patients with T2DM. (19). Several large trials with different DPP-4 inhibitors are currently under way in patients with T2D and high-risk cardiovascular profile (TECOS with Sitagliptin, SAVOR-TIMI with Saxagliptin, EXAMINE with Alogliptin and CAROLINA with Linagliptin) that may confirm the long-term conclusions and CV events protection (20-22),

In addition DPP4 inhibitors are safer than the classical glucose lowering drugs for hypoglycaemia because their action is mainly on postprandial glycaemia, with preservation of the β-cell function. In contrast, the remaining β-cell function in the hyperglycaemic states will largely be lost with sulfonylureas or metformin. (23). A recent publication of a pooled analysis of 19 clinical studies including 10,246 patients with type 2 diabetes, about the safety and tolerability of the usual monotherapy clinical dose of sitagliptin, the first approved DPP4 (showed that treatment with sitagliptin was not associated with an increased risk of major adverse cardiovascular events in treatment between 12 weeks and 2 years (24)

To our knowledge, there are no published studies comparing metformin and DPPIV inhibitors with lifestyle modification in non-diabetic people with hyperglycaemia.

2.2 Rationale or Justification

ePredice is an European project funded by the 7th Framework Program. It is a long-term, multi-centre, randomised, partially double blinded controlled (four parallel groups), phase-IIIb clinical trial, with prospective blind outcome evaluation. As a primary objective, this project seeks to evaluate the effects of treatment with, linagliptin, metformin, or their combination plus lifestyle intervention (diet and physical activity), compared to lifestyle intervention alone, for at least 2 years, and up to 4 years, on different micro-vascular parameters (retinal, renal and neurological), in adults with hyperglycaemia (Impaired Fasting Glycaemia (IFG), Impaired Glucose Tolerance (GT), or both), but without Diabetes.

Besides the main primary outcome to evaluate the effect of the different treatments regimes on the early prevention of diabetic micro-vascular complications, this project will include the evaluation of a broad spectrum of early markers of vascular function and structure in a subset of participants. These secondary endpoints include subclinical atherosclerosis, endothelial function and serological biomarkers of systemic inflammation, early micro-vascular damage, non-alcoholic fatty liver disease (NAFLD), insulin sensitivity and insulin secretion, among others. This multiple endpoints assessments will be extremely valuable to evaluate the mechanisms by which these drugs and lifestyle intervention might be acting to reduce the micro- and macro-vascular complications.

3. OBJECTIVES AND PURPOSE

General Objective:

To assess the long-term effects of different therapeutic regimens, both pharmacological and lifestyle interventions, for the early prevention of diabetic complications in people with non-diabetes intermediate hyperglycaemia (IFG, IGT or IFG plus IGT).

3.1 Primary objective:

1- To assess the effect of treatment with linagliptin, metformin or the combination of linagliptin with metformin, plus lifestyle intervention (diet and physical activity), compared to lifestyle intervention alone, for at least 2 years, and up to 4 years, on different microvascular parameters (retinal, renal and neurological), as defined by the primary and secondary endpoints, in adults with non diabetic hyperglycaemia (IGT, IFG or IFG plus IGT).

3.2 Secondary objectives:

1- To identify among people with hyperglycaemia who are most likely to develop early diabetic complication
2- To find out which of the early diabetic complications can be best prevented by interventions applied in this study
3- To determine the extent to which the compliance to the interventions affect the rate on early diabetic complications prevention
4- To evaluate the effect of the different treatment regimens applied in this study on quality of life and neuropsychological functions
5- To assess the efficacy of treatment with linagliptin, metformin and the combination of linagliptin with metformin plus lifestyle intervention in comparison to lifestyle intervention alone with regard to surrogate parameters of vascular function and structure, and novel biomarkers of microvascular damage in adults with non-diabetic hyperglycaemia (IGT, IFG or IFG plus IGT)
6- To determine the safety of linagliptin, metformin and of the combination of linagliptin with metformin plus lifestyle intervention in people with non diabetic hyperglycaemia with regard to severe adverse effects and clinically important events

4. TRIAL DESIGN

4.1 Summary of Trial

Investigator initiated (non-commercial) Long-term, multi-centre, randomised, partially double blinded, controlled (four parallel groups), phase-IIIb, clinical trial with prospective blind outcome evaluation.

The design is partially double blinded in terms that it will be double blinded only between one of the active drug arms (linagliptin only) and the placebo arm and single blinded (only for the patient) for the arm with metformin alone and the fixed combination of metformin/linagliptin. Complete description in section 8: Interventions.

The study will have 3 main periods:

1. **Screening procedures and Recruitment period**: 12 months
2. **Intervention period:**
   - ***First phase***: A three-month run-in period to evaluate drug tolerability.
   - ***Second phase***: Parallel treatment and clinical follow-up during 21 additional months (see flowchart of Visit Plan).
3. **Extended follow-up:** Epidemiological observational follow up and outcome evaluation after unmasking experimental treatment: 12 additional months (from month 24th to month 36th).

1- Screening procedures of eligible participants and patient recruitment:

Screening will be performed in the first place using any opportunistic screening strategy, either in the community, occupational or primary care settings, already established in the clinical centre. The validated European FINDRISC questionnaire (31) can be used but will not be mandatory, to estimate the individual risk of developing T2D and decide who will be candidate for an OGTT to define eligibility criteria for the trial.

If the centre already has a pre-established and ongoing strategy for screening people to early diagnosis of diabetes or pre-diabetes, databases and clinical records can be reviewed at each recruiting centre before the enrolment visit in order to identify potential eligible subjects for the trial.

Enrolment **(visit 1 in visit plan (appendix A of protocol V5.0))** will take place within two weeks and no more than one month before initiation of baseline visit (visit 2) and randomisation. Subjects must give signed and dated informed consent prior to any trial-related activities. Subjects must be provided with a copy of the subject information and a copy of their own signed and dated informed consent form. Subjects will be assigned a unique number (lowest available number allocated to the site) which will remain the same throughout the trial.

During the enrolment or baseline visit **(visit 1 or 2 in visit plan (appendix A of protocol V5.0))**, the following information will be collected and recorded**:** informed consent signed and dated; confirmation of inclusion and exclusion criteria; demographics: date of birth, race and ethnicity; clinical variables: concomitant illness/medical history (including diabetes history and complications, history of concomitant cardiovascular disease, history of pancreatitis and gallstone disease), FINDRISC scoring, concomitant medication, physical examination (systolic, diastolic blood pressure and pulse rate, height, weight and waist circumference) and smoking habit. A standard OGTT with 75 gr. of oral glucose solution will be needed for the determination of plasma glucose at fasting and 2 hrs post load to indentify participant with no diabetic hyperglycaemia (IGT, IFG or both) eligible for the trial. .

In addition, in the enrolment visit fasting blood sample, HbA_{1c}, basic hepatic and pancreatic parameters (ALT, gamma GT, bilirubin, amylase and lipase) and serum lipid profile (total-cholesterol, HDL-cholesterol and triglycerides) will be measured. In females at childbearing age, not on contraceptive management, a pregnancy test will be also performed.

All data related to the enrolment visit (visit 1) and baseline visit (visit 2) must be reviewed by the investigator to ensure that the subject is eligible to continue in the trial. If the person is NOT eligible, the subject MUST be registered as a “screening failure”. The reason for failure should be also recorded (see below).

2- Intervention: after randomization patients will be assigned to any of the 4 following intervention groups during 24 months: (see flowchart):

- **Group 1: Lifestyle intervention + two placebo tablets a day (one during breakfast, one during dinner)**. Placebo pills will have the same shape of linagliptin 5mg active drug tablet.
- **Group 2: Lifestyle intervention + two *Metformin* tablet 850 mg per day (one during breakfast, one during dinner).** No placebo pills.
- **Group 3: Lifestyle intervention + 1 tablet of *Linagliptin* 5 mg during breakfast and one placebo pill during diner:** Placebo pill will have the same shape of linagliptin active drug .
- **Group 4**: **Lifestyle intervention + 2 tablets of a fixed-dose combination of *Linagliptin* 2.5 with *Metformin* 850mg (one during breakfast, one during dinner).** No placebo pills

Rescue therapy:

If in any of the 4 groups, if HbA1c >6.5% or fasting glucose ≥7.0 mmol/l or 2 hr post load glucose ≥11.1 mmol/l is observed in two consecutive study visits (including a documented laboratory results independent the pre-established study protocol visits), the patient will be refer to his/her primary care physician to confirm diabetes diagnosis and if needed individualized therapy will be added. According to the data from the Finnish Diabetes Prevention Study the mean HbA1c did not increase in the lifestyle intervention group at all during the first 4 years (32). Thus, very few individuals are expected to cross this threshold in ePredice. The blinded experimental therapy will be open (unmasked) in order to provide all relevant information to the primary care physician to decide the individualized rescue therapy. A letter to independent primary care physician will be provided with an explanation of the trial, encouraging to favour the maintenance of the experimental treatment (that will be still provided by the trial) and add any hypoglycaemic treatment that will not interfere with the experimental therapy, avoiding the use of metformin and DPP4 inhibitors. Patient´s clinical follow-up (and outcome evaluation) will be kept unchanged and the added therapy will be recorded and considered in the finally analysis. If the regular primary care physician is already the physician in charge of the follow up of the trial or the participant does not have a regular primary care physician, the individualized therapy can be added by the trial´s physician.

If pharmacological treatment for other CVD risk factors (blood pressure or lipid lowering drugs) becomes indicated over the course of the trial, therapy can be prescribed at the discretion of the responsible physician.

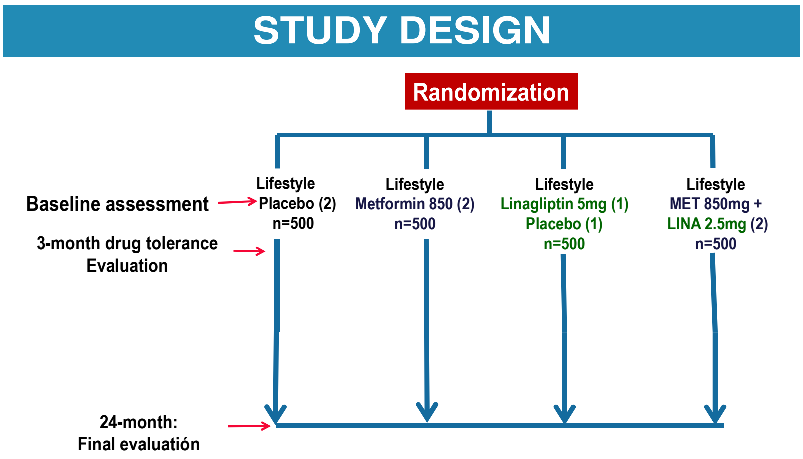

Lifestyle intervention

All patients will be enrolled in an intensive lifestyle intervention program to change several lifestyle habits simultaneously and become healthy (group 1 only in intensive lifestyle intervention, and groups 2, 3 and 4 in intensive lifestyle intervention plus the corresponding random allocated drug). The lifestyle intervention will be provided by staff nurses, physical activity technicians and/or nutritionists previously trained by experts. The intervention contains informative (diet, physical activity) as well as behavioural and motivational modules and consists of 2 (+ optional 1) individual counselling sessions including personal goal setting, and small-group (up to 10 participants) sessions, repeated every month during the first 6 months and thereafter every 3 months. Each session would last for one hour for individual sessions and 2 hours for group sessions. Optionally, based on recruitment centre resources, physical activity intervention can be delivered by group coaching (two sessions per week starting from month 3) and will facilitate access to local gyms and sport clubs; otherwise physical activity will be strongly emphasized during the group sessions. The lifestyle programme will be adapted to the local circumstances but keeping the same goals.

The lifestyle goals will be based on DPS (8) which extensively proved their efficacy to prevent T2D:

- weight loss (>5% of baseline weight or BMI<25)
- moderate total fat intake (30-35 E %)
- reduced saturated and trans fat intake (<10 E %)
- high fiber intake (>15 g/1000 kcal)
- exercise >4h/week
- smoking cessation.

Consumption of vegetables, legumes, fruits and berries, whole-grain cereal, vegetable sources of fat (nuts e.g.), olive or rapeseed oil, and fish will be emphasized. The dietary intervention will be culturally adjusted to local conditions and food supply ("Mediterranean diet", "Baltic sea diet", etc.).

3- Extended follow-up: After 24 months of experimental therapy, the trial will continue as open-label from month 24 to month 36 of follow-up. Observational follow up will be scheduled at month 36th for a telephonic contact to update contact information and invite to remind final appointment at month 36th of recruitment (12 month after the end of the experimental trial) for a complete assessment of primary and secondary outcomes (see appendix A) evaluation. As other visits during follow up, people with diabetes will be referred to their local physicians for treatment. This visit will be cover with global and local funds to be identified later on.

4.2. Randomization and code breaking

Randomization procedure:

Randomization of participants will be stratified by centre with permuted block within each centre to ensure balance between treatment overtime and within centres. Patients will be equally allocated to any of the 4 different groups (p=0.25). Given the partially blinded design, we will randomly vary block sizes to avoid predictability of treatment assignment and selection bias. A web-based, central, independent, randomization will be performed

using the monitoring software implemented specifically for the project using OpenClinica® and block will be allocated to each centre as needed (dynamic allocation of blocks).

Patients will be included in the system during the screening process. Once the patient has fulfilled all the inclusion criteria and if approved for randomization, the system will generate the documentation for the clinical center to start the patient in the trial. Any dropout or withdrawn participant at any time after randomization will NOT be replaced by new participant.

Recruitment-enrolment:

Recruitment will be semi-competitive for all centres. All centres are initially allocated 200 patients for recruitment and an approved recruitment schedule. After three months from First Patient First Visit (FPFV), if a centre falls behind his recruitment schedule (accumulated) by more than 10%, the executive board will redistribute those slots to centres that are recruiting more successfully following a pre-established guideline.

Code breaking:

Drug identification information is to be unmasked only if necessary for the welfare of the subject. There will be five main reasons for code breaking:

1. **At the end of the experimental trial.** At month 24th of initiation of the trial, the experimental treatment will be opened (unblinded). Participant will be followed observationally up to month 48th.
2. **The need of rescue therapy:** As described previously, if anytime during follow up HbA1c >6.5% or fasting glucose ≥7.0 mmol/l or 2 hr post load glucose ≥11.1 mmol/l is observed in two consecutive study visits. Outcome measurements will be carried out as originally planned**.**
3. **The presence** of any serious adverse event (SAEs). Outcome measurements will be carried out as originally planned.
4. **By voluntary withdrawn** of participant form the trial, but if possible, outcome measurements will be carried out as originally planned.
5. **Pregnancy** of a female participant during the trial: lifestyle intervention and metformin will continue as open therapy, and outcome measurements will be carried out as originally planned.

Any other premature code breaking (e.g. accidental unblinding) should be communicated to the coordinating office following a pre-established standard procedure (see Operation Manual).

4.3. Primary and Secondary Endpoints/Outcome

Microvascular complications primary endpoints

The primary endpoint is a combined continuous variable: ***"the microvascular complication index" (MCI***) composed by the linear combination of the Early Treatment Diabetic Retinopathy Study Scale (ETDRS) score, the level of urinary albumin to creatinine ratio, and sudomotor test (SUDOSCAN) score, measured during the 24th and 36th month visits.

The rationale for the use of this endpoint is given in the statistical analysis plan (SAP) (Section 8).

Secondary endpoints:

Each of the three single components of the microvascular composite index (MCI) will be also evaluated as continues changes between baseline and visits with complete assessment at months 24th and 36th, and as total number of patients developing at least one of the following conditions:

- Retinopathy score at last visit defined as 2 steps progression on the ETDRS scale between baseline and visits at month 24th and/or 36th.
- One standard deviation (SD) increase on the level of urinary albumin to creatinine ratio between baseline and visits at month 24th and/or 36th
- One SD decrease Changes in the level of hands and feet conductance in SUDOSCAN between baseline and visits at months 24th and/or 36th.

Other secondary endpoints to be used as supportive endpoints for the primary objective are:

- Change in microvascular endothelial function (MEF) measured by the Endo-PAT method (in a subset).
- Change in the Non-Alcoholic Fatty Liver (NAFL) Index (in a subset).
- Change in biomarkers of microvascular damage, endothelial function, per-oxidation, inflammation, and metabolomics (in a subset).
- Change in the insulin secretion and β-cell function (in a subset)
- Change in self-perceived Quality of Life (QoL).
- Change in symptoms of peripheral neuropathy
- Change in neuropsychological parameters: cognitive function, and depressive symptoms and indices.
- Changes in Obstructive Sleep Apnoea (OSA) indices as measured by Somnomedics.(in a subset)
- Changes in ambulatory blood pressure monitoring as measured by Somnomedics (in a subset)
- Change in the mean common carotid intimae-media thickness (CIMT) (in a subset).
- Incidence of major cardiovascular events, defined as an expanded composite of total coronary events, total stroke events, revascularization procedures (coronary artery bypass graft, percutaneous coronary angioplasty, and peripheral revascularization), hospitalization for heart failure, transient ischemic attack, and cardiovascular or cerebrovascular death

Safety endpoints (definition in section 9)

- Incidence of any adverse events (AE) including hypoglycaemic episodes
- Incidence of serious adverse events (SAE).
- Incidence of any adverse events leading to treatment discontinuation.
- Incidence of medical events of special interest: Neoplasm, Pancreatitis or acute, severe and persistent abdominal pain leading to a suspicion of pancreatitis, Acute gallstone disease (biliary colic or acute cholecystitis),

A set of centres will be chosen to be included within the subsample for the evaluation of secondary endpoints. This selection will depend on the willingness and feasibility of the centre and the pertinence evaluation from WP5 leaders. The centres will be selected well before the recruitment phase and communicate to each selected centre.

5. STUDY POPULATION

Males and females with IFG, IGT or both conditions, aged 45 to 74 years, selected from the following clinical centres (see table 1)

Table 1 Clinical centres

PARTICIPANT ORGANISATION NAME	COUNTRY
GEMEINNÜTZIGE SALZBURGER LANDESKLINIKEN BETRIEBSGESMBH (SALZBURGER LANDESKLINIKEN, SALK)	AUSTRIA
SCIENTIFIC ASSOCIATION FOR THE SUPPORT OF THE FDPM EDUCATIONAL & RESEARCH ACTIVITIES (SAFEA),.(2 CLINICAL CENTRES)	GREECE
ISTANBUL UNIVERSITY	TURKEY
MEDICAL SYSTEM BEOGRAD (MSB)	SERBIA
FACULTY OF MEDICINE, UNIVERSITY OF BELGRADE	SERBIA
UNIWERSYTET JAGIELLONSKI	POLAND
UNIVERSITY MULTI-PROFILE HOSPITAL FOR ACTIVE TREATMENT ALEXANDROVSKA EAD	BULGARIA
ASOCIACIÓN INSTITUTO MALAGUEÑO DE INVESTIGACIÓN CLÍNICA	SPAIN
EVIDEM CONSULTORES	SPAIN
DASMAN DIABETES INSTITUTE	KUWAIT

5.1 Inclusion Criteria

- Men and women
- Age 45 -74 years
- Impaired Fasting Glucose (IFG): FPG 6.1 to 6.9mmol/l and 2-h PG <7.8mmol/L; or Impaired Glucose Tolerance (IGT): FPG <7.0mmol/L and 2h PG >_7.8 and <11.1 mmol/L; or both conditions;
- Informed consent given

5.2 Exclusion Criteria

At Baseline evaluation:

- Type 1 diabetes.
- Known* or screen detected T2D with or without pharmacological treatment. History of Gestational Diabetes is not an exclusion criterion after excluding Type 2 diabetes during enrolment or baseline visit.
- Use of any anti-diabetic drug (GLP-1 receptor agonist, pramlintide, dipeptidyl peptidase-4 (DPP-4) inhibitor, metformin insulin or long-acting insulin analogue) within the 3 months prior to enrolment.
- Any previous cardiovascular or stroke clinically documented event or revascularization procedure (stent, angioplasty, by-pass) of any arterial territory documented *.
- Evidence of myocardial infarction in previous EKG.
- Current renal replacement therapy (peritoneal dialysis, haemodialysis or kidney transplantation).
- Previous diagnosis of liver or primary biliary cirrhosis or chronic hepatitis*, or an elevation of liver enzymes (AST and or AST) >3 times of the upper normal ranges** within the previous 6 months or in baseline evaluation. Liver enzymes to be measured during baseline evaluation.

- Previous diagnosis of acute or chronic pancreatitis or an elevation of pancreatic enzymes (Amylase or Lipase) >3 times of the upper normal ranges** within the previous 6 months or in baseline evaluation. Pancreatic enzymes to be measured during baseline evaluation.
- Previous diagnosis of Chronic Heart Failure (NYHA class III or higher).
- A prior organ transplant or waiting for organ transplant.
- Diagnosis of malignant neoplasm requiring chemotherapy, surgery, radiation or palliative therapy in the previous 5 years. Subjects with intraepithelial squamous cell carcinoma of the skin (Bowen's disease) treated with topical 5-fluorouracil (5FU) and subjects with basal cell skin cancer are allowed to enter the trial.
- Known or suspected hypersensitivity to trial products or related products.
- Known use of non prescribed narcotics or illicit drugs.
- Simultaneous participation in any other clinical trial of an investigational agent.
- Females of childbearing potential who are pregnant (all fertile women will be tested for before randomization), breast-feeding or intend to become pregnant.
- Morbid Obesity: (BMI >45 Kg/m2).
- Bariatric surgery plan in the next 5 years.
- Presence of cataract that impedes the retinal evaluation of both eyes. Participant with history of cataracts surgery are allowed to enter the trial if the cataracts are resolved.
- Ocular surgery planned in the next 6 months* (patients appointed for cataract surgery can be accepted after surgery if the recruitment period is still open)
- Concomitant intraocular treatment (retina or choroid).
- Tropicamide allergy (drug used to dilate the pupils)
- Participants with screen retinogram not optimal for retinal assessment based on central (WP6) evaluation
- Complete amputation of one/ both hands or one/both feet. (finger amputations are accepted)
- Dementia, mental disorder or evident cognitive impairment unable to give informed consent.
- End-stage or metastatic cancer.
- Institutionalization (nursing/mental health home, hospital, prison, etc).
- Suspected renal artery stenosis, recent gastrointestinal bleeding (within the last year), and any circumstance where ongoing medication might lead to potential adverse drug interaction with components of the study medications.
- Renal function impairment: GFR $\leq$60 ml/min/1.73m^2. To be measured during enrolment or baseline evaluation.
- Any other acute condition or exacerbation of chronic condition that in the Investigator's opinion would interfere with the trial initiation or visit schedules or procedures. Any medical condition, ongoing medication or significant disability that would prevent the participant complying with informed consent, treatment and follow-up procedures or potentially jeopardize her/his medical care.

Notes:
* Previous diagnosis should be documented by medical record review.
**upper normal limit will depend of the normal reference stated by the local laboratory, which will need to be informed to central coordination.

5.3 Subject Withdrawal Criteria: (terminating investigational product)

The subject may withdraw when he/she wish at any time.

A subject must be withdrawn from the investigational product if the following applies:

- Withdrawal of informed consent
- Pregnancy or intention of becoming pregnant during the treatment period
- Serious adverse events anytime during follow-up to any of the three drug regimens
- Participants with moderate to severe renal impairment documented in any time during follow up (CrCl <60 mL/min)
- Participants with hepatic impairment as documented by an elevation of liver enzymes (ALT or AST) more than 3 times the normal upper value in any follow up visit.
- Participants with acute pancreatitis as documented by an elevation of pancreatic enzymes (Amylase or Lipase) more than 3 times the normal upper value in any follow up visit
- Participants who developed any serious disease that would prevent the measurement of primary endpoints (cancer, CVD, stroke)

Those participants withdrawn in anytime after randomization will not be replaced. If possible, with the exception of voluntary withdrawn from the trial, all other participants withdrawn from the drug will keep the same programmed follow-up until the end of the trial without drug treatment.

The only criterion to stop follow-up is if the participant voluntarily retired from the project

6. STUDY PROCEDURES

6.1 Lifestyle habits assessment

Physical activity and dietary habits will be assessed by a standardized questionnaire. The Physical activity questionnaire, EPIC-Norfolk Physical Activity Questionnaire (RPAQ) and dietary assessment have been successfully used elsewhere and it has been validated previously. Physical activity measured with the questionnaire RPAQ shows a high correlation with physical fitness, measured by maximal oxygen uptake (33). Dietary habits will be assessed by an adaptation of the Mediterranean Diet Adherence questionnaire (MEDAS) (34) composed of 21 categorized or semi-quantitative questions about habitual dietary intake (e.g. dietary pattern, quality and quantity of dietary fat, consumption of fruit and vegetables, grain, milk, and meat products, desserts, sweets and alcoholic beverages) that enables calculation of Mediterranean diet score and estimates for saturated fat and fibre intake. In addition, 6 questions are related to perceived needs and intentions to make dietary changes. Scientific validation of the diet questionnaire is ongoing in the National Institute for Health and Welfare in Helsinki, Finland. This dietary and Physical activity evaluation will be assessed at baseline, 12 months, 24 months and 36 months visits.

In addition, adherence to the life-style intervention will be assessed in every visit after randomization using a 3x24hr Physical Activity Diary and dietary short questionnaire (see Attachment A: Visit Plan), and optionally, by a one week registry of total number of steps measured by a pedometer.

6.2 Non-invasive measurements

Body weight (0.1 kg precision) in light indoor clothing without shoes will be recorded at every visit with a calibrated scale. Heart rate (measured during one minute) and blood pressure (2 mmHg precision) will be recorded twice with a mercury sphygmomanometer on the study participants who are in both seated and standing position. Height (0.5 cm precision) will be measured every year. Waist and hip circumferences will be measured with a measuring tape (0.1 cm precision) and recorded only at baseline and final evaluation for hip and in every visit for waist circumference.

6.3 Biochemical measurements

The oral glucose tolerance test (OGTT) will be carried out according to the WHO recommendations. A 300 ml test solution contains 75g anhydrous glucose and 1.6 g citric acid. The test will start after 12 hours fasting. Fasting, 30-min, 60-min, 90-min and the 2-hour blood sample will be obtained after the ingestion of the solution and samples will be drawn into fluoridated tubes and centrifuged within 30 minutes. At the local laboratory facility, plasma glucose will be determined in all samples times with the HEMOCUE® system. The samples from all times within OGTT will be shipped to a central lab for insulin and C-peptide measurements. Full OGTT (with 5 times measurements) will be performed at baseline, 24 moths and 36m months' visits. OGTT with only fasting glucose and 2hr post load will be performed at month 12 (and optionally at the screening pre-enrolment) with measurements of glucose only at local laboratory facility. HbA1c will be measured at each visit, at the local laboratory facility.

Fasting serum total cholesterol, low density lipoprotein (LDL), high-density lipoprotein (HDL) and triglycerides, concentrations will be determined using an enzymatic method at baseline, 12, 24, and 36 months visits at the local laboratory facility. Other biochemical analysis to be performed at local laboratory facility at baseline, 12, 24

and 36 months visits are: complete hemogram, hepatic profile (AST, ALT, GGT, and ALP), pancreatic profile (Amylase and Lipase), serum creatinine, uric acid and urine analysis for creatinine and albumin concentrations. ***The techniques used for each biochemical analysis at the local laboratory facility need to be documented and send to the coordination entity before the start the trial for an approval from the WP4 leaders.***

6.4 Assessment of primary outcomes

- ***Diabetic retinopathy (DR):*** Grade of DR according to the final Early Treatment of Diabetic Retinopathy Study (ETDRS) grading scale using digital fundus photography. The standardized photography protocol developed by the Joslin Vision Network and validated with ETDRS protocol, will be performed with a non-mydriatic camera under mydriasis with *tropicamide*, and will be carried out by photographers who will receive central training and certification. The photographs will be sent to a central fundus photography reading centre at the University of Valladolid, Spain, where they will be graded by trained readers masked to participant identification and treatment group assignment. Grading will be performed using the modified Airlie House criteria (25). Full retinogram reading and grading will be performed mandatorily at Baseline, 24 and /or 36 months visits.

- **Detection of distal small fiber polyneuropathy** by the measurement of sweat function, using Sudoscan a quick, non invasive and quantitative method. It is based on an electrochemical reaction between sweat chlorides and stainless-steel electrodes in contact with the palm of the hands and soles of the feet. Results are provided as conductances (microsiemens, μS) for the hands and feet (right and left side) and a score of Diabetic Autonomic Neuropathy (DAN) based on conductance values. SUDOSCAN can detect distal small fiber polyneuropathy with a sensitivity of >75%. SUDOSCAN may be considered as a robust method for the detection of sudomotor dysfunction and is used for clinical and research purposes (26-28). Early diabetic peripheral neuropathy (DAN) defined as a reduction of >1S.D. in the conductance values between two measurements SUDOSCAN central reading and interpretation will be done by IMPETO. Full evaluation with SUDOSCAN will be performed mandatorily at Baseline, 24 and 36 months visits. Optionally, centres might decide to include additional evaluations with SUDOSCAN at 12 months visit.

- **Microalbuminuria**: Urine albumin and creatinine levels will be measured in a spot urine sample in a central laboratory at *Fundacion Jimenez Diaz* in Madrid for the primary outcome and in the local laboratory facility for safety issues. Microalbuminuria will be defined as an urine albumin to creatinine ratio (UACR) of ≥30 mg/g; Albuminuria will be defined with a UACR ≥300 mg/g. Glomerular filtration rate will be estimated (eGFR)by Modification of Diet in Renal Disease (MDRD-4) Study equation and the CKD-EPI equation using serum creatinine levels measured at local laboratory facility (29). The evaluation of urine albumin and creatinine at the central laboratory (IIS-FJD) will be performed mandatorily at baseline, 24 and 36 months visits.

- The primary composite **microvascular complication index** (MCI) will be assessed using a linear combination (weights will be defined in the Statistical Analysis Plan (SAP) and estimated from other studies) of the score of the Early Treatment Diabetic Retinopathy Study Scale (ETDRS), the level of urinary albumin to creatinine ratio, and the sweat function (SUDOSCAN).

6.5 Assessment of secondary endpoints

- ***Microvascular endothelial function by Endo-PAT®*** (Itamar Medical, Caesarea, Israel) is a new technique which can assess endothelial function quickly, easily, reliably and safely. The technique is based on the assumption that increases in digital pulse volume after an arterial occlusion as compared to baseline values, are mediated by endothelial activity. This assumption has been corroborated by evidence from studies in both normal subjects and subjects with known endothelial dysfunction (35). Central reading and analysis will be performed at the Department of Medicine, University of Pisa (WP5). This will be performed only in a subsample of around 1000 subjects that will be defined base on local centres feasibility, at baseline and at 24 and 48 months' visits. However, optionally centres might decide additional evaluations at 12th months visits.

- ***Novel biomarkers*** will be measured in different laboratories from baseline and 24th and 36th months visits in the same subsample selected for cardiovascular assessment with Endo-PAT and IMT. Although the final list of biomarkers to be measured might change during the lifetime of the project, the initial list of the selected biomarkers is as composed by the following:

  - Biomarkers of inflammation and microvascular damage: will be measured at central laboratories Fundación Jiménez Díaz (FJD) in Madrid and DASMAN Institute, Kuwait..

- ***Self-perceived quality of life*** will be assessed by the HRQOL instrument. This instrument is self administered and explores 5 domains with 5 questions and an additional visual analog scale to rate the self perception general health. This instrument has been widely used including studies in diabetes and pre-diabetes (The results will be analyzed by a central analysis at Donau-University Krems, Austria (WP6). This instrument will be applied at baseline, 12th, 24th and 36th months visits.

- **Michigan Neuropathy Screening Instrument**, The self response questionnaire will be mandatory for at baseline, 12th, 24thand 36th months visits.

- ***Peripheral neuropathy defined*** as reduced sweat function (-1SD from baseline in the Sudoscan) plus at least one symptom on the Michigan Neuropathy Screening Instrument (Q 1-7, 11-13)

- ***Depression and anxiety*** will be measured by the Hamilton Rating Scale for Depression (HAM-D) at baseline, 12, 24 and 36 month visits.

- ***Cognition*** will be measured by a short neuropsychological test-batteryl: Montreal Cognitive Assessment (MOCA), Trail making test A and B and Digit-Span forward and backward.at baseline, 12, 24 and 36 month visits.

- ***Insulin secretion rates (ISR) and β-cell glucose sensitivity***. Slope of the insulin secretion/plasma glucose dose-response by C-peptide deconvolution and OGTT modelling

Safety outcomes including Hypoglycemia described in section 10.

6.6 Informed Consent

Prior to any trial-related activity, the investigator must give the subject verbal and written information about the trial in a form that the subject can read and understand. A voluntary, signed and personally dated informed consent form will be obtained from the subject prior to any trial-related activity. In seeking and documenting informed consent, the investigator must comply with the applicable regulatory requirement(s), and adhere to the ICH GCP and the requirements in the Declaration of Helsinki.

The responsibility for seeking informed consent must remain with the investigator or another physician delegated by the investigator and cannot be delegated to a non-medically qualified person. The written informed consent must be signed and personally dated by the person who seeks the informed consent. The information given to the subjects may be given by other related personnel (e.g., a nurse), but the responsible investigator or another physician delegated by the investigator must always sign and date (i.e., obtain) the informed consent. Any local requirements must be followed. A sample of the informed consent is shown in Appendix C.

For participants incapable of reading and signing the written consent form, the consent form will be read aloud for them and additional explanations will be given if needed. After the participant's questions have been answered satisfactory, a thumb print could replace the participant's signature.

If information becomes available that may be relevant to the subject's willingness to continue participating in the trial, the investigator must inform the subject in a timely manner, and a revised written informed consent must be obtained.

Following subject's trial participation, the investigator will advise the subject with regards to the best possible treatment options for his/her hyperglycaemia.

Issues of insurance (protection of clinical trials participants): The Article 3 of the **Directive 2001/20/EC** relating to the implementation of good clinical practice in the conduct of clinical trials on medicinal products for human use requires that a provision be made for insurance or indemnity to cover the liability of the investigator and the sponsor. Art. 6 allows that either the Ethics Committee or the CA will review the provision for indemnity or compensation in the event of inquiry or death attributable to a clinical trial, and any insurance or indemnity to cover the liability of the investigator and sponsor. All patients recruited in the trial will be protected by this Directive. Patients should be insured in all participating countries according to EU and local relevant insurance regulation for clinical trials. Several international insurance companies have been identified and will select the most favourable offer from different international insurance companies.
Incidental findings: None work-package of the project includes invasive and absolutely harmless methods and do not provide any safety problems. We will, however, through the screening process identify individuals with previously undiagnosed diseases like diabetes, hypertension, dyslipidaemia etc. The participants will receive a medical report with their own data as soon as possible following the examination (when the blood samples have been analysed) in a personalized visit with the responsible study investigator, and in case of abnormalities they will be advised to contact their general practitioner or other relevant physician.

Consequences of leaving the study:
Study interventions may be permanently or temporarily discontinued for a particular participant if a definite contraindication attributable to any of the three drugs becomes apparent. The decision to discontinue the study interventions will be at the discretion of the study participant and the responsible physician.
If the subject withdraws the previously given informed consent the subject's data will be handled as follows:

- Data collected will be retained by the Coordination centre and Clinical trial Sponsor (EVIDEM) and entered into the database
- Safety events will be reported to the departments responsible for global product safety of the two drug companies providing the drugs for the trial, and to regulatory authorities according to local/national requirements

If data is used, it will always be in accordance with local law and IRB/IEC procedures.
When a subject's participation in the trial ends, the subject will consult with his/her investigator to decide on the best available treatment.

Data protection issues

Confidentiality of participants will be protected by identifying each participant on all study forms by a unique participant number. The key to the participants' numbers will be kept locked or otherwise inaccessible except to authorised persons or in emergencies. No study forms or other documents collected for the purpose of this study should reveal the participant's name. All collected data will be kept secure. No participant identifiers will be presented on any files transmitted to any committee or any institution.

First part of the data collection will be due to diabetes risk screening programs. All other parts of the project will collect self-reported and doctor-assisted questionnaire data, data obtained by physical examination and laboratory data. Collection of these data will be based on written, informed consent from each participant. The data coordination and monitoring centre will develop the database, the forms and the software to manage and scan the data sheets. The aim is to have an electronic data transfer and a save internet based data input from all regional participating centres. The central laboratory and central genetic lab will prepare and provide standardized lab forms and genetic investigation protocols. Individual data will be identifiable by centre code and registration number in the survey. The translation code between the anonymous patients' data and name, social security number, birth date or other identifier is kept separate in the institution performing the local survey.

The pharmacological intervention is one of the core parts of the study. Two different pharmacological drugs, with different formulations, are used in the study. These two drugs has been previously tested and evaluated in several clinical trials (11,18,19). Intervention is also based on behavioural, psychological based lifestyle recommendations, which are used by national or international professional institutions. Lifestyle recommendations were studied in other projects and were shown not to be harmful for the individuals (8,9)

Patient samples taken for baseline examination and follow up, will be done following the operation manual for this protocol at the performing institution.

There is no part in the ePREDICE Project which involves use of animals, human embryonic stem cells, modifications of the human genome, which then becomes hereditary or which involves cloning of individuals.
The complete protocol will be registered in the EUDRA-CT and Clinical Trial Gov registry. Patients will receive all medications free of charge.

7. STUDY VISITS

(Follow Operation Manual for detailed description in each visit)

7.1 Screening and Eligibility Assessment: Enrolment and Baseline visits

Screening procedures of eligible participants and patient recruitment:

Screening will be performed, in the first place, using any opportunistic screening strategy, either in the community, occupational or primary care setting. If the centre already has a pre-established and ongoing strategy for screening people to detect diabetes and pre-diabetes, databases and clinical records can be reviewed at each recruiting centre before the enrolment visit in order to identify potential eligible subjects for the trial.

Different opportunistic or systematic screening procedures can be used to detect potential eligible population (non-diabetic dysglycaemia) in people aged 45-74 years for this project. We have identified the following as the most common situations:

- People with abnormal non-diabetic (IGT and/or IFG) fasting or 2 hours post glucose load in an OGTT values within the 6 previous months documented in medical records or databases.

- A pre-established systematic or opportunistic screening programs using any risk score available (FINDRISC, Australian, UK risk scores, etc) and performing thereafter an OGTT in those individuals identified as high risk according to specific cut-offs.

- Performing an OGTT specifically for the trial in any individual with known risk factors for developing diabetes (obesity, cardio-metabolic risk factors, family history of diabetes, etc)

Each centre will follow their regular already implemented strategy and invite participants for the first visit of this project to confirm the inclusion and exclusion criteria.

Potential candidates can be contacted by letter, telephone or both for a first evaluation of inclusion/exclusion criteria. If the participant is likely to fulfil the study´s criteria can be invited to the enrolment visit (visit 1).

Enrolment (visit 1). This visit is aimed to evaluate eligibility criteria. A quick interview can be performed to check if the participants fulfil the criteria, mainly based on medical history (exclusion criteria). If based on medical history, the participant is likely to be eligible; subjects must give signed and dated informed consent prior to any subsequent trial-related activities. Subjects must be provided with a copy of the subject information and a copy of their own signed and dated informed consent form. Subjects will be assigned a unique number (lowest available number allocated to the site) which will remain the same throughout the trial.

After signed consent, two important tests are recommended to perform before any other test, to avoid further testing and interviews in ineligible participants:

a) ***Ophthalmological screening assessment***: Single fundus photography of each eye centred in the macula without mydriasis to assure the quality of ocular media for the primary outcome evaluation (presence of cataracts or any other eye problem that make retina visualization difficult). These images

will be sent by email with no personal data of the participant to the central reading centre (WP6) (email address (**screening.wp6@ioba.med.uva.es**) who will respond if this patient is valid to be included in less than 24 hours. The new central reading center of retinal images is eDiagnostic.

b) To evaluate the Inclusion criteria of Pre-diabetes (IGT and/or IGF) condition:

- Perform a standard OGTT (at 0´and 120´) during this visit using the HEMOCUE® device .

In addition to these tests, some centres may want to perform renal (serum creatinine and GFR calculation) hepatic (AST, ALT, ALP, gamma-GT) and pancreatic (Amylase and Lipase) profiles at this time in order to rule-out exclusion criteria.

After reviewing the results of these tests, other interviews and physical examinations can be performed in this visit or complete in visit number 2.

It will depend on the centre preference if the entire set of biological samples for all these analyses is taking on this enrolment visit or in combination with the following visit (visit 2) dedicated to completed the mandatory baseline evaluation with a second blood draw. In general we identify two different strategies:

a) ***If the preference is to have only one blood draw***, blood samples of a complete 5 point OGTT (0, 30, 60, 90 and 120 min) need to be taken during the enrolment visit and prepared for both local and central laboratory procedures. If by the local measurement of glucose levels using only fasting and 120 min samples, the participant fulfil the criteria for IGT and/or IFG, the participant will be invited to participate and, if accept, all samples from the full OGTT need to be analysed locally (including all local laboratory assessment) and shipped to central laboratory for central analysis to complete the baseline including the samples for DNA. If the participant does not classify as pre-diabetes or does not want to participate, samples should be discharged.

b) ***If the preference is to have two independent blood draws***, in visit 1 (enrolment), a simplified OGTT with only local glucose measurement at two points (fasting and 2 hours) will be performed as screening, and if the participant classifies as IGT and/or IFG and accepts to participate in the study, the participant will be invited to visit 2 (baseline evaluation) where a second blood draw will be performed including a complete (5 points) OGTT and other local and central laboratory analysis to complete the baseline evaluation.

All plasma glucose assessments will be performed at local clinical centres using **mandatorily the HEMOCUE® system** at the point of care to avoid variations across centres explained by differences in local procedures.

Note: OGTTs used for screening purposes (simplified version with only 2 time points at 0 and 120 minutes) can be performed using **capillary or venous** blood samples with the HEMOCUE® system. However any other OGTT, including full OGTT (5 time points) at baseline and final visit at month 24th and other simplified versions at months 12 and 24 (see below) **venous whole blood is mandatory**.
Because of the expected intra-person variability in glucose levels, it is likely that participants who have a previous OGTT fulfilling the criteria for pre-diabetes (IGT or IFG), in a second evaluation may have different classification, either normoglycemic or T2D. To avoid an important number of screening failures, the final inclusion criteria for glucose levels at fasting and two hour post glucose load will be based on the average of the two OGTTs

performed. This will include the baseline the OGTT (with 5 time points) and the previous OGTT (only of 2 time points) if it was used for screening. .

The following table describes the possible combinations of the two OGTTs previous to randomisation and final decision based on their results:

**Possible combination**	**First OGTT (2 points)**	**Second OGTT (5 points, but using only fasting and 2 hrs for classification)**	**Final decision**
1	Normal	--	These participants will not be invited for baseline evaluation and will not be included nor randomised in the trial.
2	Diabetes	--	These participants will not be invited for baseline evaluation and will not be included nor randomised in the trial.
3	IGT or IFG	IGT or IFG (confirmed)	Invitation to complete baseline evaluation and randomisations.
4	IGT or IFG	Normal	Use the **average** of between the 2 assessments to classify the participant. If the average values fulfil the criteria of IGT or IFG invite the participant for complete baseline evaluation and randomisation.
5	IGT or IFG	Diabetes	As the standard of care guidelines, the potential diagnosis of Diabetes needs to be confirmed by a second evaluation. Therefore, these participants need to be sent to their regular care centre or physician for a confirmatory test. If diabetes diagnosis **is not** confirmed, clinical centres might decide to invite these participants to the trial and complete baseline evaluation and randomisation if the **average** between the 3 previous assessments, fulfil the criteria of IGT or IFG.

Once the inclusion criteria based on the OGTT evaluation and screen retinogram are confirmed and a first review of the exclusion criteria by medical history are discarded, participant should be invited to a second visit to complete baseline evaluation, confirm inclusion/exclusion criteria, complete assessment of primary outcomes and randomise the participant to a specific treatment arm.

As mentioned before, the entire baseline evaluation can be performed within visit 1 and visit 2; however the analysis of all inclusion and exclusion criteria, including the laboratory analysis to evaluate, renal, liver and pancreatic profiles, must be performed in **no more than 1 month** before randomisation.

The following table describe all procedure to be completed as baseline evaluation before randomisation:

Procedure	Name	Description	Visit 1 and 2<br>m 0-1<br>Enrol/Baseline
CRFs	**Review inclusion / exclusion Criteria**		M
	**Informed consent (IC)**		M
	**Ophthalmic screening assessment**		M
	**Medical interview**	Personal data, personal and family medical history, background lifestyle	M
	**Physical examination**	Height, weight, waist and hip circumference, blood pressure	M
	**Drug Accountability Log**		M
	**Lifestyle**	Nutrition Qx, physical activity (RPAQ)	M
		Physical activity diary	M
	**Neuropsychological assessment**	Quality of life (5D)	M
		Cognition (MOCA, TMT AB, Digit Span – Forward and Backward)	M
		Extended test series	O
		Anxiety and depression (Hamilton and MINI)	M
		Sleep disorders questionnaire	M
		Neuropathic symptoms (MNSI_ Scoring Version)	M
Local Laboratory	**Hemogram**		M
	**Complete OGTT (glucose)**	0, 30, 60, 90 and 120 min glucose	M
	**Simplified OGTT (screening)**	0 and 120 minutes	O
	**HbA1c**		M
	**Hepatic profile**	AST, ALT, ALP, gamma-GT	M
	**Renal profile**	Creatinine, uric acid	M
	**Lipid profile**	Total-Cholesterol, HDL-Cholesterol, Triglycerides	M
	**Morning urine**	Albuminuria, creatinine.	M
	**pregnancy test**		M
Central Lab FJD SPAIN	**Insulin and C-petide**	0, 30, 60, 90 and 120 min	M
	**Special Biomarkers**		M
	**Morning urine**	Urine Albumin and creatinine	M
	**DNA sample**		M
Diagnostic Procedure	**Retinography**	Retinal vasculature	M
	**SUDOSCAN**	Sudomotor index	M
M: Mandatory, O: Optional.			

All central laboratory analysis, focused on the measurement of all biomarkers needed for the primary and secondary outcomes evaluation including special biomarkers, will be performed at the end of the trial for both baseline and final visits (month 24th and 36th). However, the clinical centre will need to follow the operation manual to ship the samples periodically to the central laboratory. Local laboratory assessments are focused on glucose monitoring and safety evaluation and will be performed in routine bases during follow up, dependent of the programmed test. (Visit plan Appendix A of protocol V5.0).

All laboratory results will be reviewed and the reports signed by the Investigator who will record in the CRF whether they are normal, abnormal but NOT clinically significant or abnormal AND clinically significant. In the case the pancreatic, hepatic and renal profiles will be repeated before the Baseline visit, and if the abnormal result(s) confirmed, the patient will be excluded.

Specific Biomarkers:

- *Biomarkers of inflammation and microvascular damage*

7.2 Subsequent Assessments

Visit 3	Visit 4	Visit 5	Visit 6	Visit 7
3 Month	6 Month	12 Month	24 Month	36 Month

For each visit, the evaluation includes

- Assessment of endpoints/outcome measures according to work plan
- Physical examination, basic laboratory tests and safety assessment.
- Dispensing study drugs.
- Assessment of drug compliance and life-style intervention adherence and concomitant medication.

Visits contents

Visit 3 (At 3 months**:** Core assessment)

Procedure	Name	Description	Visit 3<br>m 3
CRFs	Medical Interview	Patient's personal data and update background information	M
	Physical examination	Weight, waist circumference and blood pressure (BP)	M
	Drug Accountability Log		M
	Adherence Questionnaire	Includes pill count	M
	Lifestyle	workshops record	M
		Individual intervention form	M
M: Mandatory, O: Optional			

Visit 4 (At 6 months**:** Core, assessment)

Procedure	Name	Description	Visit 4<br>m 6
CRFs	Medical Interview	Patient's personal data and update background information	M
	Physical examination	Weight, waist circumference and blood pressure (BP)	M
	Drug Accountability Log		M
	Adherence Questionnaire	Includes pill count	M
	Lifestyle	Nutrition and physical activity questionnaire, RPAQ	M

		workshop records and individual intervention forms	M
Local Laboratory	fasting glucose<br>HbA1c		M<br>M
M: Mandatory,			

Visit 5 (At 12 months: Core assessment)

Procedure	Name	Description	Visit 5<br>m 12
CRFs	Medical Interview<br>Physical examination<br>Drug Accountability Log<br>Adherence Questionnaire	Patient's personal data and update background information<br>Height Weight, waist circumference blood pressure (BP)<br><br>Includes pill count	M<br>M<br>M<br>M
	Lifestyle	Nutrition and physical activity (RPAQ) questionnaire<br>workshop records and individual intervention forms	M<br>M
	Neuropsychological assessment	Quality of life (5D)<br>Cognition (MOCA, TMT AB, Digit Span – Forward and Backward)<br>Anxiety and depression (Hamilton and MINI)<br>Neuropathic symptoms (MNSI_ Scoring Version)	M<br>M<br>M<br>M
Local Laboratory Test	Hemogram<br>Simplified OGTT<br>HbA1c<br>Hepatic profile<br>Pancreatic profile<br>Renal profile<br>Lipid profile<br>Morning urine spot	 0 and 120 minutes<br><br>AST, ALT, ALP, gamma-GT<br>Amylase, Lipase<br>Creatinine, uric acid, MDRD-4, CKD-EPI<br>Total-Cholesterol, HDL-Cholesterol, Triglycerides, LDL-Cholesterol<br>albuminuria, creatinine, alb/creat ratio	M<br>M<br>M<br>M<br>O<br>M<br>M<br>M
Diagnostic Procedure	SUDOSCAN	Sudomotor index	M
M: Mandatory, O: Optional			

Visit 6 (At 24 months, complete assessment)

Procedure	Name	Description	Visit 6<br>m 24
CRFs	Medical Interview<br>Physical examination<br>Drug Accountability Log<br>Adherence Questionnaire	Patient's personal data and update background information<br>Height, weight, waist circumference blood pressure (BP)<br><br>Includes pills count	**M**<br>**M**<br>**M**<br>**M**

	**Lifestyle**	Nutrition questionnaire<br>physical activity (RPAQ)<br>workshop records and individual intervention forms	M<br>M<br>M
	**Neuropsychological assessment**	Quality of life (5D)<br>Cognition (MOCA, TMT AB, Digit Span – Forward and Backward)<br>Depression (Hamilton)<br>Sleep disorders<br>Neuropathic symptoms (MNSI_ Scoring Version)	M<br>M<br>M<br>M<br>M
Local Laboratory Test	**Hemogram**		M
	**Complete OGTT**	0, 30, 60, 90 and 120 min glucose	M
	**HbA1c**		M
	**Hepatic profile**	AST, ALT, ALP, gamma-GT	M
	**Pancreatic profile**	Amylase, Lipase	O
	**Renal profile**	Creatinine, uric acid, MDRD-4, CKD-EPI	M
	**Lipid profile**	Total-Cholesterol, HDL-Cholesterol, Triglycerides,, LDL-Cholesterol	M
	**Morning urine spot**	albuminuria, creatinine, alb/creat ratio	M
FJD SPAIN	**Insulin and C-peptide**	0, 30, 60, 90 and 120 min	M
	**Biomarkers**		M
	**Morning urine**	albuminuria, creatinine, alb/creat ratio	M
Diagnostic Procedure	**Retinography**	Retinal vasculature	M
	**SUDOSCAN**	Sudomotor index	M
**M: Mandatory, O: Optional**			**CA.**

7.3 End of Trial Assessment (Observational follow up after open experimental treatment)

After 24 month of experimental treatment, the randomised treatment will be unmasked and the participant will be referred to his/her primary physician to continue treatment as needed. Between month 24 and month 36, clinical centre will contact the participant by telephone at least once at 36 months. All participants should be evaluated here, including those who have dropped out from their allocated treatment for any reason. This visit will be cover with global and local funds to be identified later on

Visit 7 (At 36 months, complete assessment)

Procedure	Name	Description	Visit 7<br>m 36
CRFs	**Medical Interview**	Patient's personal data and update background information	M
	**Physical examination**	Height, weight, waist circumference blood pressure (BP)	M
	**Lifestyle**	Nutrition and physical activity (RPAQ) questionnaire	M<br>M

	**Neuropsychological assessment**	Quality of life (5D)<br>Cognition (MOCA, TMT AB, Digit Span – Forward and Backward)<br>Depression (Hamilton)<br>Sleep disorders<br>Neuropathic symptoms (MNSI_ Scoring Version)	**M**<br>**M**<br>**M**<br>**M**<br>**M**
**Local Laboratory Test**	**Hemogram**		**M**
	**Complete OGTT**	0, 30, 60, 90 and 120 min glucose	**M**
	**HbA1c**		**M**
	**Hepatic profile**	AST, ALT, ALP, gamma-GT	**M**
	**Pancreatic profile**	Amylase, Lipase	**O**
	**Renal profile**	Creatinine, uric acid, MDRD-4, CKD-EPI	**M**
	**Lipid profile**	Total-Cholesterol, HDL-Cholesterol, Triglycerides, LDL-Cholesterol	**M**
	**Morning urine spot**	albuminuria, creatinine, alb/cret ratio	**M**
**FJD SPAIN**	**Insulin and C-petide**	0, 30, 60, 90 and 120 min	**M**
	**Biomarkers**		**M**
	**Morning urine**	albuminuria, creatinine, alb/cret ratio	**M**
**Diagnostic Procedure**	**Retinography**	Retinal vasculature	**M**
	**SUDOSCAN**	Sudomotor index	**M**
**M: Mandatory, O: Optional**			

8. INTERVENTIONS

8.1 Intervention Groups:

After randomization patients will be assigned to any of the following 4 intervention groups: (see table 2)

Table 2: Intervention Groups

INTERVENTION GROUPS	
1	**Lifestyle intervention + one placebo tablet/twice a day**: One during breakfast, one during dinner). Placebo pills with identical aspect of linagliptin 5 mg active drug pill
2	**Lifestyle intervention + *Metformin* 850 mg/ twice a day;** One during breakfast, one during dinner)**.** No placebo pills.
3	**Lifestyle intervention + *Linagliptin* 5mg/day:** One tablet of active drug during breakfast and one placebo tablet during dinner. Placebo pills with identical aspect of linagliptin 5 mg active drug pill
4	**Lifestyle intervention** + **fixed-dose combination of *Linagliptin* 2.5mg with *Metformin* 850 mg/twice a day**: One during breakfast, one during dinner. No placebo pills.

Rescue therapy:

If in any of the 4 groups, HbA1c >6.5% or fasting glucose ≥7.0 mmol/l or 2 hr post load glucose ≥11.1 mmol/l is observed in two consecutive study visits (including a documented laboratory results independent the pre-established study protocol visits), the patient will be refer to his/her primary care physician to confirm diabetes diagnosis and if needed individualized therapy will be added, depending on the decision of the physician. The blinded experimental therapy will be open (unmasked) in order to provide all relevant information to the primary care physician to decide the individualized rescue therapy. A letter to primary care physician will be provided with an explanation of the trial, encouraging to favour the maintenance of the experimental treatment (that will be still provided by the trial) and add any glucose lowering treatment that will not interfere with the experimental therapy, avoiding the use of metformin and DPP4 inhibitors. Patient´s clinical follow-up (and outcome evaluation) will be kept unchanged and the added therapy will be recorded and considered in the finally analysis. If the regular primary care physician is the physician in charge of the follow up of the trial or the participant does not have a regular primary care physician, the individualized therapy can be added by the trial´s physician.

If pharmacological treatment for other CVD risk factors (blood pressure or lipid lowering drugs) becomes indicated over the course of the trial, therapy can be prescribed at the discretion of the responsible physician. All patients in groups 1, 2, 3 and 4 will be also enrolled in lifestyle intervention component.

8.2 Lifestyle intervention

A motivational intervention program will be set up for all the participants. This program will follow the process model for supporting lifestyle behaviour changes proposed by the EU-funded IMAGE project (*"toolkit to change behaviour": http://www.image-project.eu/pdf/final_version_of_toolkit-perfect.pdf*) The main objective of the proposed program is to give advice and support in developing motivation to make individually tailored lifestyle changes, goal setting and action planning and to facilitate maintenance of achieved lifestyle changes. Helping a person to change an existing behaviour requires: a) Individually tailored intervention and advice, b) support for developing motivation to make changes, goal-setting & action planning and c) ongoing support and encouragement to maintain change & advice on how to manage setbacks. It is particularly important to aim at achieving several healthy lifestyle habits.

A process model for supporting lifestyle behavior change (Source: G*reaves & Sheppard, 2009 (26))***:**

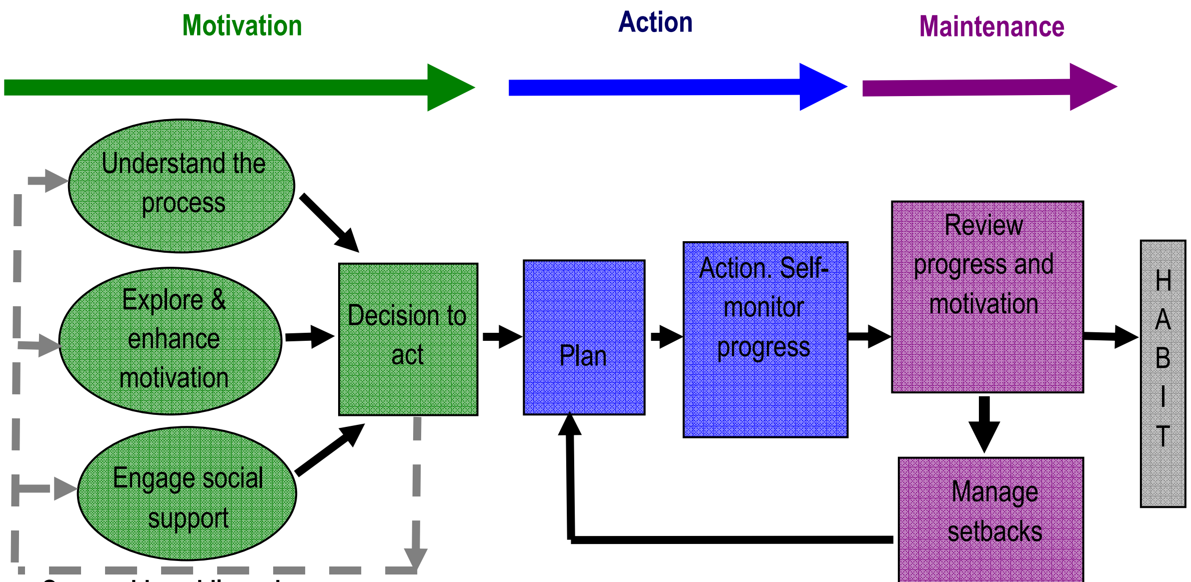

Over-arching philosophy:
Emphasise empowerment / ownership of goals, risk and actions
Develop tools, strategies and motivation to manage lifestyle in the long term

The intervention programme for behaviour change includes

- Support for changes in diet and physical activity, and smoking cessation
- Support for self-monitoring & self-regulation - encourage individuals to monitor physical activity levels and what they eat (activity and food diaries) & encourage personal management of their behaviour change (i.e. regular reflection on progress with a focus on identifying what works and problem solving).
- Goal-setting - plan when, where, and how to perform the new behaviour. Ensure goals are SMART: Specific, Measurable, Achievable, Relevant, and Time-framed. Consider setting both short-term goals and long-term goals
- Action planning – An action plan should include three sections:
    - Clear goals
    - Clear information on who is providing social support, where and when the support will happen and

  - Coping strategies on how to deal with problems that may occur
- Coping strategies & problem solving – explain how to identify and cope with barriers that stop individuals achieving their goals, and how to solve/ deal with problems when they occur (i.e. how their plans can be revised to work better).
- Social support - engage others who are important such as family, friends and peers, to help support / encourage behaviour change. Support may be emotional, practical or informational (e.g. help with planning activities). Encourage clients to invite a supporter along to sessions if they wish.
- A strong focus on strategies which help maintain changes in behaviour – for example, using relapse management strategies (e.g. explaining that setbacks are normal and provide useful learning experiences, identify what has caused any setbacks and make new coping plans to deal with these barriers to change).

Nutrition and dietary guidance to prevent diabetes

The participants are advised to consume a balanced, nutritious, enjoyable diet, without forgetting pleasure and psychological and social well-being. The intervention goals are translated to food intake level (see goals for food intake in table 3) and diet composition will be individually adjusted (see the EAT CLEVER principle: Table 4)

Table 3. Goals for food intake

GOALS FOR LONG TERM FOOD INTAKE	GOALS FOR LONG-TERM NUTRIENT INTAKE
• Consuming fruit, vegetables, and legumes in abundance (≥ 500 g or five portions per day)<br>• Choosing whole grain in all cereal products<br>• Limit sugar to ≤ 50 g/day, including sugar in food and beverages<br>• Consuming vegetable (olive, rapeseed) oil and/or soft margarines and/or nuts as the primary source of fat<br>• Limiting butter, other saturated fat and partially hydrogenated fats<br>• Choosing low-fat milk and meat products<br>• Consuming fish regularly (≥2 per week)<br>• Consuming alcoholic beverages in moderation (≤2 drink/day for men and ≤1 drink/day for women) if at all<br>• Other goals according to individual needs (e.g. body weight, lipid disorders, hypertension, medications)	• Dietary fibre 25-35 g/day<br>• Total fat 30-35 E%* (60-80 g/day with 2000 kcal daily intake level), of which saturated + trans fat ≤ 10 E%<br>• Alcohol ≤ 5 E%*<br>• Energy intake balanced with physical activity levels to achieve 5% weight reduction or BMI <25<br><br>*E% = proportion of total energy

Table 4: The EAT CLEVER principle: brief practical advice for counsellors.

EAT CLEVER	
**E**stimation of the dietary pattern compared to the recommendations	Use the food diary, or interview to help participant to become aware of his/her dietary pattern and food consumption. Compare dietary intake to the recommendations. Consider special needs, resources and readiness to change food habits.
**A**ims in the long and short term	Discuss both short and long term goals: what is the participant willing and able to do at the moment? Help to set practical, achievable targets and proceed with small steps. Make a plan with the participant.
**T**ools, guidance and	Which kind of tools, guidance, support or skills are needed and available? Involving

support	the family and friends and group counselling are all worth considering.
**C**omposition of the diet	A diet with high sugar and other refined carbohydrates and low fibre content, or high saturated and trans fat content may increase the risk for diabetes and other related disorders. Whole grains and moderate amounts of coffee and alcohol may decrease the risk. Encourage the use of herbs and spices to reduce salt. Refer to your national nutrition recommendations but consider the special requirements of people with high diabetes risk, such as the improvement of the components of the metabolic syndrome. Take into account any additional disease the participant may have.
**L**ifestyle for the whole life	Diet is influenced by culture, religion, ethical, physiological, psychological, social and economic aspects, availability, and individual likes and dislikes. Help the participant to find his/her own healthy way of life. Lifestyle change is a process and relapses are part of it. Help the participant to learn from these experiences to develop successful strategies over time.
**E**nergy	Excessive energy intake causes weight gain. If the participant is overweight, make a plan with her / him to support gradual weight loss (step by step). Focus on substituting foods with high saturated fat and/or refined carbohydrate content with lower-energy items. How many meals and snacks, beverages and alcohol included, does he/she have during a day and night? Some regularity in the daily meal plan helps to control over-eating.
**V**ariety	Emphasise variety instead of restriction. A health-promoting diet provides satiety and pleasure as well as protective nutrients. Encourage the participant to try new foods. Give advice on how to read food labels. This can help the participant to feel more confident and expand their healthy food choices.
**E**valuation	Evaluation and self-monitoring help in achieving and maintaining new food habits. Body weight and /or waist circumference should be measured regularly. Encourage the participant to use a food diary or some other methods to monitor eating habits: the number of meals and snacks, the amounts of certain food stuffs, such as vegetables, whole grains, sugar, alcoholic beverages, vegetable oil and/or fat etc.
**R**isk management	Dietary guidance must be based on evidence from nutrition and behavioural sciences. Focus on the big picture: changing one aspect in the diet affects many others. Strict restrictions and 'crash dieting' may lead to an unhealthy diet, and can cause damage in the long term as well as psychological and social harm. A multi-disciplinary team, including a registered dietician and a psychologist, can give essential support to avoid these risks.

Physical activity to prevent diabetes

It is important to motivate the participant o start, and then to keep being physically active. The following advice/ key messages will help to give adequate support:

- Increase awareness of the need to be active
- Discuss participant´s history of physical activity
- Discuss pros and cons of increasing physical activity
- Help to set realistic and individual goals
- Increase daily physical activity, because every single bout of physical activity is useful (e.g. walking, gardening, etc.)
- Advise the participant to be physically active for at least 30 min on a minimum of 5 days a week
    - Preferably a combination of cardio-respiratory endurance training and resistance training
- Encourage the adoption of enjoyable physical activities as having fun is an important factor in keeping active
- If the participant has co-morbidities recommend that they have a physician consultation before starting vigorous exercise

To educate about how to gain training benefits from a combination of cardiorespiratory endurance training and resistance training, we can apply the F.I.T.T. principle (Table 5). These are general guidelines for individuals of moderate fitness level and are based on “optimal figures”. It may be necessary to break down the recommendations into gradual steps in order to avoid physical and mental overload. Remember that ANY increase in physical activity from the patient´s baseline level is likely to be beneficial.

F.I.T.T. PRINCIPLE		AEROBIC ENDURANCE TRAINING	RESISTANCE TRAINING
**F** requency	How often	3x / week (minimum)<br><br>Max. 2 days gap between training sessions	2-3x / week
**I** ntensity	How hard	(a) light to moderate<br>(40-60% VO2 max. / 50-70% HRmax)<br>(e.g. brisk walking – 5-6 km/h)<br>‡ slightly increased breathing rate<br><br>(b) vigorous<br>e.g. jogging – 8-10 km/h)<br>‡ increased breathing rate and sweating	light to moderate<br>(slight muscular fatigue)
**T** ime	How long	(a) light to moderate<br>45-60 min (in total > 150 min / week)<br><br>(b) vigorous 30-40 min (in total > 90 min / week)	1-3 sets of 8-15 repetitions for each exercise
**T** ype	What kind	‡ walking, jogging, cycling, swimming, hiking, skiing	about 8 different strength exercises<br>‡ using the major muscles of the body<br>(e.g. with fitness machines, resistance-bands or just with your own body weight

Intervention strategy:

All patients will be enrolled in an intensive lifestyle intervention program (group 1 only in intensive lifestyle intervention, and groups 2, 3 and 4 in intensive lifestyle intervention plus the corresponding random allocated drug). The lifestyle intervention will be provided by nursing staff (previously trained by expert psychologists), physical activity and nutrition specialists. The intervention contains informative (diet, exercise) as well as behavioural and motivational modules and consists of the following sessions:

Individual counselling sessions: 2 (+ optional 1) of 1 hour duration each.

- The first individual counselling session is scheduled after the first group session
    - Revision of the ePREDICE lifestyle goals
    - Discussion of the participants´ perceptions of his/her own lifestyle

- **Personal goals** according to participants´ needs and perceptions
- The facilitators help in the goal-making process, but **do not** decide, what the participants´ goals are.
- The progress is reviewed in the next individual counselling session

Group sessions: Small-group (up to 10 participants, 1,5hr each).

- Once a month during the first 6 months (7 sessions) aimed at:
  - motivational activities
  - behavioural guidance
  - information
- 3-monthly sessions thereafter up to the end of the intervention (10 session in total) aimed at:.
  - monitoring the progress
  - sharing experiences
  - building and maintaining motivation and perceived self-efficacy

The physical activity intervention program: Physical activity will be strongly emphasized during the group sessions, where participants are encouraged to be physically active in their everyday life and to exercise also on their own. Optionally, based on recruitment centre resources, physical activity intervention can be delivered by group coaching (two sessions per week starting from month 3) and facilitate access to local gyms and sport clubs The lifestyle programme will be adapted to the local circumstances but keeping the same goals .

The specific goals of the intervention and the basis for the evaluation are as follows:

1) ≥ 150 minutes of aerobic exercise per week
2) ≥ 2 strength exercises per week
3) ≥ 5 servings of vegetables every day
4) ≥ 3 servings of fruit every day
5) ≥ 4 servings (60 ml) of extra virgin olive or rapeseed oil per day
6) ≥ 3 servings (90 g) of nuts per week
7) ≥ 4 servings of whole-grain foods per day (instead of the white ones)
8) ≥ 1 serving low-fat dairy products (instead of the normal fat ones)
9) ≤ 1 servings of sweets, biscuits, cakes, chocolate per week
10) ≤ 1 servings of fast food (hamburgers, chips, pizza, savoury pastries) per week
11) < 1 servings of sugared drinks (sodas and juices) per day
12) ≤ 2 servings of red meat and meat products per week
13) Weight loss of >5% of the baseline body weight

8.3 Drug intervention

As described before, three out of the four arms in the trial will receive active drug therapy in addition to the life-style intervention. The remaining arm will only receive the life-style intervention plus placebo pills. The three active drugs to be used in this trial are Metformin, provided by Merck Serono®, Linagliptin and a fixed dose combination of Metformin + Linagliptin both provided by BOEHRINGER INGELHEIM. As an Investigator driven clinical trial, both pharmaceutical companies (Merck Serono® and BOEHRINGER INGELHEIM) are not involved in any scientific decision and will only participate by donating, preparing and distributing the drugs.

Both companies will donate the medication as bulk of tablets and ship them to the Monitoring and Logistic Centre EVIDEM CONSULTORES) in Madrid. An authorized pharmaceutical laboratory in Madrid will prepare the active drug and placebo pills in individual masked standard pharmacy bottles (identical for all treatment arms) that will be distributed to all centres following the planned visits (see appendix A: Visit Plan): The first 2 bottles pills for 3 months each, and afterwards 5 bottles for medication for 6 months each to complete the 24 months of the experimental intervention.

In addition to adherence questionnaire and pill count, an objective measurement of drug adherence will be evaluated by using Medication Event Monitoring System (MEMS®) by AARDEX® group, a partner of the ePredice consortium. The MEMS® integrates a microcircuit that registers time and date of every opening and closing. These special devices will be used for a subsample of 20% of all participants stratified by clinical centres.

Because of frequent gastrointestinal side effects of metformin (included in 2 out of the four arms) and to avoid discontinuation of the treatment, the drug should be started at a low dose with gradual titration. To maintain the blind design, we will use the same titration strategy in all four arms, starting with only one pill during the first two weeks and increase to the full dose of 2 pills per day (as described in table of intervention groups above).

8.3.1: Metformin 850mg (provided by Merck Serono® (*Glucophage*)).
(For complete product information released by the European Medicines Agency see appendix B):

Each film-coated tablet contains metformin hydrochloride 850 mg corresponding to metformin base 662,9 mg.

Metformin is a biguanide with antihyperglycaemic effects, lowering both basal and postprandial plasma glucose. It does not stimulate insulin secretion and therefore does not produce hypoglycaemia. Metformin may act via 3 mechanisms: (1) Reduction of hepatic glucose production by inhibiting gluconeogenesis and glycogenolysis, (2) In muscle, by increasing insulin sensitivity, improving peripheral glucose uptake and utilization and (3) Delay of intestinal glucose absorption.

Posology and method of administration
In addition to Lifestyle intervention, Group 2 will receive monotherapy with **Metormin 850 mg/ twice a day** (one during or after breakfast and one during or after dinner). Because of frequent gastrointestinal side effects, it should be started at a low dose with gradual titration
.
Contraindications
Hypersensitivity to metformin hydrochloride or to any of the excipients.

- Diabetic ketoacidosis, diabetic pre-coma.
- Renal failure or renal dysfunction (e.g., serum creatinine levels > 135 µmol/L in males and > 110 µmol/L in females).
- Acute conditions with the potential to alter renal function such as: -Dehydration, -severe infection, -shock, -intravascular administration of iodinated contrast agents.
- Acute or chronic disease which may cause tissue hypoxia such as: -cardiac or respiratory failure, -recent myocardial infarction, -shock
- Hepatic insufficiency, acute alcohol intoxication, alcoholism.

Special warnings and special precautions for use

- **Lactic acidosis:** Lactic acidosis is a rare, but serious (high mortality in the absence of prompt treatment), metabolic complication that can occur due to metformin accumulation. Reported cases of lactic acidosis in patients on metformin have occurred primarily in diabetic patients with significant renal failure. The incidence of lactic acidosis can and should be reduced by assessing also other associated risk factors such as poorly controlled diabetes, ketosis, prolonged fasting, excessive alcohol intake, hepatic insufficiency and any condition associated with hypoxia.
- **Renal function:** As metformin is excreted by the kidney, serum creatinine levels should be determined before initiating treatment and regularly thereafter:
  – at least annually in patients with normal renal function,
  – at least two to four times a year in patients with serum creatinine levels at the upper limit of normal and in elderly subjects.

  Decreased renal function in elderly subjects is frequent and asymptomatic. Special caution should be exercised in situations where renal function may become impaired, for example when initiating antihypertensive therapy or diuretic therapy and when starting therapy with an NSAID.

Undesirable effects

- Gastrointestinal symptoms such as nausea, vomiting, diarrhea, abdominal pain and loss of appetite (>10%) are very common: these occur most frequently during initiation of therapy and resolve spontaneously in most cases. To prevent these gastrointestinal symptoms, it is recommended that metformin be taken in 2 or 3 daily doses during or after meals. A slow increase of the dose may also improve gastrointestinal tolerability.
- Metallic taste (3 %) is common.
- Mild erythema has been reported in some hypersensitive individuals. The incidence of such effects is regarded as very rare (<0.01%)
- A decrease of vitamin B12 absorption with decrease of serum levels has been observed in patients treated long-term with metformin and appears generally to be without clinical significance (<0.01%).
- Lactic acidosis (0.03 cases/1000 patient-years) is very rare

8.3.2: Linagliptin (provided by Boehringer Ingelheim**)**
(For complete product information released by the European Medicines Agency see appendix B):

Each tablet contains linagliptin (as phosphate monohydrate), equivalent to 2.5 mg linagliptin.
Linagliptin is an inhibitor of DPP-4, an enzyme that degrades the incretin hormones glucagon-like peptide-1 (GLP-1) and glucose-dependent insulinotropic polypeptide (GIP). Both GLP-1 and GIP increase insulin biosynthesis and secretion from pancreatic beta cells in the presence of normal and elevated blood glucose levels. GLP-1 also reduces glucagon secretion from pancreatic alpha cells, resulting in a reduction in hepatic glucose output. Thus, linagliptin stimulates the release of insulin in a glucose-dependent manner and decreases the levels of glucagon in the circulation.

Posology and method of administration
In addition to Lifestyle intervention, Group 3 will receive monotherapy with **Linagliptin 2.5 mg c/12h** (during breakfast and dinner).
Contraindications: Hypersensitivity to the active substance or to any of the excipients

Special warnings and precautions for use

- Linagliptin should not be used in patients with type 1 diabetes or for the treatment of diabetic ketoacidosis.
- ***Pancreatitis:*** In post-marketing experience there have been spontaneously reported adverse reactions of acute pancreatitis. Patients are informed of the characteristic symptom of acute pancreatitis: persistent, severe abdominal pain. Resolution of pancreatitis has been observed after discontinuation of linagliptin (with or without supportive treatment), but very rare cases of necrotising or haemorrhagic pancreatitis and/or death have been reported. If pancreatitis is suspected, Linagliptin and other potentially suspect medicinal products should be discontinued.
- ***Hypoglycaemia***: In clinical trials of Linagliptin as monotherapy and as part of combination therapy with medicinal products not known to cause hypoglycaemia (i.e. metformin and/or a PPARγ agonist), rates of hypoglycaemia reported with linagliptin were similar to rates in patients taking placebo. When linagliptin was added to a sulphonylurea or to insulin, the incidence of hypoglycaemia was increased over that of placebo).
- ***Renal impairment:*** Linagliptin is renally excreted. To achieve plasma concentrations of Linagliptin similar to those in patients with normal renal function, lower dosages are recommended in patients with moderate and severe renal impairment, as well as in ESRD patients requiring haemodialysis or peritoneal dialysis.
- ***Hypersensitivity reactions***: Postmarketing reports of serious hypersensitivity reactions in patients treated with Linagliptin have been reported. These reactions include anaphylaxis, angioedema, and exfoliative skin conditions including Stevens-Johnson syndrome. Onset of these reactions occurred within the first 3 months after initiation of treatment with Linagliptin, with some reports occurring after the first dose. If a hypersensitivity reaction is suspected, discontinue Linagliptin, assess for other potential causes for the event, and institute alternative treatment for diabetes.

Undesirable effects

Serious adverse reactions including pancreatitis and hypersensitivity reactions have been reported.

8.3.3 Fixed-dose combination of linagliptin with metformin JENTADUETO (provided by BOEHRINGER INGELHEIM

Each tablet contains 2.5mg of linagliptin (as phosphate monohydrate) and 850 mg of metformin hydrochloride.

Combines two antihyperglycaemic agents with complementary mechanisms of action to improve glycaemic control: linagliptin phosphate, a dipeptidyl peptidase 4 (DPP-4) inhibitor, and metformin hydrochloride, a member of the biguanide class.

Pharmacokinetic properties

A bioequivalence study in healthy subjects demonstrated that the linagliptin/metformin hydrochloride combination tablets are bioequivalent to co-administration of linagliptin phosphate and metformin hydrochloride as individual tablets.

Posology and method of administration

In addition to lifestyle intervention, group4 will receive **2 tablets of a fixed-dose combination of *Linagliptin* 2.5mg/ with *Metformin* 850mg c/12h. (one during breakfast, one during dinner).** No placebo pills

Contraindications

Is contraindicated in patients with:

- Renal impairment (e.g., serum creatinine ≥1.5 mg/dL for men, ≥1.4 mg/dL for women, or abnormal creatinine clearance) which may also result from conditions
- such as cardiovascular collapse (shock), acute myocardial infarction, and septicemia [see Warnings and Precautions
- Acute or chronic metabolic acidosis, including diabetic ketoacidosis. Diabetic ketoacidosis should be treated with insulin [see Warnings and Precautions
- A history of hypersensitivity reaction to linagliptin (such as urticaria, angioedema, or bronchial hyperreactivity) or metformin

Special warnings and precautions for use

- Lactic Acidosis

Metformin

Lactic acidosis is a serious, metabolic complication that can occur due to metformin accumulation during treatment with JENTADUETO and is fatal in approximately 50% of cases. Lactic acidosis may also occur in association with a number of pathophysiologic conditions, including diabetes mellitus, and whenever there is significant tissue hypoperfusion and hypoxemia. Lactic acidosis is characterized by elevated blood lactate levels (>5 mmol/L), decreased blood pH, electrolyte disturbances with an increased anion gap, and an increased lactate/pyruvate ratio. When metformin is implicated as the cause of lactic acidosis, metformin plasma levels of >5 µg/mL are generally found.

The reported incidence of lactic acidosis in patients receiving metformin is approximately 0.03 cases/1000 patient-years, (with approximately 0.015 fatal cases/1000 patient-years). In more than 20,000 patient-years exposure to metformin in clinical trials, there were no reports of lactic acidosis. Reported cases have occurred primarily in diabetic patients with significant renal impairment, including both intrinsic renal disease and renal hypoperfusion, often in the setting of multiple concomitant medical/surgical problems and multiple concomitant medications. Patients with congestive heart failure requiring pharmacologic management, particularly when accompanied by hypoperfusion and hypoxemia due to unstable or acute failure, are at increased risk of lactic acidosis. The risk of lactic acidosis increases with the degree of renal impairment and the patient's age. The risk of lactic acidosis may, therefore, be significantly decreased by regular monitoring of renal function in patients taking metformin. In particular, treatment of the elderly should be accompanied by careful monitoring of renal function. Metformin treatment should not be initiated in any patients unless measurement of creatinine clearance demonstrates that renal function is not reduced. In addition, metformin should be promptly withheld in the presence of any condition associated with hypoxemia, dehydration, or sepsis. Because impaired hepatic function may significantly limit the ability to clear lactate, metformin should be avoided in patients with clinical or laboratory evidence of hepatic impairment. Patients should be cautioned against excessive alcohol intake when taking metformin, since alcohol potentiates the effects of metformin on lactate metabolism. In addition, metformin should be temporarily discontinued prior to any intravascular radiocontrast study and for any surgical procedure necessitating restricted intake of food or fluids. Use of topiramate, a carbonic anhydrase inhibitor, in epilepsy and migraine prophylaxis may cause dose-dependent metabolic acidosis and may exacerbate the risk of metformin-induced lactic acidosis

- Monitoring of Renal Function

Although linagliptin undergoes minimal renal excretion, metformin is known to be substantially excreted by the kidney. The risk of metformin accumulation and lactic acidosis increases with the degree of renal impairment. Therefore, JENTADUETO is contraindicated in patients with renal impairment.

Before initiation of therapy with JENTADUETO and at least annually thereafter, renal function should be assessed and verified to be normal. In patients in whom development of renal impairment is anticipated (e.g., elderly), renal function should be assessed more frequently and JENTADUETO discontinued if evidence of renal impairment is present.

Linagliptin may be continued as a single entity tablet at the same total daily dose of 5 mg if JENTADUETO is discontinued due to evidence of renal impairment. No dose adjustment of linagliptin is recommended in patients with renal impairment.

Use of concomitant medications that may affect renal function or metformin disposition:

Concomitant medication(s) that may affect renal function or result in significant hemodynamic change or interfere with the disposition of metformin should be used with caution Radiological studies and surgical procedures:

Radiologic studies involving the use of intravascular iodinated contrast materials (e.g., intravenous urogram, intravenous cholangiography, angiography, and computed tomography) can lead to acute alteration of renal function and have been associated with lactic acidosis in patients receiving metformin. Therefore, in patients in whom any such study is planned, JENTADUETO should be temporarily discontinued at the time of or prior to the procedure, and withheld for 48 hours subsequent to the procedure and reinstituted only after renal function has been confirmed to be normal. JENTADUETO should be temporarily discontinued for any surgical procedure (except minor procedures not associated with restricted intake of food and fluids) and should not be restarted until the patient's oral intake has resumed and renal function has been evaluated as normal.

- Impaired Hepatic Function

Because impaired hepatic function has been associated with some cases of lactic acidosis with metformin therapy, JENTADUETO should generally be avoided in patients with clinical or laboratory evidence of hepatic disease [see Warnings and Precautions.

- Hypoglycemia

Linagliptin

Insulin secretagogues are known to cause hypoglycemia. The use of linagliptin in combination with an insulin secretagogue (e.g., sulfonylurea) was associated with a higher rate of hypoglycemia compared with placebo in a clinical trial [see Adverse Reactions (6.1)]. Therefore, a lower dose of the insulin secretagogue may be required to reduce the risk of hypoglycemia when used in combination with JENTADUETO [see Dosage and Administration (2.2)].

Metformin

Hypoglycemia does not occur in patients receiving metformin alone under usual circumstances of use, but could occur when caloric intake is deficient, when strenuous exercise is not compensated by caloric supplementation, or during concomitant use with other glucose-lowering agents (such as SUs and insulin) or ethanol. Elderly, debilitated, or malnourished patients, and those with adrenal or pituitary insufficiency or alcohol intoxication are particularly susceptible to hypoglycemic effects.

Hypoglycemia may be difficult to recognize in the elderly, and in people who are taking β-adrenergic blocking drugs.

- Vitamin B12 Levels

In controlled, 29-week clinical trials of metformin, a decrease to subnormal levels of previously normal serum vitamin B12 levels, without clinical manifestations, was observed in approximately 7% of metformin-treated patients. Such decrease, possibly due to interference with B12 absorption from the B12-intrinsic factor complex, is, however, very rarely associated with anemia or neurologic manifestations due to the short duration (<1 year) of the clinical trials. This risk may be more relevant to patients receiving long-term treatment with metformin, and

adverse hematologic and neurologic reactions have been reported postmarketing. The decrease in vitamin B12 levels appears to be rapidly reversible with discontinuation of metformin or vitamin B12 supplementation. Measurement of hematologic parameters on an annual basis is advised in patients on JENTADUETO and any apparent abnormalities should be appropriately investigated and managed. Certain individuals (those with inadequate vitamin B12 or calcium intake or absorption) appear to be predisposed to developing subnormal vitamin B12 levels. In these patients, routine serum vitamin B12 measurement at 2- to 3-year intervals may be useful.

- Alcohol Intake

Alcohol is known to potentiate the effect of metformin on lactate metabolism. Patients, therefore, should be warned against excessive alcohol intake while receiving

- Hypoxic States

Cardiovascular collapse (shock) from whatever cause (e.g., acute congestive heart failure, acute myocardial infarction, and other conditions characterized by hypoxemia) have been associated with lactic acidosis and may also cause prerenal azotemia. When such events occur in patients on JENTADUETO therapy, the drug should be promptly discontinued

- Macrovascular Outcomes

There have been no clinical studies establishing conclusive evidence of macrovascular risk reduction with linagliptin or metformin or any

Undesirable effects

There have been no therapeutic clinical trials conducted with Linagliptin and metformin tablets however bioequivalence of Metformin and linagliptin with co-administered linagliptin and metformin has been demonstrated. Serious adverse reactions including pancreatitis and hypersensitivity reactions have been reported. Hypoglycemia has been reported in combination with sulphonylurea (13.8%) and insulin (10.9%).

Special precautions for storage: Do not store above 30°C.

9. STATISTICAL ANALYSIS AND ASSESSMENT OF EFFICACY

9.1 General considerations

An independent (non-clinical centre), University-based group in Gothenburg will analyze and produce summary tables and data listings based on the common Statistical Analysis Plan (SAP). In the absence of any reason of code breaking mentioned before (see section 4.2), the blinding of the randomised treatments will be maintained until the database has been released for statistical analyses, after all participants have completed the 24 -month visit or have earlier discontinued study therapy.

No analyses of unmasked or between-group data will be performed before the database is closed or released, except for those highly confidential analyses performed by the Data Safety and Monitoring Committee (DSMC) to support the deliberations of the independent Steering Committee (SC) or in direct response to a recommendation by the SC.

Analysis of efficacy will be done both by intention-to treat (as randomised), and by protocol. Concerning primary analysis last visit available data will be used for these analyses.

The following statistical analyses will be performed: Final Analysis at the end of treatment at month 24 and observational follow-up analysis at year 3. Baseline analysis might also be used to refine the final sample size, in the case that prevalence of retinopathy be lower than expected. However the main treatment efficacy analysis only the month 24 analyses will be used.

9.2 Assessment of Efficacy:

There are two main purposes of the study 1) compare whether further glucose lowering by treatment with anti-diabetic therapies (intensive treatment) in the pre-diabetic state is beneficial in preventing diabetic complications, and 2) study whether any specific anti-diabetic agent of metformin, linagliptin, and the combination of linagliptin with metformin separately has a superior effect than life-style intervention in preventing diabetic complications in pre-diabetic individuals. This approach is similar to that used in the United Kingdom Prospective Diabetes Study (UKPDS) in type 2 diabetic individuals where both intensive therapy combining sulphonylureas and insulin therapy (two arms) were compared to conventional therapy and both therapies were also evaluated separately.

The primary analysis will be to compare complications in all three anti-diabetic therapies combined (metformin, linagliptin, and the combination of linagliptin with metformin), to complications in the life-style only arm to study whether further glucose lowering in the pre-diabetic state by anti-diabetic therapies reduces diabetic complications. However, the power calculation has been performed to make it possible to also answer whether any of the active therapies has a superior effect compared to life-style intervention. Since each of the 3 active pharmacologic arms separately is smaller than the 3 pharmacologic arms of active treatments combined, enough power will also be obtained for comparing the 3 pharmacologic groups combined with life-style intervention.

In previous studies of intensive glycaemic control in patients with type 2 diabetes, it cannot be concluded that any greater effects have been seen in preventing retinopathy, microalbuminuria or neuropathy. It should also be noticed that consistent effects in preventing microvascular complications have been difficult to prove when

lowering glucose levels close to normal even in very large studies. To obtain an as high power as possible these 3 events will therefore be combined in the primary analysis in a composite endpoint. Further on, the level of the Early Treatment Diabetic Retinopathy Study Scale (ETDRS), level of albumin excretion and neuropathy marker (sweat function) will be used, i.e. as continuous scales instead of a certain cut-off level which will also increase the power. The outcome in the primary analysis will hence be a composite outcome of retinopathy, albuminuria and neuropathy (here referred to as any microvascular complication) to obtain as high power as possible using a linear combination of the score of the Early Treatment Diabetic Retinopathy Study Scale (ETDRS), the level of urinary albumin to creatinine ratio and sweat function scale (SUDOSCAN). Weights of these 3 outcome measurements will be defined in the SAP and estimated from other studies. Analysis will be performed using Mann-Whitney U-test (two-sided, significance level 0.05) or alternatively analysis of covariance (ANCOVA) in case adjustment for baseline variables is required between the 3 active arms combined and the lifestyle arm. The same statistical analysis will be used when comparing each arm separately to the life-style arm. The analyses will be performed on the full analyses set (ITT-population) with adjustments for baseline variables significantly differing between the groups and significantly related to the outcome variable.

The primary comparison within the study report will be:

- Life-style + 3 active arms combined vs Life-style group

The secondary comparisons within the study report will be:

- Life-style + metformin group vs life-style group
- Life-style + linagliptin group vs life-style group
- Life-style + combination of metformin and linagliptin group vs life-style group
- Life-style + metformin group vs life-style + linagliptin group
- Life-style + metformin group vs life-style + combination of metformin and linagliptin group
- Life-style + linagliptin group vs life-style + combination of metformin and linagliptin group

The primary and selected secondary efficacy analyses will be performed on the ITT population in the following order:

Primary analysis

1) Compare any microvascular complication by intensive antidiabetic treatment (3 active arms combined) to life-style intervention alone (life-style arm).

Secondary analyses

2) Compare the score of the ETDRS scale by intensive antidiabetic treatment (3 active arms combined) to life-style intervention.

3) Compare the level of albumin/creatinine ratio by intensive antidiabetic treatment (3 active arms combined) to life-style intervention.

4) Compare sweats function score by intensive antidiabetic treatment (3 active arms combined) to that in life-style intervention.

5) Compare any microvascular complication separately for each active therapy to life-style intervention.

6) In the following order separately compare the score of the ETDRS scale, level of albumin creatinine ratio, and sweat function score for each active diabetic agent separately to life-style intervention.

7) Treatment with linagliptin and the combination of linagliptin with metformin will both be compared regarding complications to the metformin group in separate analyses. Analyses will be performed in the following order of events: any microvascular complication, ETDRS score, albumin-creatinine ratio and neural conduction velocity.

For the numbered analyses above the theory of sequentially multiple test procedures (37) will be applied. If a test gives significant result on 0.05 significant level the total test mass will be transferred to the following number in the test sequence until a non-significant result is achieved.

All other endpoints will be analysed without any specific order: cardiovascular complications, peripheral vascular disease, microvascular endothelial function, intima media thickness of the common carotid artery, biomarkers of inflammation, biomarkers of microvascular damage, biomarkers of early DR, biomarkers of vascular damage, fatty liver index, sleep apnea, quality of life, hypoglycaemia, mini-mental state examination questionnaire for cognitive function, insulin secretion rate and glucose sensitivity.

For comparison between two groups, continuous variables will be analysed unadjusted by Mann-Whitney U-test , ordered categorical variables by Mantel-Haenszel Chi-square test, non-ordered categorical variables by Chi-2 test, dichotomous variables by Fisher's exact test and time to event by Logrank test. All primary and secondary efficacy analyses will be adjusted for variables significantly differing between the groups and significantly related to the outcome variable. In this case, the difference between the groups will be tested using logistic regression for dichotomous variables and ANCOVA, for continuous variables. All significance tests will be two-sided and conducted at the 0.05 significance level.

Calculation of 24-months and 36-months cumulative incidence for each separate dichotomous event will be calculated for each study arm and for combined 3 treatment arm. Relative and Absolute Risk Reductions (RRR and ARR) will be calculated.

Further statistical analyses of the treatment effect on time to event will be analyzed using Poisson regression models adjusted for baseline covariates. These models allow analysis of effects of time in study and interaction between treatments and time in study.

Further exploratory statistical analyses of the remaining treatment effect on time to event when adjusted for baseline covariates and other time dependent covariates, such as HbA1c and plasma glucose levels over time, will also be analyzed using Poisson regression. The updated values for covariates will be determined by calculating a function of specified values (e.g. HbA1c), including all values measured from baseline to the time closest to the primary endpoints assessment.

In addition, exploratory longitudinal analyses of point prevalence will be conducted. Most such analyses will be conducted using multivariate methods e.g. the method of generalized estimating equations, and longitudinal mixed-effects growth-curve models.

Descriptive summary statistics for continuous variables will include the number of observations (N), mean, standard deviation (SD), median and range.

Descriptive summary statistics for categorical variables will include frequency counts and percentages [N (%)]. Unless stated otherwise in the mock tables, the denominator for percentage calculations will be the number of patients in the analysis population and treatment group.

Demographics, Baseline characteristics and all efficacy variables will be analyzed using the ITT and PP population and the ITT analysis will be the main analysis. Demographics, Baseline characteristics and all safety variables will be analyzed using the safety population.

All statistical analyses of secondary, tertiary endpoints, safety endpoints and other variables will be specified in detail in the main SAP. The SAP will also list all tables, figures and listings to be included in the Statistical report and in the Clinical Study Report. The SAP will be written by the Statistical methodology and analysis team and will be reviewed by the Steering Committee. The SAP will be signed before the clean file meeting and the locking of the database.

A Tables Manual will also be written and reviewed with the exact layout of all tables, figures and listings given in the main SAP.

Cost-effectiveness and cost-utility analysis in terms of use of therapies, incidence of complications, morbidity and associated effect on costs and health related quality of life will be specified in detail in a separate SAP.
Insulin sensitivity and beta-cell function will be determined by modelling analysis OGTTs, according to validated methodologies (36) The modelling results will include the glucose tolerance parameters, an insulin sensitivity index (37) and several beta-cell function parameters representing various aspects of the beta-cell response ((38). These parameters include in particular: 1) fasting insulin secretion and total insulin secretion during the OGTT; 2) beta-cell glucose sensitivity, a parameter quantifying the ability of the beta cell to respond to the glucose changes; 3) rate sensitivity, a parameter quantifying the early insulin secretion response (a marker of first phase secretion); 4) the potentiation factor, which quantifies the time-dependent amplification of insulin secretion.

The details of the analysis of insulin sensitivity and beta-cell function will be given in a separate SAP.

10. ASSESSMENT OF SAFETY

An independent external Data Safety and Monitoring Committee (DSMC) will be constituted for the trial to oversee safety and perform ongoing safety surveillance at pre-defined time points as well as ad-hoc. The DSMC will evaluate all relevant safety information received in DSMC data packages and will have access to un-blinded data.

The DSMC is composed of permanent members who cover relevant specialties (cardiology, endocrinology, gastroenterology and statistics).

Following each DSMC meeting, the DSMC will issue a recommendation statement as to whether the trial may continue, should be modified, or terminated as necessary. Clear evidence of net harm with respect to total mortality, cancer, hospitalisations or other variables identified by the DSMC based on emerging data from this or other trials, that is consistent over time and across subgroups would justify a recommendation to stop the trial early.

The DSMC members must disclose any potential conflicts of interest and must be independent of clinical centres. They shall be free of financial interest that could be substantially affected by the outcome of this trial and no participating Investigator's should be members of the DSMC. The DSMC works in accordance with written guidelines, tasks and responsibilities, schedule for review of data and pre-planned decisions points, specific data to be reviewed, work processes, pre-planned statistical analysis and the level of access to un-blinded data.

DSMC meetings will be held as a minimum every 6 months or more frequently if requested by the Steering Committee based on the outcome of DSMC data reviews. An independent statistician will prepare data packages

including accumulated safety data before each DSMC. All data can be presented un-blinded. DSMC recommendations will be submitted on an expedited basis to local Health Authorities and to IECs/IRBs according to local requirements.

All AES and SAEs will be assessed and classified by the Data Safety Monitoring Committee (DSMC) composed of cardiologists, neurologists, diabetologists, and general clinicians who will be masked to treatment assignment. These AEs and SAEs will then be encoded using the Medical Dictionary for Regulatory Activities (MedDRA, Version 12.1) system. This is a medically validated terminology database developed by the International Conference on Harmonization. Within the MedDRA, AEs are grouped by SOC, eg, “cardiac disorders” or “gastrointestinal disorders”. Within an SOC, specific AEs are identified by PT. For instance, the PTs included in the analysis of edema-related AEs, one of the most common AEs with some of blood glucose lowering drugs in the trial, are allergic edema, generalized edema, local swelling, localized edema, edema, peripheral edema, pitting edema, skin edema, skin swelling, and swelling.

All AEs and SAEs should be collected, assessed and recorded by the local investigator as to the severity, possible relationship to the study medication and onset.. This will include laboratory abnormalities if considered an AE by the investigator. All AEs and SAEs shall be tabulated in the statistical report as total, by severity AEs and relationship to the study medication. The collection, CRF-handling (cleaning) and the coding of the AEs and SAE's will be data management activity under WP9.

10.1 Definitions

An AE or Adverse Event is:

Any untoward medical occurrence in a patient or clinical investigation participants administered a medicinal product, which does not necessarily have to have a causal relationship with this treatment (the study medication).
An AE can therefore be any unfavourable and unintended sign (including an abnormal laboratory finding), symptom or disease temporally associated with the use of the study medication/procedure, whether or not considered related to the study medication.

An AR or Adverse Reaction is:

All untoward and unintended responses to a medicinal product related to any dose.
The phrase "responses to a medicinal product" means that a causal relationship between a study medication and an AE is at least a reasonable possibility, i.e., the relationship cannot be ruled out.
All cases judged by either the reporting medically qualified professional or the sponsor as having a reasonable suspected causal relationship to the study medication/procedure qualify as adverse reactions.

A SAE or Serious Adverse Event is:

- *Cardiovascular disease*: death secondary to cardiovascular disease or any sudden death judged not to be caused by hypoglycaemia or other known reason, acute myocardial infarction (MI), silent MI appearing as a major new Q-wave abnormality on a routine ECG, initiation of thrombolytic therapy for suspected MI, coronary artery disease (CAD) requiring bypass surgery or angioplasty, or CAD confirmed by angiography or by a combination of angina and ischemia documented with non-invasive testing.
- *Cerebrovascular disease*: stroke or transient ischemic attack confirmed by angiography or non-invasive testing.
- *Peripheral vascular disease* (PVD): surgical amputation of a lower extremity necessitated by vascular disease, arterial vascular events requiring bypass or angioplasty, claudication with exercise testing or angiographic evidence of vascular disease, or an ankle-to-arm blood pressure ratio <0.8 or >1.4.

• *Confirmed hypoglycaemia* defined as symptoms suggestive of low blood glucose confirmed by self-monitored blood **glucose measurement, <3.1 mmol/L plasma glucose equivalent.**
• *Severe hypoglycaemia*: events that require assistance from another individual, including episodes of seizure and/or coma.

To ensure no confusion or misunderstanding of the difference between the terms "serious" and "severe", which are not synonymous, the following note of clarification is provided:
The term "severe" is often used to describe the intensity (severity) of a specific event (as in mild, moderate, or severe myocardial infarction); the event itself, however, may be of relatively minor medical significance (such as severe headache). This is not the same as "serious," which is based on patient/event outcome or action criteria usually associated with events that pose a threat to a participant's life or functioning as defined in the bullet points above. Seriousness (not severity) serves as a guide for defining regulatory reporting obligations.

A SAR or Serious Adverse Reaction is:
An adverse event (expected or unexpected) that is both serious and, in the opinion of the reporting investigator, believed with reasonable probability to be due to one of the study treatments, based on the information provided.

A SUSAR or Suspected Unexpected Serious Adverse Reaction is:
A serious adverse reaction, the nature or severity of which is not consistent with the applicable product information (e.g. Investigator's Brochure for an unapproved investigational product or summary of product characteristics for an approved product).

10.2 Causality and Expectedness

The relationship of each adverse event to the trial medication must be determined by a medically qualified individual at each clinical centre according to the following definitions:
Related: *The adverse event follows a reasonable temporal sequence from trial medication administration. It cannot reasonably be attributed to any other cause.*
Not Related: *The adverse event is probably produced by the participant's clinical state or by other modes of therapy administered to the participant.*

10.3 Procedures for Recording Adverse Events

All AEs occurring during the study until the 36 month after the initiation of the therapy observed by the *investigator or reported by the participant, whether or not attributed to study medication, will be recorded on the CRF.*

A specific form to report will be distributed with clear instruction to report any SAE immediately to the coordination center by completing the SAE Report Form available in OpenClinica and sent it urgently by mail/fax to: sae@evidem.com / Fax: +34914025066

The following information will be recorded: description, date of onset and end date, severity, assessment of relatedness to study medication, other suspect drug or device and action taken. Follow-up information should be provided as necessary.

AEs considered related to the study medication as judged by a medically qualified investigator at the local centre will be communicated to the central coordination (sponsor) and will be followed until resolution or the event is

considered stable. All related AEs that result in a participant's withdrawal from the study or are present at the end of the study, should be followed up until a satisfactory resolution occurs.

It will be left to the investigator's clinical judgment whether or not an AE is of sufficient severity to require the participant's removal from treatment. A participant may also voluntarily withdraw from treatment due to what he or she perceives as an intolerable AE. If either of these occurs, the participant must undergo an end of study assessment and be given appropriate care under medical supervision until symptoms cease or the condition becomes stable.

Any pregnancy occurring during the clinical study and the outcome of the pregnancy should be recorded and followed up for congenital abnormality or birth defect.

10.4 Reporting Procedures for Serious Adverse Events

All SAEs must be reported to the Sponsor or designated organization within one working day of discovery or notification of the event. The Sponsor or designated organization will perform an initial check of the report, request any additional information. All SAE information must be recorded on an SAE forms and faxed to the Sponsor or designated organization. Additional information received for a case (follow-up or corrections to the original case) need to be detailed on a new SAE form and faxed to the Sponsor or designated organization.

It may be appropriate that some SAEs do not require immediate reporting but this must be justified. Justification might be determined, for example, by admission to hospital, or prolongation of hospitalization, where this is to be expected in the underlying disease or condition.

10.5 Type and Duration of Follow-Up of Subjects after Adverse Events

Describe how AEs will be followed until resolved or considered stable. Specify procedures for reporting and follow-up of AEs that are consistent with the Schedule of Events. Include duration of follow-up after appearance of AEs (e.g., 1 week, 2 months).

10.6 Safety analysis

- For AEs, SAEs, treatment related AEs and SAEs, hypoglycaemic AEs and SAEs, medical events of special interests AEs and SAEs, major cardiovascular AEs and SAEs, discontinuations due to AEs and deaths, incidence will be calculated as number of patients with an event divided by the number of patients in the treatment group. They will be presented by System Organ Class (SOC) and Preferred Term (PT) enligt MedDRA dictionary.
- The incidence of single or recurrent events, such as hypoglycemia, will be summarized as a crude rate. Such rates will be presented as the number of events per 100 patient-years based on the ratio of the observed number of events to the total patient-years of exposure. The standard error for such rates will be computed allowing for over dispersion.

11. SAMPLE SIZE CALCULATION

11.1 Power calculation

The main comparison of this study was originally estimated to be between two groups of the size 750 and 2250 with respect to a score, which is a linear combination of three scores. We estimated to achieve the power 80% at

the significance level 0.05, two-sided test, provided that the difference exceeded 0.12 times the standard deviation of the score. We have no experience of the distribution of the score, but to get an idea of the difference we consider another variable, HbA1c. In a population we earlier found that the standard deviation was 1.55.

Thus $0.12 \cdot 1.55 = 0.18 \approx 0.2$ and the sample size give the power 80% to assess a difference between the mean 7.0 versus 6.8.

However, since the original calculation we have gained experience from larger patient materials and several clinical trials that the standard deviation in HbA1c is generally considerably lower than 1.55%. In a recent study comprising more than 90% of individuals with type 2 diabetes in Sweden, the SD of HbA1c was 1.3%. In randomized placebo-controlled trials that we have recently performed we checked the SD of HbA1c in earlier similar trials and found that an SD of 1.2% was often used, considered to be generally conservative. In a recent trial we performed the SD of HbA1c was just below 1% (2). Hence, an SD of HbA1c of 1.4% should be viewed as conservative and therefore used in the current complementary calculations.

Assuming the difference in the primary variable between the groups (proportion 1:3) of 0.2 and a standard deviation of 1.40e, we estimate that 2000 evaluable individuals are needed to obtain a power of 79% by using two-sided t-test and the significance level of 0.05. If there are expected to be 10% individuals discontinuing the trial which has been the case in earlier similar randomized trials (4), approximately 2200 patients would be needed to obtain the same power of 79%. If there are 10% of patients among 2000 patients discontinuing the trial (1800 continuing, 200 discontinuing) the corresponding power would instead be 75%.

Our view from the above performed estimations is that 2000 patients at a minimum should be included in the ePREDICE trial. The calculation was performed by using procedure PROC POWER in SAS.

Events versus a continuous variable

When studying the effect of a treatment compared with another one we can either use the event of getting the diagnosis during the study period or we can perform the comparison between the treatments with respect to the change or the final value of the continuous variable. The latter type of comparison turns in many situations out to be much more efficient than the comparison of the incidence of events. A basic scientific principle tells us that we should apply the most powerful type of comparison even if it is of interest (as a later step) to elucidate the effect in terms of hazard functions of events.

Only at the end of the study X is observed. If $X>1.28$ (the cut off limit) an event has occurred. The probability of event is 0.10 for control group and 0.0604 for the new treatment group. In order to achieve the power 80% at the significance level 0.05, two-tailed test, $2 \cdot 750$ patients are needed (Fisher's exact test). If we instead compare the two groups with respect to X only $2 \cdot 217$ patients are needed, t-test for two samples, and $2 \cdot 226$ patients are needed if Mann-Whitney's test is applied. Below also other cut off limits are considered.

Table2. Sample size calculations for different continuous variable cut-offs (power 80%).

Cut Off	Probability of values above the limit		Number needed for the power 80%
	Control	New treatments	
0.00	0.5000	0.3936	2 · 351
1.00	0.1587	0.1020	2 · 562
1.28	0.10	0.0604	2 · 750
1.50	0.0668	0.03837	2 · 980

In the present study the power calculations were done for incidences of events but in order to achieve the maximum efficiency the primary analyses will be performed by use of the corresponding continuous variables.

The use of continuous variables in this case is not only more powerful due to the reasons shown above but also for another reason. The development of a complication to IGT or to diabetes, but especially to IGT, will probably take a long time and the change of the corresponding continuous variable will be a much earlier sign and thus the difference in power is probably even larger than shown above.

As a primary aim we will compare a linear combination of a retinopathy scale, U-albumin and a score of neuropathy. The coefficients of the linear combination will be determined on the basis of information from other materials available before the analysis of this study so the expected value of the power determining quantity will be as large as possible. One of the coefficients could without loss of efficiency be put to 1, so there are two of them to be determined. Because other materials than that from the present study are used, no bias is introduced by the optimization. As further aims we will analyze the risk of the different events depending on treatment and other variables.

11.2 Adjustment of sample size and statistical power

When ePREDICE trial was originally designed, no empirical data on the effect of treatment on microvascular complications in prediabetes existed. Therefore original sample size was only based on theoretical statistical assumptions. By July 2017 when the recruitment, follow-up and primary outcome data collection were sufficient,. we followed the statistical recommendations and evidence recently published on adaptative trial design to allow for interim sample size reassessment (Thorlund K. BMJ 2018; doi:10.1136/bmj.k698). Therefore the sample size and power calculation were revised by an independent external statistician, under supervision of Prof. Marcus Lind, leader of WP8 (see D8.4 for more details).

The database for the revised power calculation contained 173 participants with both baseline and one-year follow-up SUDOSCAN data, and information on group of intervention assigned.

The interim analysis of baseline distribution (cross-sectional analysis) and 1-year changes (paired t-test analysis) of SUDOSCAN parameters in the first 149 patients randomized in ePREDICE was performed for this purpose. The statistical software used for the analysis was SAS version 9.4.

No statistical differences in socio-demographic, clinical and main outcome variables was observed between two treatment groups at baseline

For sample size analysis, we selected the variation of CAN Risk Score between baseline and one year follow-up (mean ± SD, median and Q1-Q3 range).
After 1-year follow-up, absolute mean difference in CAN risk score was 3.6 units significantly lower in Group 2 (lifestyle & pharmacological intervention) than in Group 1 (only lifestyle intervention).
Based on the above new empirical data on the incidence of neuropathy, revised sample size calculations was performed with the validated academic public statistical software GRANMO, version 7.0 available at: *https://www.imim.cat/ofertadeserveis/software-public/granmo/* using the module «repeated measurements» (paired t-test for means).

We calculated two sample sizes for two different statistical scenarios, assuming the following conditions:

a) Unfavourable scenario: minimum absolute difference between groups 1.8 units (half mean difference of observed in the sample), common standard deviation of 12.1 in the whole population (higher than observed), follow-up dropout rate 0.25 (maximum expected 0.20), correlation coefficient between first and second SUDOSCAN measurements 0.80 (0.83 previously reported for feet3), and Group 1 size/Group 2 size ratio of 3.

   The calculation with these assumptions gives the total sample size for all 4 randomization groups together N= 1012 individuals

Accepting an alpha risk of 0.05 and a beta risk of 0.2 in a two-sided test, 253 participants are necessary in Group 1 (lifestyle + placebo) and 759 in Group 2 ((lifestyle + any drug intervention) to recognize as statistically significant a difference greater than or equal to 1.8 units. The common standard deviation is assume 12.1 and the correlation coefficient between the initial and final measurement as 0.8. We foreseen a dropout rate of 25%.

b) Realistic (conservative) scenario: minimum absolute difference between groups 1.8 units (half mean difference of observed in the sample), common standard deviation of 11.4 in the whole population, follow-up drop-out rate 0.25 (maximum expected 0.20), correlation coefficient between first and second SUDOSCAN measurements .80 (0.83 previously reported for feet3), and Group 1 size/Group 2 size ratio of 3.

   The calculation with these assumptions gives the total sample size for all 4 randomization groups together: N= 896 individuals

Accepting an alpha risk of 0.05 and a beta risk of 0.2 in a two-sided test, 224 participants are necessary in Group 1 (lifestyle + placebo) and 672 in Group 2 (lifestyle + any drug intervention) to recognize as statistically significant a difference greater than or equal to 1.8 units. The common standard deviation is assume 11.4 and the correlation coefficient between the initial and final measurement as 0.8. It has been anticipated a dropout rate of 25%.

Accepting an alpha risk of 0.05 in a two-sided test with 224 participants in Group 1 and 672 in Group 2, the statistical power was 82% to recognize as statistically significant a difference of means (1-year CAN Risk Score of 34.6 in Group 1 and 31.5 in group 2).Power calculation has been also performed with the statistical software GRANMO, version 7.0.

The previous sample size of 2.000 patients was based on the theoretical calculations, with no empirical evidence (no data published). Power calculated was 75.4%.
Basic statistical principles recommend the most powerful type of comparison should be apply for sample size calculation. Continuous variables are more powerful than categorized clinical variables. Also the development of clinical events take a long time, while the change in the corresponding continuous variable will be a much earlier sign of efficacy.

SUDOSCAN parameters are continuous variables, which are more efficient than the comparison of the incidence events. The use of SUDOSCAN as a continuous variable allows reducing sample size by half while maintaining the statistical power of the study in 82%.
In conclusion, a minimum sample size of 896 participants would be sufficient to detect significant differences in the risk of neuropathy between treatment groups in ePREDICE trial.

12. DIRECT ACCESS TO SOURCE DATA/DOCUMENTS

Direct access will be granted to authorize representatives from the sponsor, host institution and the regulatory authorities to permit trial-related monitoring, audits and inspections. See description of *Data protection issues* on section 6.5 of this document.

13. QUALITY CONTROL AND QUALITY ASSURANCE

Quality control

Quality control procedures in the ePREDICE study include those in place internally in all the laboratories and reading centres as well as those implemented as part of ePREDICE data collection.
The local clinic procedures that require training and certification include the performance of Endothelial function by Endo-PAT, sudomotor function with SUDOSCAN, ECG recording, carotid ultrasound, and retinal fundus photographs. All blood and urine tests undergo repeated assessments in a subsample for analytic precision by assays of split-duplicate samples in the central laboratory. Split-duplicate analysis is also used to monitor grading of the fundus photographs, ECGs and IMT measurements.

Data handling and monitoring

Data management will follow specific procedures using the web-based, open resource computerized system for clinical research OpenClinica Enterprise (https://community.openclinica.com) which permits:

- Create Study.
- Create CRF.
- Create Event Definitions, based on OIDs, type, category, date
- Create Subject Group Classes,
- Create Rules to perform advanced validation based on Object Identifiers (OIDs) that identify various entities (Studies, Sites, Events, etc..)
- Create Sites
- Assign Users, types and roles

Online monitoring and data management

- Source data verification (SDV)

- Study Audit Logs
- Rules for Cross Field and Cross form edit checks and advanced validations.
- Groups:
- CRFs: view all the Case Report Forms (CRFs) that are available for use in the study or sites

Data entry

The *OpenClinica* software will be used for data entry at each local centre. This program has a verification module that ensures the quality of data entry. The printed data will be stored locally.

All subjects included in the study will be allocated a personal ID number created with an algorithm based on the centre of origin, and a pre-established ID number. The ID will appear on each table of the study. The different data tables to be included in the study are the following: baseline data, matching the CRF data of visit 0, table of baseline data, tables for each follow-up visit and monitoring and events tables. Data management is the responsibility of the CRO: EVIDEM CONSULTORES SL .

Appropriate measures such as encryption of data files will be used to assure confidentiality of subject data when it is transmitted over open networks. The subject and the biological material obtained from the subject will be identified by subject number, trial site and trial identification number. Appropriate measures such as encryption or deletion will be enforced to protect the identity of human subjects in all presentations and publications as required by local/regional/national requirements.

Laboratory data will be transferred electronically from the central laboratory performing clinical analyses. In cases where laboratory data is transferred via non-secure electronic networks; data will be encrypted during transfer. The transfers will be done according to agreed timelines. The final database will be transferred to the coordination centre and the Statistical Analysis Centre.

14. ETHIC, DEONTOLOGICAL AND REGULATORY CONSIDERATIONS

General ethic issues

The study will strictly follow the regulations adopted by the World Medical Association Declaration of Helsinki, and subsequent amended included the latest in the 59th WMA General Assembly, Seoul, October 2008 (parts A, B and C); as well as local/national regulations regarding ethical committees and data protection regulations. Each participating centre will be responsible for obtaining all relevant approvals from ethical committees, data protection agencies etc. No centre can start data collection until a copy of all local approval have been sent to the principal investigator and sponsor of the study (EVIDEM). An individual approval is needed for each part of the study. Informed consent from each individual will be obtained by the participating centre. Standardised patient history, consent and investigation forms are used. No payment/salary reimbursement will be given in any part of the study, but participants in the intervention study can be offered reimbursement of their travel costs by specification due to the repeated visits, if necessary. The research in this study fulfils legal and ethical requirements of the member states and ICPC states where it is carried out. This project is designed for adult people regardless of gender, cultural, religious or ethnic background. The sensitive data to be submitted to the central data base will be anonymous, no personal identifier will be sent outside the local participating centres.

Benefits and burden of the experiments and safety of study subjects

In addition to randomised treatment, namely the combination of linagliptin with metformin, linagliptin or metformin, all subjects will be enrolled in the intensive lifestyle intervention programme. Investigators will be encouraged to manage subject’s glucose control (in the rescue therapy) and other risk factors (blood pressure, lipids) according to current evidence-based standards. Current treatment guidelines will be provided to investigator's in a written form throughout the trial. Furthermore, subjects may benefit from the weight stabilising effects demonstrated for lifestyle intervention. Another potential benefit of participating in the trial is that the trial flow will ensure management at regular intervals (e.g., HbA1c, BP, lipids, renal status etc.), which should further facilitate individualized management of subjects.

The trial drug products may be associated with adverse events, but relevant precautions have been implemented in the design and planned conduct of the trial in order to minimise the risks and inconveniences of participating in the trial. These precautions include thorough information regarding the correct administration of the trial products and gradual dose adjustment to mitigate the gastrointestinal side effects. Furthermore, subjects will be fully informed about possible adverse event profile of the trial products and potential other inconveniences and will be instructed to contact the investigator in case of any concerns regarding the trial participation. All specific safety areas will be subject to ongoing monitoring of un-blinded data by an independent DSMB. See also the exclusion criteria, the safety assessment and the analysis sections, for specific safety areas of interest (related with the participation of subjects in this proposal.

Study significance and benefit to local communities

Potential benefits of the proposed study are:

- Broad implementation of a combined (drug+lifestyle) treatment strategy has the potential to reach more people with greater efficiency than traditional care based on single therapy. Combining several drugs with lifestyle intervention is likely to be more effective and have fewer side effects than high-dose therapy with a single drug o several drugs.
- The outcome of this study will contribute to our understanding of the pathophysiology of early prevention of diabetes complications, and guide the design of future clinical trials.
- The study will help build an initial network of physicians committed to both research and prevention of Diabetes complications and CVD in Europe and Australia.

Other beneficial considerations are:

- There are few reports on the effects (benefits) of the different new families of glucose lowering drugs (DPP-IV inhibitors); how these new drugs would work in pre-diabetic patients is yet to be understood.

The countries where the clinical research (randomized clinical trial) will be undertaken are: Austria, Bulgaria, Greece, Italy (canceled), Lithuania (canceled), Poland, Serbia, Spain, Turkey and Australia (canceled)

The legal framework applicable to this protocol is that of the European Medicines Agency of (EMA) for the EU state members listed above, and the medicine agencies for those participating centres in non-EU countries (Serbia and Turkey). Regulatory authorities will receive the clinical trial application, substantial/non-substantial amendments to the protocol, reports on SAEs, and the CTR according to national requirements.

Prior to commencement of the trial the protocol, any amendments, subject information/informed consent form, any other written information to be provided to the subject, subject recruitment procedures (incl. advertisement), if any, IB, or package insert linagliptin, metformin and the combination of linagliptin with metformin, available safety information, information about payments and compensation available to subjects if not mentioned in the subject information, the investigator's current curriculum vitae (CV) and/or other documentation evidencing qualifications, and other documents as required by the local IRB/IEC should be submitted. The submission letter should clearly identify the trial identification number, version, EudraCT no., title and/or date of the documents that have been submitted to the IRB/IEC. Written approval/favourable opinion must be obtained from IRB/IEC prior to commencement of the trial.

During the trial, the investigator must promptly report the following to the IRB/IEC, in accordance with local requirements: updates to IB, unexpected SAEs where a causal relationship cannot be ruled out, substantial amendments to the protocol, non-substantial protocol amendments according to local requirements, deviations to the protocol implemented to eliminate immediate hazards to the subjects, new information that may affect adversely the safety of the subjects or the conduct of the trial (including new risk/benefit analysis in case it will have an impact on the planned follow-up of the subjects), annually written summaries of the trial status and other documents as required by the local IRB/IEC.

Substantial protocol amendments must not be implemented before approval/favourable opinion, unless necessary to eliminate immediate hazards to the subjects.The investigator must maintain an accurate and complete record of all submissions made to the IRB/IEC. The records should be filed in the investigator's trial file and copies must be sent to the CC.

The only persons with access to the participant´s clinical record are the participant´s physician and nurse. Identification of potential eligible participants will be done by local family physicians and nurses. No external personnel will perform any screening or check patient eligibility. All clinical visits, procedures and follow-up will be conducted by the local health personnel at each participating clinical centre. Neither the Coordinating Centre, nor the CRO personnel (monitors) will have ever access to patient´s information in clinical records.

All clinical recruitment centers should translate the protocol and the informed consent into their own language, and to submit them to the local ethical committee. Copies of approval from the local committees will be requested and collected by the coordinating centre (EVIDEM) and submitted to the EC before starting actual patient recruitment.

When submitting the application for scrutiny to the *compet*ent local/national ethical boards/bodies for authorization/opinions/notifications, detailed information must be provided on the informed consent procedures that will be implemented. Copies of examples of Informed Consent Forms and information Sheets must be included. These must be in language and terms understandable to the participants.

Participants must have the right:
- To know that participation is voluntary

- To ask questions and receive understandable answers before making a decision
- To know the degree of risk and burden involved in participation
- To know who will benefit from participation
- To know the procedures that will be implemented in the case of incidental findings
- To receive assurances that appropriate insurance cover is in place
- To know how their data will be collected, protected during the project and either destroyed or reused at the end of the research, if plan to reuse the data exist,
- Participants should be duly informed, and consented also for this further usage
- To know of any potential commercial exploitation of the research

The different local ethic committees approving the study protocol will perform the ethic monitoring at local centres. We will identify and invite an international, independent ethic expert to coordinate the local ethic monitoring program and produce the annual periodic report for the EC. The annual periodic report is now included as a deliverable in WP1.

All partners and countries participating in ePREDICE will follow the EU directives:

- Directive 2001/20/EC OF the European Parliament and of the Council of 4 April 2001 on the approximation of the laws, regulations and administrative provisions of the Member States relating to the implementation of good clinical practice in the conduct of clinical trials on medicinal products for human use.
- Commission Directive 2005/28/EC of 8 April 2005 laying down principles and detailed guidelines for good clinical practice as regards investigational medicinal products for human use, as well as the requirements for authorization of the manufacturing or importation of such products.

Ethical standards in ePREDICE will follow the guidelines:

Detailed guidance on the request to the competent authorities for authorisation of a clinical trial on a medicinal product for human use, the notification of substantial amendments and the declaration of the end of the trial (CT-1) (2010/C 82/01)
Good Clinical Practice Guideline, under the Legislative basis Directive 75/318/EEC as amended ICH E6: *Good Clinical Practice: Consolidated guideline*, CPMP/ICH/135/95

15. REFERENCES

Evidence-based state-of-the-art (references by theme).

- Definition, classification and clinical diagnosis of hyperglycaemia and diabetes:
  1. Definition and diagnosis of diabetes mellitus and intermediate hyperglycemia: report of a WHO/IDF consultation. Geneva 2006.
- Diabetic microvascular vs macrovascular disease complications
  2. Orasanu G, Plutzky J. The Continuum of Diabetic Vascular Disease: From Macro- to Micro-J Am Coll Cardiol. 2009; 53(5 Suppl): S35–S42
- Hyperglycaemia and microvascular disease progression
  3. Balkau B, Hu G, Qiao Q, Tuomilehto J, Borch-Johnsen K, Pyorala K; DECODE Study Group; European Diabetes Epidemiology Group. Prediction of the risk of cardiovascular mortality using a score that includes glucose as a risk factor. The DECODE Study. Diabetologia 2004;47:2118-28
  4. Diabetes Prevention Program Research Group. The prevalence of retinopathy in impaired glucose tolerance and recent-onset diabetes in the Diabetes Prevention Program. Diabet Med 2007; 24:137–144
  5. Wong TY, Liew G, Tapp RJ, et al. Relation between fasting glucose and retinopathy for diagnosis of diabetes: three population-based cross-sectional studies. Lancet 2008;371:736–43
- Molecular bases of microvascular disease
  6. Brownlee M. The pathobiology of diabetic complications: a unifying mechanism. Diabetes 2005;54:1615–25
  7. Quagliaro L, Piconi L, Assaloni RG, et al. Intermittent high glucose enhances ICAM-1, VCAM-1 and E-selectin expression in human umbilical vein endothelial cells in culture: the distinct role of proteinkinase C and mitochondrial superoxide production. Atherosclerosis 2005;183:259–7.
- Lifestyle interventions and prevention of microvascular complications
  8. Tuomilehto J, Lindström J, Eriksson JG, Valle TT, Hämäläinen H, Ilanne-Parikka P, Keinänen-Kiukaanniemi S, Laakso M, Louheranta A, Rastas M, Salminen V, Uusitupa M; Finnish Diabetes Prevention Study Group. Prevention of type 2 diabetes mellitus by changes in lifestyle among subjects with impaired glucose tolerance. N Engl J Med. 2001 May 3;344(18):1343-50
  9. The Diabetes Prevention Program Research Group. Reduction in the incidence of type 2 diabetes with lifestyle intervention or metformin. N Engl J Med 2002;346(6):393-403
- Pharmacological treatment for prevention of microvascular complications
  10. Q. Gong & E. W. Gregg & J. Wang & Y. An & P. Zhang &W. Yang & H. Li & H. Li & Y. Jiang & Y. Shuai & B. Zhang & J. Zhang & R. B. Gerzoff & G. Roglic & Y. Hu & G. Li & P. H. Bennett Long-term effects of a randomised trial of a 6-year lifestyle intervention in impaired glucose tolerance on diabetes-related microvascular complications: the China Da Qing Diabetes Prevention Outcome Study. Diabetologia 2011; 54:300–307.
  11. Holman RH, Paul SK, M. Bethel MA, Matthews DR, and Neil HAV. 10-Year Follow-up of Intensive Glucose Control in Type 2 Diabetes N Engl J Med 2008;359:1577-89.
  12. Heller SR on behalf of the ADVANCE Collaborative Group. A Summary of the ADVANCE Trial. Diabetes Care 2009; 32(Suppl 2): S357-61.
  13. ACCORD Study Group; ACCORD Eye Study Group, Chew EY, Ambrosius WT, Davis MD, Danis RP, Gangaputra S, Greven CM, Hubbard L, Esser BA, Lovato JF, Perdue LH, Goff DC Jr, Cushman WC, Ginsberg HN, Elam MB, Genuth S, Gerstein HC, Schubart U, Fine LJ. Effects of medical therapies on retinopathy progression in type 2 diabetes. N Engl J Med. 2010;363:233-44.
  14. NAVIGATOR Study Group, Holman RR, Haffner SM, McMurray JJ, Bethel MA, Holzhauer B, Hua TA, Belenkov Y, Boolell M, Buse JB, Buckley BM, Chacra AR, Chiang FT, Charbonnel B, Chow CC, Davies MJ, Deedwania P, Diem P, Einhorn D, Fonseca V, Fulcher GR, Gaciong Z, Gaztambide S, Giles T, Horton E, Ilkova H, Jenssen T, Kahn SE, Krum H, Laakso M, Leiter LA, Levitt NS, Mareev V, Martinez F, Masson C, Mazzone T, Meaney E, Nesto R, Pan C, Prager R, Raptis SA, Rutten GE, Sandstroem H, Schaper F, Scheen A, Schmitz O, Sinay I, Soska V, Stender S, Tamás G, Tognoni

G, Tuomilehto J, Villamil AS, Vozár J, Califf RM. Effect of nateglinide on the incidence of diabetes and cardiovascular events. N Engl J Med;362:1463-76.

15. American Diabetes Association. Standards of medical care in diabetes-2011. Diabetes Care. 2011 Jan;34 Suppl 1:S11-61.

- Novel pharmacological treatments for hyperglycaemia

16. G. Schernthaner , A. H. Barnett, D. J. Betteridge, R. Carmena , A. Ceriello, B. Charbonnel , M. Hanefeld, R. Lehmann , M. T. Malecki, R. Nesto, V. Pirags, A. Scheen, J. Seufert, A. Sjohölm, A. Tsatsoulis, R. DeFronzo. Is the ADA/EASD algorithm for the management of type 2 diabetes (January 2009) based on evidence or opinion? A critical analysis. Diabetologia 2010; 53:1258–1269.
17. Defronzo RA. Banting Lecture. From the triumvirate to the ominous octet: a new paradigm for the treatment of type 2 diabetes mellitus. Diabetes 2009;58:773-95.
18. Russell S. Incretin-based therapies for type 2 diabetes mellitus: a review of direct comparisons of efficacy, safety and patient satisfaction. Int J Clin Pharm. 2013 Apr;35(2):159-72.
19. Wu D, Li L, Liu C. Efficacy and safety of dipeptidyl peptidase-4 inhibitors and metformin as initial combination therapy and as monotherapy in patients with type 2 diabetes mellitus: a meta-analysis. Diabetes Obes Metab. 2013 Jun 26. doi:10.1111/dom.12174. [Epub ahead of print] PubMed PMID: 23803146
20. Scirica BM, Bhatt DL, Braunwald E, Steg PG, Davidson J, Hirshberg B, Ohman P, Frederich R, Wiviott SD, Hoffman EB, Cavender MA, Udell JA, Desai NR, Mosenzon O, McGuire DK, Ray KK, Leiter LA, Raz I; the SAVOR-TIMI 53 Steering Committee and Investigators. Saxagliptin and Cardiovascular Outcomes in Patients with Type 2 Diabetes Mellitus N Engl J Med. 2013 Oct 3;369(14):1317-1326.
21. White WB, Cannon CP, Heller SR, Nissen SE, Bergenstal RM, Bakris GL, Perez AT, Fleck PR, Mehta CR, Kupfer S, Wilson C, Cushman WC, Zannad F; the EXAMINE Investigators. Alogliptin after Acute Coronary Syndrome in Patients with Type 2 Diabetes N Engl J Med. 2013 Oct 3;369(14):1327-1335
22. Scheen AJ. Cardiovascular effects of dipeptidyl peptidase-4 inhibitors: from risk factors to clinical outcomes. Postgrad Med. 2013 May;125(3):7-20..
23. Richard KR, Shelburne JS, Kirk JK. Tolerability of dipeptidyl peptidase-4 inhibitors: a review. Clin Ther. 2011 Nov;33(11):1609-29.
24. Williams-Herman D, Engel SS, Round E, Johnson J, Golm GT, Guo H, Musser BJ, Davies MJ, Kaufman KD, Goldstein BJ. Safety and tolerability of sitagliptin in clinical studies: a pooled analysis of data from 10,246 patients with type 2 diabetes. BMC Endocr Disord. 2010 Apr 22;10:7:1606-16

- Primary endpoint assessments:

25. Early Treatment Diabetic Retinopathy Study Research Group (ETDRS). Fundus photographic risk factors for progression of diabetic retinopathy. ETDRS report no. 12. Ophthalmology 1991;83:823–833.
26. Tesfaye S , Boulton AJ, Dyck PJ, Freeman R, Horowitz M, Kempler P, Lauria G, Malik RA, Spallone V, Vinik A, Bernardi L, Valensi P; Toronto Diabetic Neuropathy Expert Group. Diabetic neuropathies: update on definitions, diagnostic criteria, estimation of severity, and treatments. Diabetes Care 2010; 33:2285-93.
27. H. Gin, R. Baudouin, C. Raffaitin, V. Rigalleau, C. Gonzalez. Non invasive and quantitative assessment of sudomotor function for peripheral diabetic neuropathy evaluation. *Diabetes & Metabolism*. 2011;11(4):527-32.
28. H. Mayaudon, PO. Miloche, B. Bauduceau. A new simple method to assess sudomotor function: interest in type 2 diabetes. *Diabetes & Metabolism.* 2010;36(6):450-4.
29. Earley A, Miskulin D, Lamb EJ, Levey AS, Uhlig K. Estimating equations for glomerular filtration rate in the era of creatinine standardization: a systematic review. Ann Intern Med. 2012 Jun 5;156(11):785-95.

- A process model for supporting lifestyle behaviour change

30. Greaves, C., Reddy, P. and Sheppard, K. 2010. Supporting Behaviour Change for Diabetes Prevention. In: Schwarz, P., Reddy, P., Greaves, C., Dunbar, J. and Schwarz J. (eds.) Diabetes Prevention in Practice. Dresden: Tumaini Institute for Prevention Management, 19-29

- Other relevant references:

31. Lindström J, Tuomilehto J. The diabetes risk score: a practical tool to predict type 2 diabetes risk. Diabetes Care. 2003;26:725-31.
32. Pajunen P, Peltonen M, Eriksson JG, Ilanne-Parikka P, Aunola S, et al. Finnish Diabetes Prevention Study. HbA(1c) in diagnosing and predicting Type 2 diabetes in impaired glucose tolerance: the Finnish Diabetes Prevention Study. Diabet Med. 2011 Jan;28(1):36-42.
33. Golubic R, Ekelund U, Wijndaele K, Luben R, Khaw KT, et al. Rate of weight gain predicts change in physical activity levels: a longitudinal analysis of the EPIC-Norfolk cohort. Int J Obes (Lond). 2012 Apr 24
34. Domínguez LJ, Bes-Rastrollo M, de la Fuente-Arrillaga C, Toledo E, Beunza JJ, Barbagallo M, Martínez-González MA. Similar prediction of decreased total mortality, diabetes incidence or cardiovascular events using relative- and absolute-component Mediterranean diet score: The SUN cohort. Nutr Metab Cardiovasc Dis. 2012 Mar 6.
35. Hamburg NM, Keyes MJ, Larson MG, Vasan RS, Schnabel R, Pryde MM, Mitchell GF, Sheffy J, Vita JA, Benjamin EJ. Cross-sectional relations of digital vascular function to cardiovascular risk factors in the Framingham Heart Study. *Circulation*. 2008;117:2467–2474
36. Vanoli D, Lindqvist P, Wiklund U, Henein M, Näslund U. Fully automated on-screen carotid intima-media thickness measurement: a screening tool for subclinical atherosclerosis. J Clin Ultrasound. 2013 Jul-Aug;41(6):333-9
37. Florez H, Pan Q, Ackermann RT, Marrero DG, Barrett-Connor E, Delahanty L, et al. Diabetes Prevention Program Research Group. Impact of lifestyle intervention and metformin on health-related quality of life: the diabetes prevention program randomized trial. J Gen Intern Med. 2012
38. Ziegler D, Rathmann W, Dickhaus T, Meisinger C, Mielck A; KORA Study Group. Prevalence of polyneuropathy in pre-diabetes and diabetes is associated with abdominal obesity and macroangiopathy: the MONICA/KORA Augsburg Surveys S2 and S3. Diabetes Care. 2008 Mar;31(3):464-9.
39. Tovilla-Zárate C, Juárez-Rojop I, Peralta Jimenez Y, Jiménez MA, Vázquez S, et al. Prevalence of anxiety and depression among outpatients with type 2 diabetes in the Mexican population. PLoS One.2012;7(5):e36887.
40. Maia AC, Braga Ade A, Brouwers A, Nardi AE, Oliveira e Silva AC. Prevalence of psychiatric disorders in patients with diabetes types 1 and 2. Compr Psychiatry. 2012 Nov;53(8):1169-73
41. Chen RH, Jiang XZ, Zhao XH, Qin YL, Gu Z, Gu PL, et al. Risk factors of mild cognitive impairment in middle aged patients with type 2 diabetes: a cross-section study. Ann Endocrinol (Paris). 2012 Jun;73(3):208-12.
42. Guo X, Zhang Z, Zhu W, Lian N, Lu H, Zhao J. Cognitive functioning in schizophrenia with or without diabetes. Zhong Nan Da Xue Xue Bao Yi Xue Ban. 2011 Aug;36(8):724-7.
43. Spießhöfer J, Heinrich J, Bitter T, Efken C, Lehmann R, Eckert S, Horstkotte D, Oldenburg O. Validation of blood pressure monitoring using pulse transit time in heart failure patients with Cheyne-Stokes respiration undergoing adaptive servoventilation therapy. Sleep Breath. 2013 Sep 24.
44. Mari A, Ferrannini E. Beta-cell function assessment from modelling of oral tests: an effective approach. Diabetes
45. Obes Metab. [Review Validation Studies]. 2008 Nov;10 Suppl 4:77-87

Appendix A: Visit plan (eliminated)

(See protocol V5.0)

Appendix B: Description of drug products characteristics released by the European Medicines Agency

(Independent documents):

EMA_jentadueto.pdf:

http://www.ema.europa.eu/docs/en_GB/document_library/EPAR_-_Product_Information/human/002279/WC500130969.pdf

EMA_trajenta.pdf

http://www.ema.europa.eu/docs/en_GB/document_library/EPAR_-_Product_Information/human/002110/WC500115745.pdf

EMA_glucophage.pdf

http://www.ema.europa.eu/docs/en_GB/document_library/Referrals_document/Glucophage_30/WC500011044.pdf

Appendix C: Sample of informed consent document.

Independent document.

e-predice_Annex_ICF_V 5.1 June 12 2018
