# Supplementary material for: Early prevention of diabetes microvascular complications in people with hyperglycaemia in Europe. ePREDICE randomized trial. Study protocol, recruitment and selected baseline data
Source: PLoS One. 2020 Apr 13;15(4):e0231196. doi: 10.1371/journal.pone.0231196 (PMC7153858; doi:10.1371/journal.pone.0231196)
Supplement: S3 Data — (PDF) [file pone.0231196.s007.pdf]

3 January 2015

Complementary description of statistical power, ePREDICE study

To determine to what extent the power changes in the ePREDICE-study if the smple size is reduced additional power calculations have been performed. Anders Odén, Professor in statistics at the Chalmers Technology University, University of Gothenburg, Gothenburg, Sweden, participant of the statistical work package, has performed the calculations. Professor Odén was also involved in the original power calculations for the ePREDICE trial. Below is shown how the statistical power varies for various sample sizes.

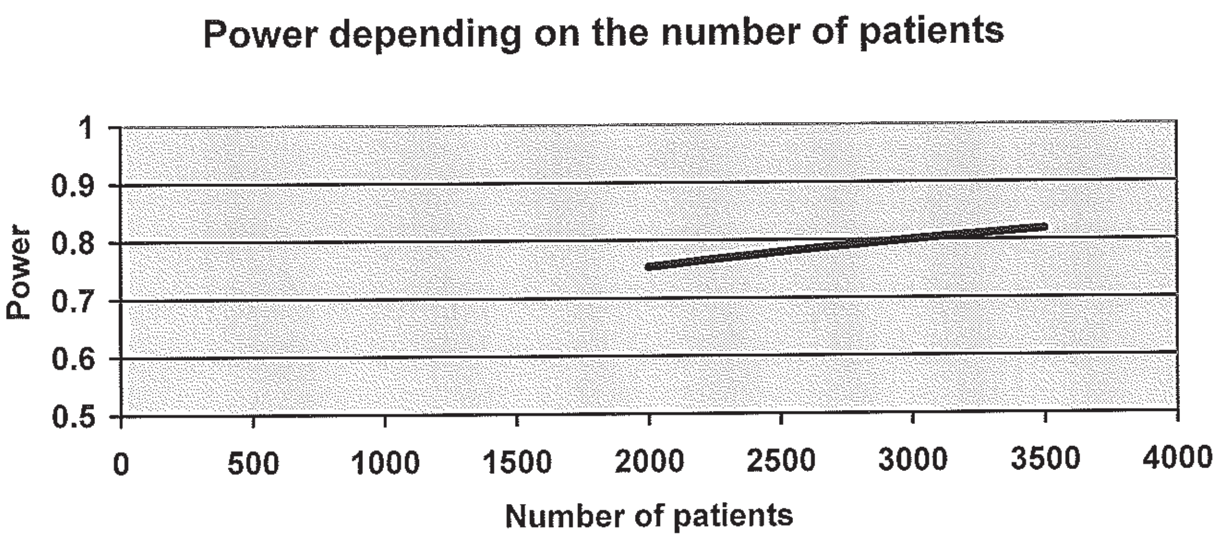

Let Φ denote the standardized normal distribution function. The power, when the size of the study deviates from 3000 could be calculated as

$\Phi(0.8417 \cdot (n/3000)^{1/2})$,

where n is the number of patients. When the number of patients is reduced from 3000 to 2000 the power decreases from 80% to 75.4 %, which is not a substantial change.

Dr Marcus Lind
Coordinator of the statistical work package

Associate Professor of Diabetology
University of Gothenburg, Gothenburg, Sweden

Chief Physician of Diabetology
Research responsible, department of Medicine
NU-Hospital Organization, Uddevalla, Sweden

ANNEX. SAMPLE SIZE AND STATISTICAL POWER CALCULATIONS

In the original study protocol power calculations were done for incidences of events, but in order to achieve the maximum efficiency the primary analyses will be performed by use of the corresponding continuous variables.

The primary comparison will be between the group with life-style intervention and the union of the other treatment groups (500 versus 1500 patients) with respect to a score. The power of non-parametric tests will be approximately the same as for t-test. If Dm/s = 0.12 then the power 80%, two-sided test at the significance level 0.05, will be achieved. When two treatment groups are compared (750 versus 750 patients) the same power will be achieved if Dm/s = 0.15. The difference between the two frequency functions is 0.15 times the standard deviation.
The expected 95% confidence interval if there is no difference between the groups is -0.10 - 0.10 times the standard deviation. That means that the sample size is sufficient to assess that the difference is small, if that is the case.

We have no empirical results of efficacy of the treatments for participants with pre-diabetes on the studied condition (micro and macrovascular complications). Thus we have chosen an efficient variable (instead of relatively rare events) for the comparison and considered so small differences that smaller ones will be of limited value to treat. Thereby we try to avoid a situation where we neither reach a significant result nor can assess that the difference between treatments are small. We can simply say that the study is dimensioned so it will be conclusive.
Events versus a continuous variable

When studying the effect of a treatment compared with another one we can either use the event of getting the diagnosis during the study period or we can perform the comparison between the treatments with respect to the change or the final value of the continuous variable. The latter type of comparison turns in many situations out to be much more efficient than the comparison of the incidence of events. A basic scientific principle tells us that we should apply the most powerful type of comparison even if it is of interest (as a later step) to elucidate the effect in terms of hazard functions of events.

Only at the end of the study X is observed. If X>1.28 (the cut off limit) an event has occurred. The probability of event is 0.10 for control group and 0.0604 for the new treatment group. In order to achieve the power 80% at the significance level 0.05, two-tailed test, 2×750 patients are needed (Fisher's exact test).

The use of continuous variables in this case is not only more powerful due to the reasons shown above but also for another reason. The development of a complication to IGT or IFG, will probably take a long time and the change of the corresponding continuous variable will be a much earlier sign and thus the difference in power is probably even larger than shown above.

As a primary aim we will compare a linear combination of a retinopathy scale, albumin to creatinine ratio and a score of neuropathy (sweat function). The coefficients of the linear combination will be determined on the basis of information from other materials (published and unpublished) available before the analysis of this study so the expected value of the power determining quantity will be as large as possible. One of the coefficients could without loss of efficiency be put to 1, so there are two of them to be determined. Because other materials than that from the present study are used, no bias is introduced by the optimization. As further aims we will analyze the risk of the different events depending on treatment and other variables.

The determination of the coefficients will not use information from this present study, therefore will not be possible to manipulate these coefficient based on results. The coefficients will be determined during the study when enough information from suitable other studies have been found. The determination of the coefficients will at least be defined on before the database is closed and locked, based on all information available at that time point.
